# Identification of Hub Genes Associated With Progression and Prognosis in Patients With Bladder Cancer

**DOI:** 10.3389/fgene.2019.00408

**Published:** 2019-05-07

**Authors:** Xin Yan, Xiao-Ping Liu, Zi-Xin Guo, Tong-Zu Liu, Sheng Li

**Affiliations:** ^1^Department of Urology, Zhongnan Hospital of Wuhan University, Wuhan, China; ^2^Department of Biological Repositories, Zhongnan Hospital of Wuhan University, Wuhan, China; ^3^Human Genetics Resource Preservation Center of Hubei Province, Wuhan, China

**Keywords:** bladder cancer, hub genes, survival analysis, enrichment analysis, prognosis, bioinformatics

## Abstract

Given that most bladder cancers (BCs) are diagnosed in advanced stages with poor prognosis, this study aims to find novel biomarkers associated with the progression and prognosis in patients with BC. 1,779 differentially expressed genes (DEGs) between BC samples and normal bladder tissues were identified in total. Then, 24 DEGs were regarded as candidate hub genes by constructing a protein–protein interaction (PPI) network and a random forest model. Among them, six genes (BUB1B, CCNB1, CDK1, ISG15, KIF15, and RAD54L) were eventually identified by using five analysis methods (one-way Analysis of Variance analysis, spearman correlation analysis, distance correlation analysis, receiver operating characteristic curve, and expression values comparison), which were correlated with the progression and prognosis of BC. Moreover, the validation of hub genes was conducted based on GSE13507, Oncomine, and CBioPortal. Results of univariate Cox regression analysis showed that the expression levels of all the hub genes were influence features of overall survival (OS) and cancer specific survival (CSS) based on GSE13507, and we further established a six-gene signature based on the expression levels of the six genes and their Cox regression coefficients. This signature showed good potential for clinical application suggested by survival analysis (OS: Hazard Ratio = 0.484, 95%CI: 0.298–0.786; *P* = 0.0034; CSS: Hazard Ratio = 0.244, 95%CI: 0.121–0.493, *P* < 0.0001) and decision curve analysis. In conclusion, our study indicates that six hub genes have great predictive value for the prognosis and progression of BC and may contribute to the exploration of further basic and clinical research of BC.

## Introduction

Bladder cancer (BC) is the most common malignancy of the urinary system (Ferlay et al., 2010). According to recent research, the incidence of BC is growing worldwide (Ebrahimi et al., 2019). There were 437,442 new cases of BC in 2016 (Ebrahimi et al., 2019). BC ranks the first in urinary malignancies among males (Chen et al., 2016). Therefore, early diagnosis, postoperative monitoring, prognosis evaluation and more effective individualized treatment of BC are extremely important. At present, cystoscopy and biopsy are still the gold standard for diagnosing BC (Emerson and Cheng, 2005). Cystoscopy is an invasive examination, which is not accepted by most patients (Harris et al., 1997). Urinary cytology examination is the main method for diagnosing BC and postoperative follow-up (Matsuzaki et al., 2016). The specificity of urinary cytology examinations was high, ranging from 90 to 96%, while the sensitivity of diagnosis for low grade and early stage BC was very low (Kiyoshima et al., 2016). Biomarkers are the frontiers and hotspots in the screening and diagnosis of BC (Kiyoshima et al., 2016).

In recent years, a few biomarkers have been used for the diagnosis of BC. For instance, Nuclear Matrix Protein-22 (NMP-22), a protein component found in the spindle of mitotic cells, could ensure chromosome segregation when cells underwent mitosis. A small amount of NMP-22 could be found in the urine of normal people, and BC might exist when its content exceeded the threshold (Miakhil et al., 2013). However, the sensitivity and specificity of NMP-22 were reported controversially in literatures (47–90%) (Tilki et al., 2011). Given that most BCs were diagnosed with advanced stages, the prognosis of patients with BC remained extremely poor. Therefore, it is of top priority to develop novel and specific prognostic markers for patients with BC.

In the field of machine learning and data mining, random forest is an ensemble learning method for classification, regression and other tasks (Rigatti, 2017). Random forest realizes its function by constructing a multitude of decision trees at training time (Svetnik et al., 2003). Then, it further outputs the class that is the mode of the classification or mean prediction (regression) of the individual trees (Svetnik et al., 2003). This method has a lot of advantages, one of these is that it gives estimates of what variables are important in the classification (Svetnik et al., 2003), which interests us most. It means that we can construct a random forest model to narrow down the number of genes (variables) according to the importance of variables.

Previous studies only identified differentially expressed genes (DEGs) based on one database and there was a lack of validation when using other databases (Jia et al., 2015). Although some studies had validated their results through reverse transcription-quantitative polymerase chain reaction (RT-qPCR) and western blotting, researchers only identified hub genes by constructing a co-expression network (Zhang et al., 2017) or a protein–protein interaction (PPI) network (Bi et al., 2015). In this study, we not only constructed a PPI network of DEGs to pick out hub genes, but also innovatively constructed a random forest model to further narrow down the number of hub genes in the PPI network by using these genes as features and their expression levels as feature values. Furthermore, unlike other studies, we used many different methods to identify hub genes associated with the progression and prognosis of patients with BC among these candidate hub genes and further validated them based on other databases.

## Materials and Methods

### BC Gene Expression Studies

To screen DEGs, we downloaded TCGA-BLCA data including 413 BCs with clinical information and 19 normal bladder samples from The Cancer Genome Atlas (TCGA) database^1^. Another independent dataset GSE13507 (Kim et al., 2010; Lee et al., 2010) was downloaded from the gene expression omnibus (GEO) database^2^ to verify our results. GSE13507, an expression profile based on Agilent GPL6102 platform (Illumina Human-6 v2.0 Expression Beadchip), included 165 primary BC samples, 23 recurrent non-muscle invasive tumor tissues, 10 normal bladder mucosae and 58 bladder mucosae surrounding cancer.

### Data Processing and DEG Screening

**FIGURE 1:**
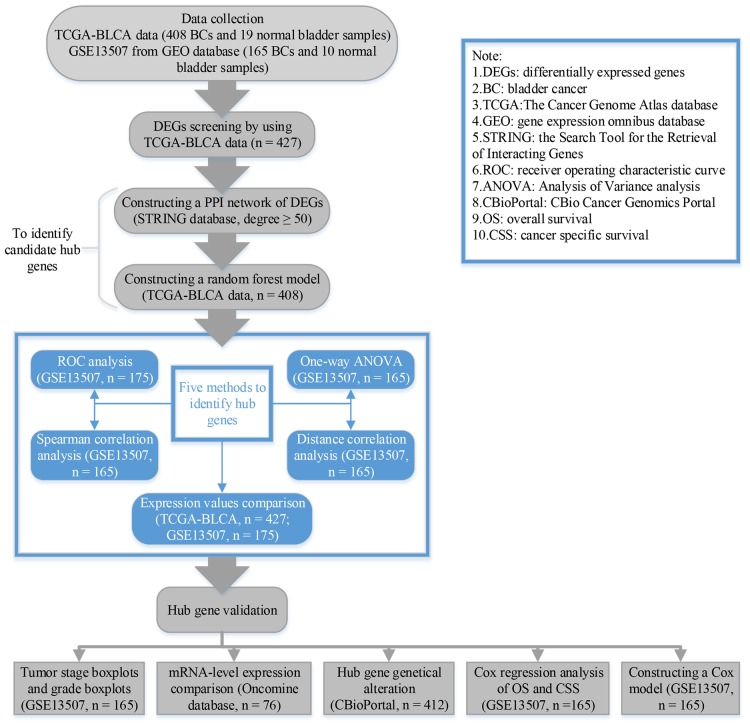
Flow diagram of data preparation, processing, analysis, and validation in this study.

The research process of this study was showed in Figure 1. For GSE13507, we used “affy” in R for normalization and log2 transformation by using “rma” algorithm (Gautier et al., 2004). After weeding out samples with incomplete clinical information, a total of 427 samples (408 BCs and 19 normal samples) were used to select DEGs by using package “edgeR” (Robinson et al., 2010) in R. We considered genes as DEG when they reached the standard: Adjust *P* value < 0.05, and | log_2_ fold change (FC)|≥ 2 (Sun et al., 2017; Li et al., 2018).

### Functional Enrichment Analysis

We performed Gene Ontology (GO) enrichment analysis and Kyoto Encyclopedia of Genes and Genomes (KEGG) pathway analysis for DEGs to find out their lurking functions by using R package “clusterProfiler” (Yu et al., 2012). In this study, we only showed the results of biological process (BP) and KEGG. Gene sets at *P* < 0.05 were considered to be significantly enriched (Li et al., 2018).

### Candidate Hub Gene Identification

Firstly, by means of the Search Tool for the Retrieval of Interacting Genes (STRING) (Szklarczyk et al., 2015), we built the PPI network of DEGs. Parameters setting: Network scoring: degree cutoff = 2; Cluster finding: node score cutoff = 0.2, k-core = 2, and max. depth = 100 (Sun et al., 2017). In this study, we calculated the degree of genes by network analyzer (a tool in Cytoscape, Shannon et al., 2003). After that, genes with degree ≥ 50 were considered to be hub genes in the PPI work. Secondly, in order to pick out the most important factors associated with the progression among them, we further constructed a random forest model of hub genes in the PPI network by using package “randomForest” (Liaw and Wiener, 2002) in R. After that, genes which reached the standards (both of MeanDecreaseAccuracy and MeanDecreaseGini ranked top 50) (Svetnik et al., 2003) were considered as candidate hub genes.

### Hub Gene Identification

In this study, five different methods were used to identify hub genes among candidate hub genes using GEO dataset GSE13507. The one-way ANOVA test and spearman correlation analysis were performed using SPSS (version 21.0). We used R package “ggplot2” (Wickham, 2015) to visualize the results. Meanwhile, we used R package “energy” (Rizzo and Szekely, 2016) to complete the distance correlation analysis to overcome the weaknesses of spearman correlation. All of the three analyses were performed to explore the correlation between gene expression levels and tumor grade to pick out genes associated with tumor progression based on GSE13507. Moreover, by means of R package “plotROC” (Sachs, 2015), Receiver operating characteristic curve (ROC) analysis was performed. In GSE13507, we worked out the AUC to distinguish BC samples from normal tissues. After that, we compared the expression levels of candidate hub genes in BC and normal bladder tissues using GSE13507 (*n* = 175) and TCGA-BLCA data (*n* = 427). The boxplots were drawn using R package “ggstatsplot” (Patil and Powell, 2018) and GEPIA (Tang et al., 2017) (Gene Expression Profiling Interactive Analysis). Genes which satisfied the standard (*P* < 0.05 in all analysis and AUC ≥ 0.85) (Yuan et al., 2018) were considered to be hub genes. An upset plot was also performed using R package “UpSetR” (Conway et al., 2017) to overlap genes in these five analysis methods.

### Hub Gene Validation and Genetical Alteration

Based on GSE13507, T stage (Ta, T1, T2, T3, and T4) boxplots and tumor grade (low and high) boxplots were performed using “ggstatsplot.” A one-way ANOVA test was performed to measure the statistical significance in stage boxplots. In addition, we validated the mRNA-level expression of hub genes based on Oncomine^3^ (Rhodes et al., 2004). In the present study, 28 tumors and 48 normal bladder samples from Sanchez-Carbayo Bladder 2 were included (Sanchez-Carbayo et al., 2006). We used an unpaired *t*-test to measure the statistical significance in grade boxplots and mRNA-level validation. Visualization and analysis of cancer genomic datasets can be realized by using the CBio Cancer Genomics Portal^4^ (Cerami et al., 2012; Gao et al., 2013). In the present study, CBioPortal was used for exploring genetic alterations of hub genes and relationships between genes and drugs (the data of CbioPortal originated from TCGA, 412 samples in total were included in this step).

### Univariate and Multivariate Cox Regression Analysis

In the present study, we included the hub gene expression values and other important clinical information (gender, age, tumor grade, T stage, N stage, and M stage) into univariate Cox analysis of overall survival (OS) by using dataset GSE13507. After that, we constructed a Cox model by combining the regression coefficient (beta) with gene expression values. The Cox model and factors with *p*-Value < 0.05 in univariate Cox analysis were included for multivariate Cox analysis. To do this, we could determine if the prediction of hub genes was independent from other clinical features. We used R package “forestplot” (Aut and Aut, 2016) for visualization.

### Survival Analysis and Decision Curve Analysis (DCA)

For investigating the influence of the Cox model on the OS and cancer specific survival (CSS) of BC patients, we calculated the risk score of every sample in GSE13507. BCs were divided into two groups (high risk and low risk) according to the median risk score of GSE13507. Kaplan–Meier survival analysis was conducted with the information from GSE13507 by using R package “survival” (Therneau, 2015). This package also generated a Kaplan–Meier survival curve. In addition, DCA was used to further validate the diagnostic value of this model and hub genes.

## Results

### Identification of DEGs Between BC and Normal Bladder Tissues

In total, 1, 779 DEGs were identified under the threshold of adjust *P-*value < 0.05 and | log_2_FC|≥ 2. Among them, 908 genes were up-regulated and 871 genes were down-regulated (Supplementary Figure S1). The adjust *P*-value and log_2_FC of DEGs were available in Supplementary Table S1.

### GO Biological Processes and KEGG Analysis

**FIGURE 2:**
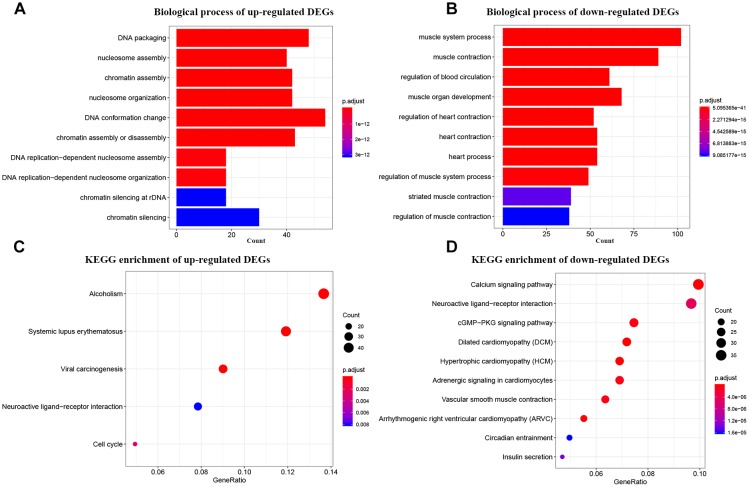
Bioinformatics analysis of up-regulated and down-regulated DEGs. **(A)** GO analysis of up-regulated DEGs. **(B)** GO analysis of down-regulated DEGs. **(C)** KEGG pathway enrichment of up-regulated DEGs. **(D)** KEGG pathway enrichment of down-regulated DEGs.

For primary comprehensions of these DEGs, GO and KEGG pathway analyses were performed. According to GO biological processes analysis, the up-regulated DEGs were enriched in 102 BPs. The top 10 were DNA packaging, nucleosome assembly, chromatin assembly, nucleosome organization, DNA conformation change, chromatin assembly or disassembly, DNA replication-dependent nucleosome assembly, DNA replication-dependent nucleosome organization, chromatin silencing at rDNA, and chromatin silencing (Figure 2A). As for the down-regulated DEGs, they were enriched in 326 BPs in total. The top 10 were muscle system process, muscle contraction, regulation of blood circulation, muscle organ development, regulation of heart contraction, heart contraction, heart process, regulation of muscle system process, striated muscle contraction, and regulation of muscle contraction (Figure 2B). While in KEGG pathway analysis, the up-regulated DEGs were enriched in only 5 KEGG pathways including systemic lupus erythematosus, alcoholism, viral carcinogenesis, cell cycle, and neuroactive ligand-receptor interaction (Figure 2C). Meanwhile, the down-regulated DEGs were enriched in 33 KEGG pathways totally. The top 10 were dilated cardiomyopathy (DCM), hypertrophic cardiomyopathy (HCM), calcium signaling pathway, arrhythmogenic right ventricular cardiomyopathy (ARVC), vascular smooth muscle contraction, cGMP-PKG signaling pathway, adrenergic signaling in cardiomyocytes, neuroactive ligand-receptor interaction, insulin secretion, and circadian entrainment (Figure 2D).

### Candidate Hub Gene Identification

**FIGURE 3:**
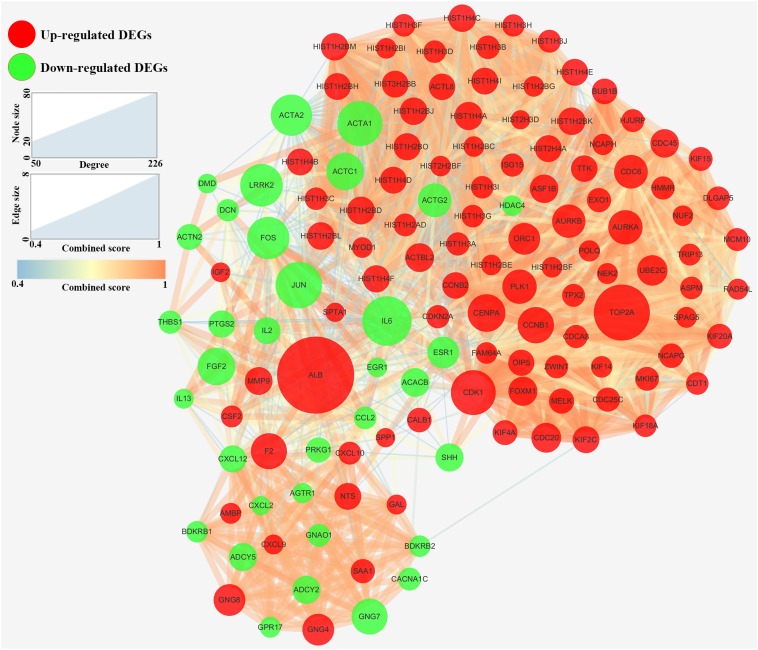
Protein–protein interaction network of hub genes (degree ≥ 50) in DEGs. Red nodes: Up-regulated DEGs. Green nodes: Down-regulated DEGs. The node size was proportional to the degree and the edge width was proportional to the combined score based on STRING database.

At the beginning, we constructed a PPI network of DEGs (Supplementary Figure S2). 134 genes with degree ≥ 50 were considered as hub genes in the PPI network (Figure 3). In order to narrow down the number of hub genes in the PPI network, we also constructed a random forest model. According to the results, 24 genes were common in genes with MeanDecreaseAccuracy ranked in the top 50 and genes with MeanDecreaseGini ranked in the top 50 (Supplementary Table S2). Genes with MeanDecreaseAccuracy ranked in the top 30 and genes with MeanDecreaseGini ranked in the top 30 were shown in Supplementary Figure S3. We regarded these 24 genes as candidate hub genes for further analysis.

### Hub Gene Identification

When exploring the correlation between gene expression levels and tumor grade, 12 genes were identified by one-way ANOVA, 22 genes were picked out by the spearman correlation analysis, 24 genes were screened by the distance correlation analysis (*P* < 0.05, Supplementary Table S3). Then, we performed ROC curve analysis, eight genes which reached the standard of AUC ≥ 0.85 were finally screened out (Supplementary Table S3). In addition, 16 genes were differentially expressed in tumor tissues compared with normal tissues based on GSE13507.

**FIGURE 4:**
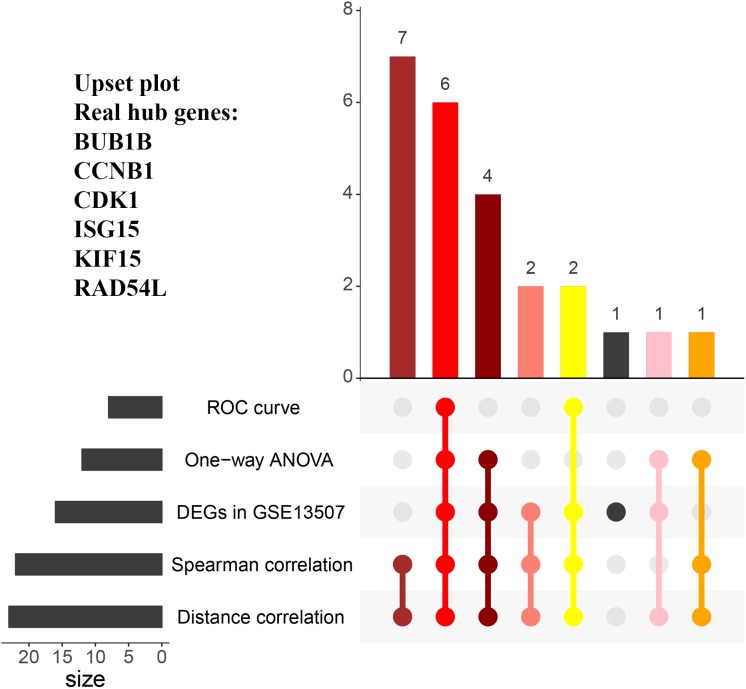
The UpSet intersection diagram to identify hub genes. Five types of analyses were showed in the UpSet plot. The numbers on the bars stand for the numbers of significative genes in the corresponding analyses.

**FIGURE 5:**
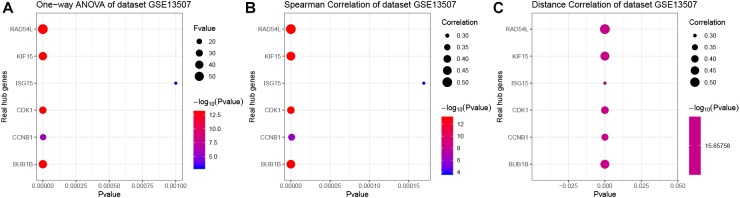
The correlation analysis between hub gene expression levels and tumor grade. **(A)** One-way ANOVA analysis of the hub genes using GSE13507. **(B)** Spearman correlation analysis of the hub genes using GSE13507. **(C)** Distance correlation analysis of the hub genes using GSE13507. Hub genes: BUB1B, CCNB1, CDK1, ISG15, KIF15, and RAD54L.

**FIGURE 6:**
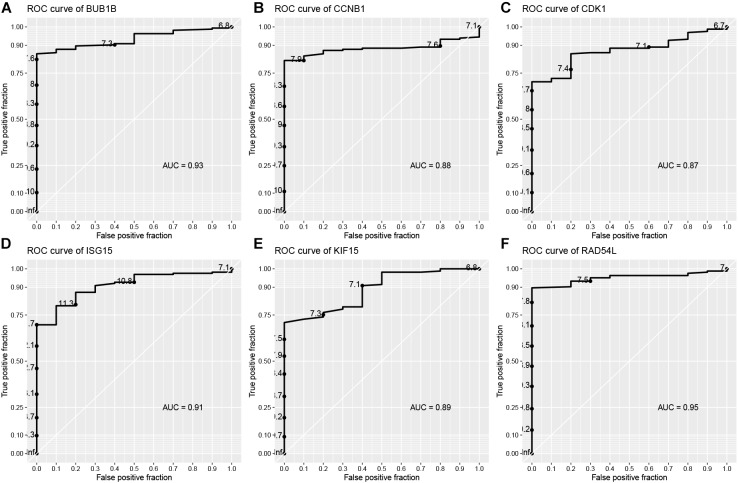
Receiver operating characteristic (ROC) curves and area under the curve (AUC) statistics to evaluate the diagnostic efficiency of the hub genes in GSE13507. **(A)** BUB1B, **(B)** CCNB1, **(C)** CDK1, **(D)** ISG15, **(E)** KIF15, and **(F)** RAD54L.

In the end, six genes [BUB1B (BUB1 mitotic checkpoint threonine kinase B), CCNB1 (cyclin B1), CDK1 (cyclin-dependent kinase 1), ISG15 (Interferon-stimulated gene 15 kDa protein), KIF15 (Kinesin family member15), and RAD54L (RAD54 like)] were identified as hub genes, because they showed significant *P*-value in all the five analysis procedures (Figure 4). As shown in Figure 5, the six hub genes were all significantly associated with the progression of BC. Among them, RAD54L might be the biggest factor affecting tumor grade (one-way ANOVA: 56.778, *P* < 0.001; spearman correlation: 0.504, *P* < 0.001; distance correlation: 0.522, *P* < 0.001). The results of ROC curve indicated that RAD54L could distinguish BC samples from normal tissues best, among all the hub genes (BUB1B: AUC = 0.934; CCNB1: AUC = 0.884; CDK1: AUC = 0.869; ISG15: AUC = 0.908; KIF15: AUC = 0.888; and RAD54L: AUC = 0.951, Figure 6). Moreover, based on GSE13507 and TCGA-BC data, the expression levels of these six genes were significantly higher in tumor tissues (Supplementary Figure S4).

### Validation and Genetical Alteration of Hub Genes

**FIGURE 7:**
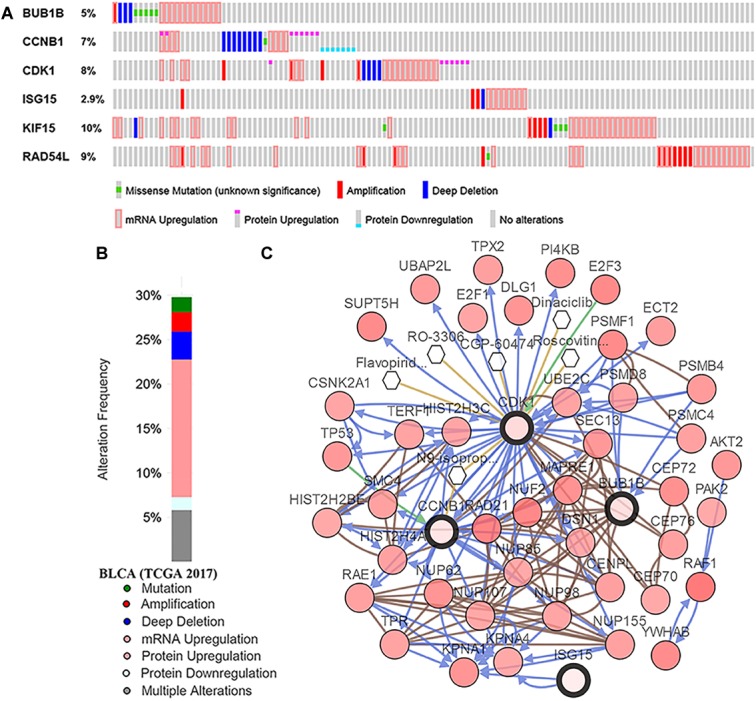
Genetic alterations associated with hub genes in TCGA-BLCA. **(A)** A visual summary across on a query of six hub genes showing genetic alteration of six hub genes in TCGA-BLCA patients. **(B)** The total alteration frequency of six hub genes in TCGA-BLCA is illustrated. **(C)** The network contains 46 genes (4 hub genes and 42 most variant genes). Relationship between hub genes and tumor drugs is also illustrated. BLCA, bladder urothelial carcinoma.

Based on GSE13507, the tumor grade and stage boxplots of six hub genes were shown in Supplementary Figures S5A,B. The results suggested that all the hub genes were significantly associated with the grade and T stage of tumor. In addition, mRNA expression levels were all significantly higher in tumor tissues compared with those in normal tissues (BUB1B: *t* = -8.109, *P* < 0.001; CCNB1: *t* = -9.942, *P* < 0.001; CDK1: *t* = -9.784, *P* < 0.001; ISG15: *t* = -4.008, *P* < 0.001; KIF15: *t* = -3.781, *P* < 0.001; and RAD54L: *t* = -2.944,*P* = 0.005; Supplementary Figure S5C), which was suggested by the Oncomine database. These results made the six hub genes we screened out reliable. As for genetical alteration, six hub genes were altered in 123 (30%) of 412 patients (Figure 7B). As shown in Figure 7A, KIF15 altered most (10%) and the main type was mRNA upregulation. A network contained 46 genes (4 hub genes and 42 most variant genes) was showed in Figure 7C. As for the relationship between anticancer drugs and hub genes, we found CCNB1 and CDK1 were the targets of cancer drugs. But there was no drug targeting to the rest four hub genes, which might be novel therapeutic targets for patients with BC.

### Cox Regression Analysis of OS and CSS Among Patients With BC

**FIGURE 8:**
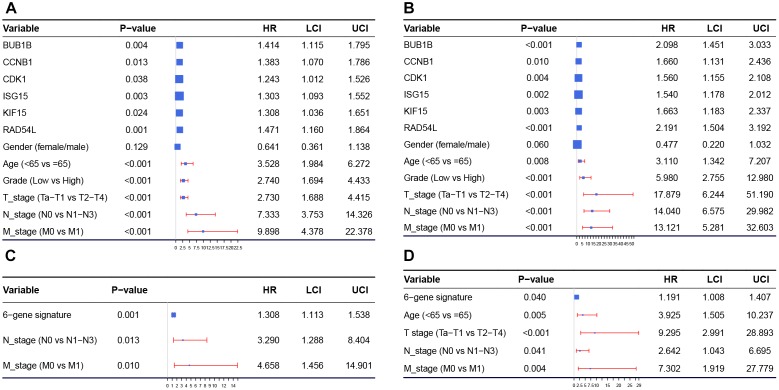
Forest plot summary of analyses of OS **(A)** and CSS **(B)** univariable analyses of hub genes, gender, age, grade and TNM stage by using GSE13507. Forest plot summary of analyses of OS **(C)** and CSS **(D)** multivariable analyses of the six-gene signature and other influence features by using GSE13507. HR, hazard ratio; OS, overall survival; CSS, cancer specific survival.

Univariate Cox regression analysis showed that almost all the factors we included were influence features of OS and CSS, except gender (Figure 8A,B). Following this we established a six-gene signature, with the risk scores calculated based on the expression levels of the six genes and Cox regression coefficients as follows: risk score of OS = BUB1B × 0.347 + CCNB1 × 0.324 + CDK1 × 0.218 + ISG15 × 0.264 + KIF15 × 0.268 + RAD54L × 0.386, risk score of CSS = BUB1B × 0.741 + CCNB1 × 0.507 + CDK1 × 0.445 + ISG15 × 0.432 + KIF15 × 0.508 + RAD54L × 0.784. After that, we included the six-gene signature into the multivariate Cox analysis. The results showed that even being adjusted by other factors, risk scores of the six-gene signature were still relevant to OS and CSS among patients with BC (Figure 8C,D).

### Survival Analysis of Real Hub Genes and DCA

**FIGURE 9:**
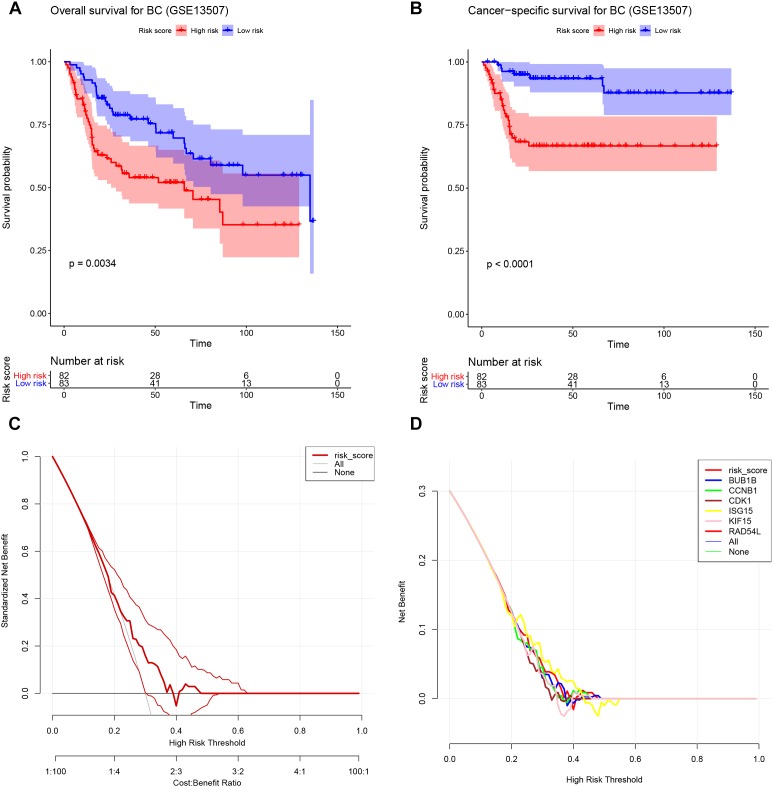
Validation of the Cox model. **(A)** Survival analysis of the association between risk score and overall survival time in BC (based on GSE13507). **(B)** Survival analysis of the association between risk score and cancer-specific survival time in BC (based on GSE13507). **(C)** DCA for assessment of the clinical utility of the six-gene signature. **(D)** DCA for assessment of the clinical utility of BUB1B, CCNB1, CDK1, ISG15, KIF15, and RAD54L. The *x*-axis represents the percentage of threshold probability, and the *y*-axis represents the net benefit. DCA, decision curve analysis.

One hundred sixty-five BC patients’ prognostic information was obtained from GSE13507. The result suggested that the high-risk group (Hazard Ratio = 0.484, 95%CI of ratio: 0.298–0.786, *P* = 0.0034) had worse OS for patients with BC (Figure 9A). As for CSS analysis, the high-risk group (Hazard Ratio = 0.244, 95%CI of ratio: 0.121–0.493, *P* < 0.0001) was obviously associated with poor CSS for patients with BC (Figure 9B), as shown in Figure 9C, except when then value of Threshold Probability (Pt) = 0.40; this signature showed high potential, because the Pt (the red thick line in the figure) ensured better net benefits compared with all (gray line in the figure), or none, of the options (black line in the figure). But we could not distinguish this signature from the single gene models because it did not ensure better net benefits compared with others (Figure 9D).

## Discussion

Bladder cancer, which is among the leading causes of cancer death globally, can occur at any age (Ebrahimi et al., 2019). Therefore, there is a pressing need for sensitive and novel biomarkers of BC.

After determining our goals, we identified 1,779 DEGs in total. These DEGs were made up of 908 up-regulated genes and 871 down-regulated genes. According to the GO biological processes analysis, the up-regulated DEGs majorly participated in DNA packaging, nucleosome assembly, chromatin assembly, nucleosome organization, DNA conformation change, chromatin assembly or disassembly, DNA replication-dependent nucleosome assembly, DNA replication-dependent nucleosome organization, chromatin silencing at rDNA, and chromatin silencing. As for KEGG analysis, the up-regulated DEGs were relevant to systemic lupus erythematosus, alcoholism, viral carcinogenesis, cell cycle, and neuroactive ligand-receptor interaction; meanwhile down-regulated DEGs were obviously relevant to DCM, HCM, calcium signaling pathway, ARVC, and vascular smooth muscle contraction. On the basis of degree of connectivity, we picked out 134 hub genes in the PPI network among these DEGs. After that, a random forest model was constructed to screen candidate hub genes; from these 134 genes and 25 genes were finally selected. Interestingly, some studies have proved that hub genes in the PPI network are often not disease genes (Zhao and Liu, 2019). To make sure that the hub genes we identified were associated with tumor progression, we preformed spearman correlation analysis, one-way ANOVA, and distance correlation analysis by regarding these genes and tumor grade as variables based on GSE13507. In addition, based on TCGA-BLCA data and GSE13507, we compared the expression levels of candidate hub genes in BC and normal bladder tissues to pick out genes high expressed in BC compared with those in normal tissues. Genes high expressed in BC might be associated with the happening of BC. By these five analyses, six hub genes (BUB1B, CCNB1, CDK1, ISG15, KIF15, and RAD54L) related to the progression and poor prognosis of BC were finally identified. This meant we tried our best to ensure that the hub genes we screened were disease genes.

In order to validate the six hub genes, we firstly performed stage and grade boxplots based on GSE13507. The results suggested that the high expressions of hub genes were associated with the malignant degree and progression of BC. Secondly, the mRNA expression levels of hub genes were all significantly higher in tumor tissues compared with those in normal tissues, which demonstrated that the six genes played important roles in the occurrence and progression of BC. Furthermore, in order to validate the prognosis value of the hub genes, we brought 6 factors and the hub genes expression values into Cox regression analysis among BC patients based on GSE13507. The results suggested that expression levels of all the six hub genes were associated with OS and CSS among patients with BC. We then established a six-gene signature and the risk scores were calculated combining the expression levels of the six genes and Cox regression coefficients. The Cox multivariate analysis showed that risk score, N stage, and M stage were relevant to OS and CSS among patients with BC. In order to verify the prognosis value of this six-gene signature we performed survival analysis and the results showed that the high-risk group was obviously associated with poor OS and CSS for patients with BC.

With the development of bioinformatics and high-throughput sequencing, studies indicated that small molecules might have a beneficial or detrimental effect against diseases (Qin et al., 2017). This made it possible to regard genes as novel therapeutic targets (Hansen et al., 2019; Song et al., 2019). So that we used CBioPortal to explore the relationship between hub genes and drugs aiming at finding new targets for anticancer drugs in this study. We found that CCNB1 and CDK1 were already the targets for anticancer drugs, which meant the remaining genes (BUB1B, ISG15, KIF15, and RAD54L) might become potent drug targets. CCNB1 showed higher expression in most tumor cells compared with normal cells (Fang et al., 2014). This caused deficiencies in MPF (maturation promoting factor) phosphorylation regulation mechanism (Zhang H. et al., 2018). In order to carry on the anti-tumor treatment, medicines inhibited the function of MPF through targeting CCNB1 to prevent cell mitosis (Egloff et al., 2006). Some recent studies also thought CCNB1 was a drug target. Freitas et al. (2016) thought hierridin B was a potential anticancer compound that targeted CCNB1. A study in breast cancer confirmed that targeting CCNB1 was useful in BRCA1-associated breast cancer therapy (Choi et al., 2018). In clinical, Resveratrol (Joe et al., 2002), quercetin (Choi et al., 2001), and genistein (Wiseman et al., 2007) were the representative targeted drugs. As for CDK1, it was a co-chaperone of CCNB1 (Wang et al., 2014). CDK1 could form complexes with cyclin B1 (Wang et al., 2014). CDKl/cyclin Bl complexes not only played an important role in cell division, but also increased the activity of strong mitochondria (Wang et al., 2014). According to the results of recent research, targeting CDK1 could overcome apoptotic resistance in patients with colorectal cancer (Zhang P. et al., 2018). In clinical there existed many targeted drugs for CDK1, most of which belonged to CDK inhibitors. The famous drugs were flavopiridol (Prithviraj et al., 2013) and palbociclib (Todd et al., 2015). Compared with the first-generation inhibitors, palbociclib had better selectivity, higher therapeutic index, and less side effects (Mariaule and Belmont, 2014). This suggested that improving the selectivity of CDK inhibitors was the key to improving the treatment index. For a deeper and better understanding of the remaining four genes, a literature review was carried out. BUB1B, also known as BUBR1, was an important functional protein of mitotic detection point (Pinto et al., 2014). The changes of BUB1B expression played an important role in tumorigenesis and progression (Myrie et al., 2000). ISG15 was up-regulated in the uterus, corpus luteum and liver during early pregnancy in animals as reported (Zhang L. et al., 2018). KIF15, a member of kinesin superfamily protein, could promote cell mitotic and participate in cellular material transportation (Woehlke and Schliwa, 2000). RAD54L, encoded by gene RAD54L, was shown to play an important role in homologous recombination related repair or DNA double-strand breaks (Rencic et al., 1996). In summary, we found that almost all the six hub genes were associated with cell cycle and mitosis.

Some limitations of this study also should be discussed. Firstly, lack of experiments (*in vivo* and *in vitro* validation) might be one limitation of our study. Secondly, according to the results, the six hub genes were all up-regulated in BC, but the mechanism of up-regulation was not clear. There might need more evidences to find out the biological basis. Therefore, further molecular biological experiments are needed to confirm the function of these hub genes and how they perform their roles in the progression of BC.

## Conclusion

In conclusion, by using a series of bioinformatics and retrospective analyses, the present study identified six hub genes (BUB1B, CCNB1, CDK1, ISG15, KIF15, and RAD54L), which were significantly associated with progression and prognosis of BC. These hub genes were all up-regulated in BC and four of them might be novel drug targets. Further and more in-depth study is necessary to confirm the results of the study. In any case, our study could provide some strong basis for gene targeting therapy of BC in the future and the six hub genes might be potential and novel target genes for BC.

## Author Contributions

SL, X-PL, and XY reviewed the relevant literature and drafted the manuscript. XY, X-PL, Z-XG, and T-ZL conducted all the statistical analyses. All authors read and approved the final manuscript.

## Conflict of Interest Statement

The authors declare that the research was conducted in the absence of any commercial or financial relationships that could be construed as a potential conflict of interest.

## Footnotes

1 https://genome-cancer.ucsc.edu/

2 https://www.ncbi.nlm.nih.gov/geo/

3 http://www.oncomine.org/

4 http://www.cbioportal.org/

## Supplementary Material

FIGURE S1 Volcano plot visualizing DEGs in TCGA-BC data.

FIGURE S2 The whole Protein–protein interaction (PPI) network of DEGs. Red nodes: Up-regulated DEGs. Green nodes: Down-regulated DEGs.

FIGURE S3 A random forest plot: genes with MeanDecreaseAccuracy ranked top 30 and genes with MeanDecreaseGini ranked top 30.

FIGURE S4 Expression levels of hub genes in BC and normal tissues based on GSE13507 **(A)** and GEPIA **(B)**.

FIGURE S5 Validation of the six hub genes. **(A)** Grade plot of the hub genes using GSE13507. **(B)** Stage plot of the hub genes using GSE13507. **(C)** mRNA expression levels of hub genes suggested by Oncomine database.

TABLE S1 Differentially expressed genes (DEGs) between BC samples and normal bladder tissues.

TABLE S2 Common genes in MeanDecreaseAccuracy and MeanDecreaseGini ranked top 50.

TABLE S3 One-way ANOVA analysis, spearman correlation analysis, distance correlation analysis, and AUC of candidate hub genes.
