# Supplementary figures and images for: Identification of Hub Genes Associated With Progression and Prognosis in Patients With Bladder Cancer
Source: Front Genet. 2019 May 7;10:408. doi: 10.3389/fgene.2019.00408 (PMC6513982; doi:10.3389/fgene.2019.00408)

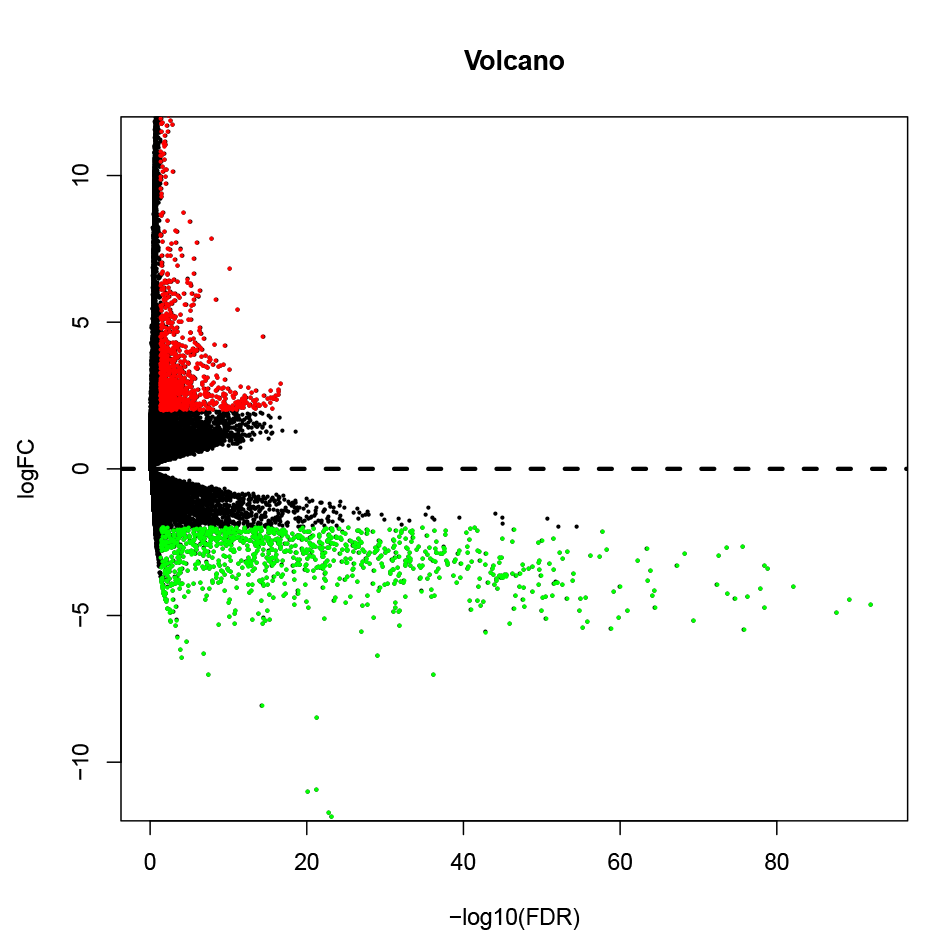

Supplement: FIGURE S1 — Volcano plot visualizing DEGs in TCGA-BC data. [file Image_1.TIF]

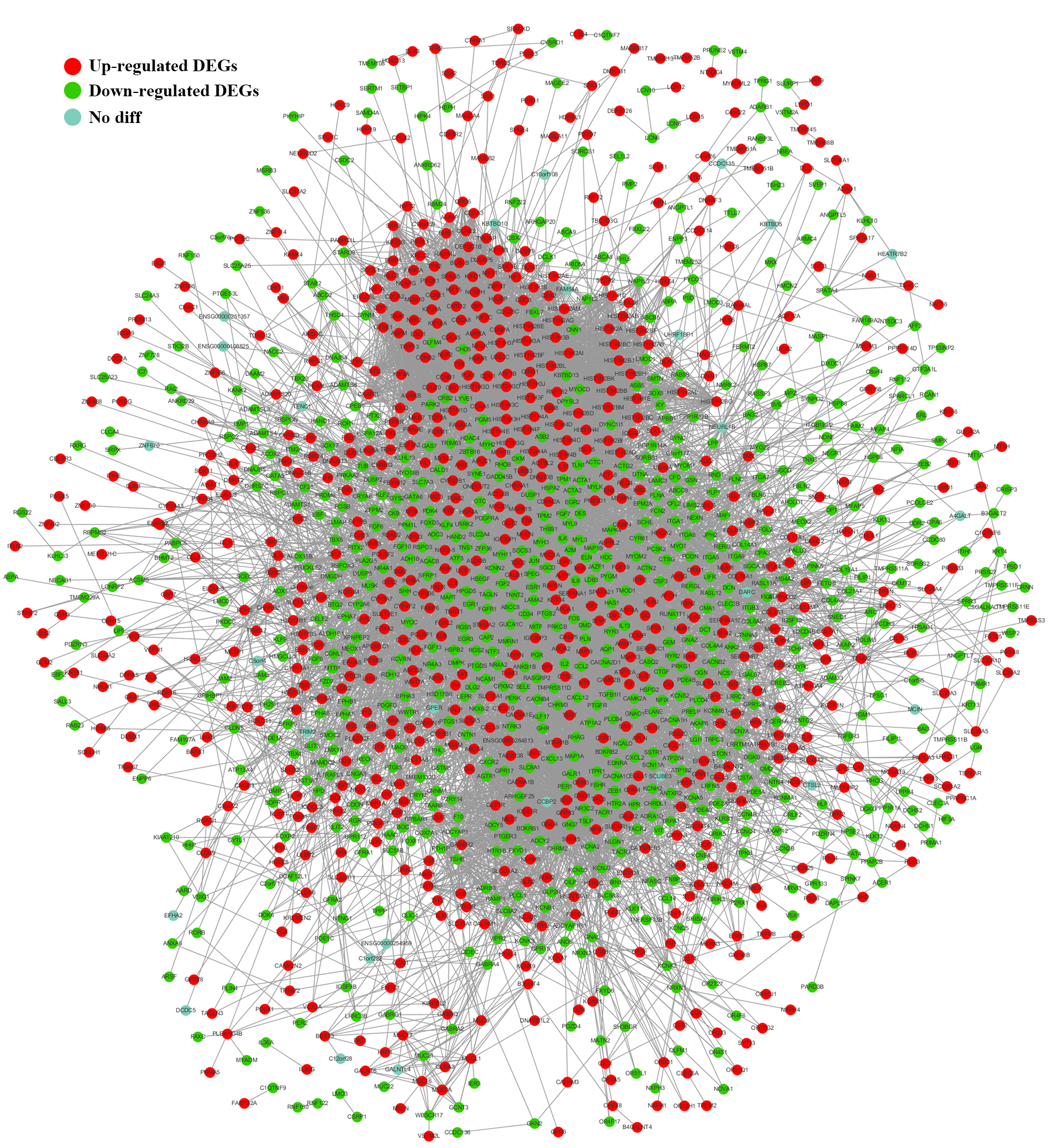

Supplement: FIGURE S2 — The whole Protein–protein interaction (PPI) network of DEGs. Red nodes: Up-regulated DEGs. Green nodes: Down-regulated DEGs. [file Image_2.TIF]

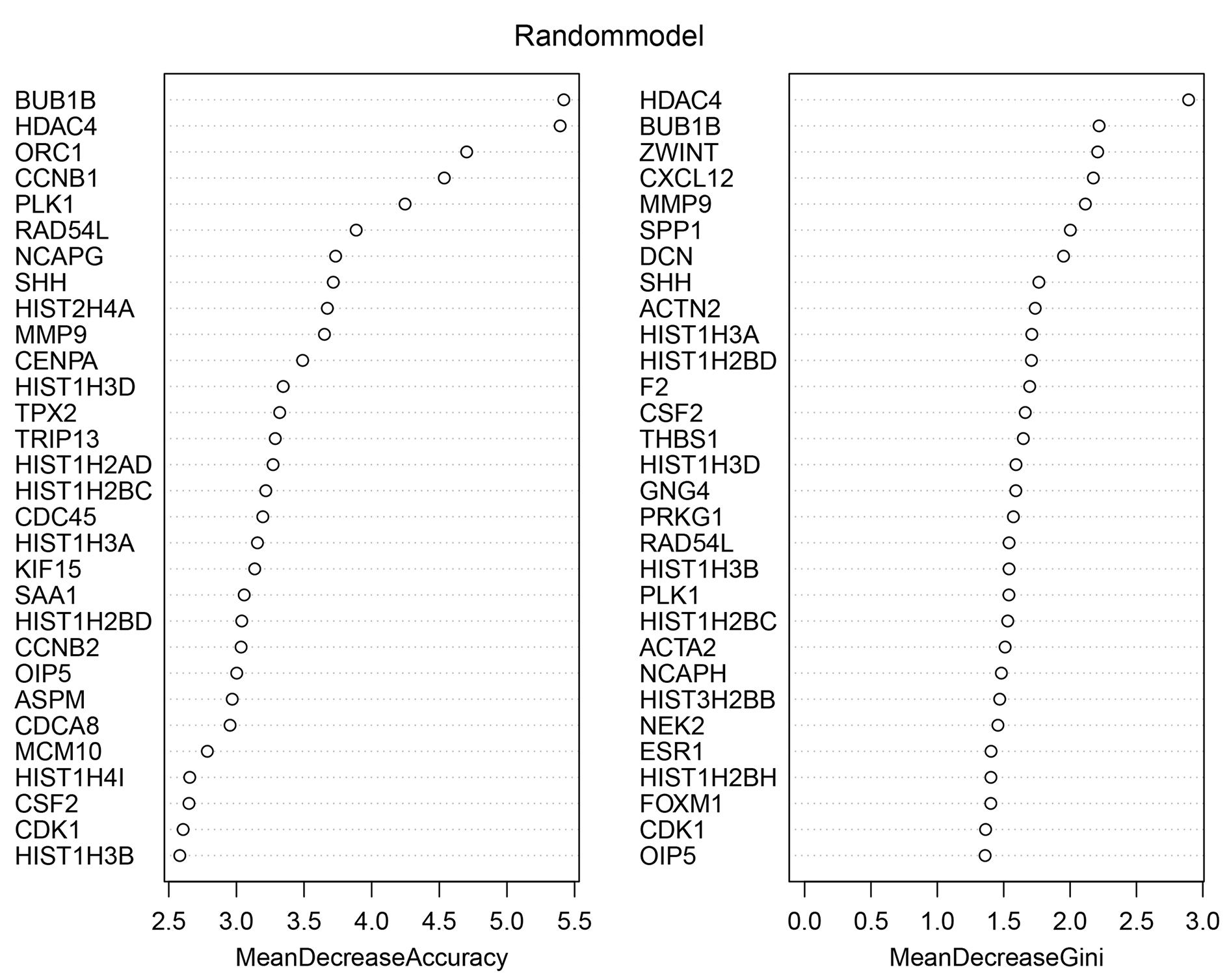

Supplement: FIGURE S3 — A random forest plot: genes with MeanDecreaseAccuracy ranked top 30 and genes with MeanDecreaseGini ranked top 30. [file Image_3.TIF]

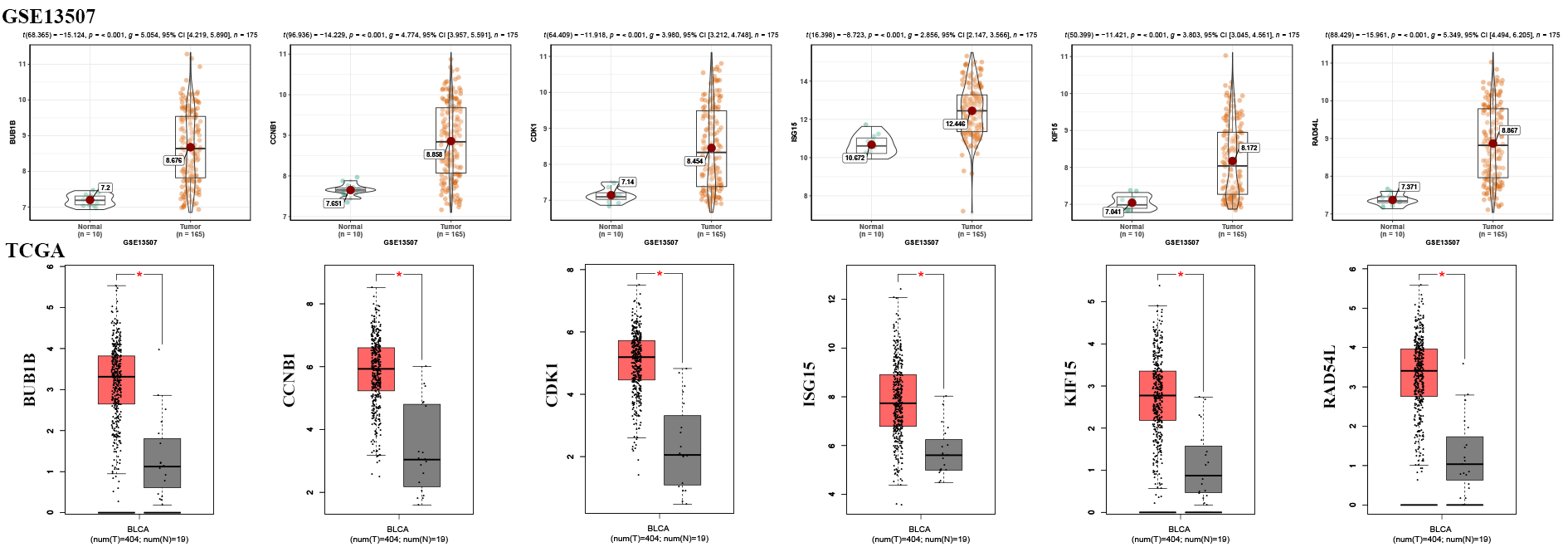

Supplement: FIGURE S4 — Expression levels of hub genes in BC and normal tissues based on GSE13507 (A) and GEPIA (B). [file Image_4.TIF]

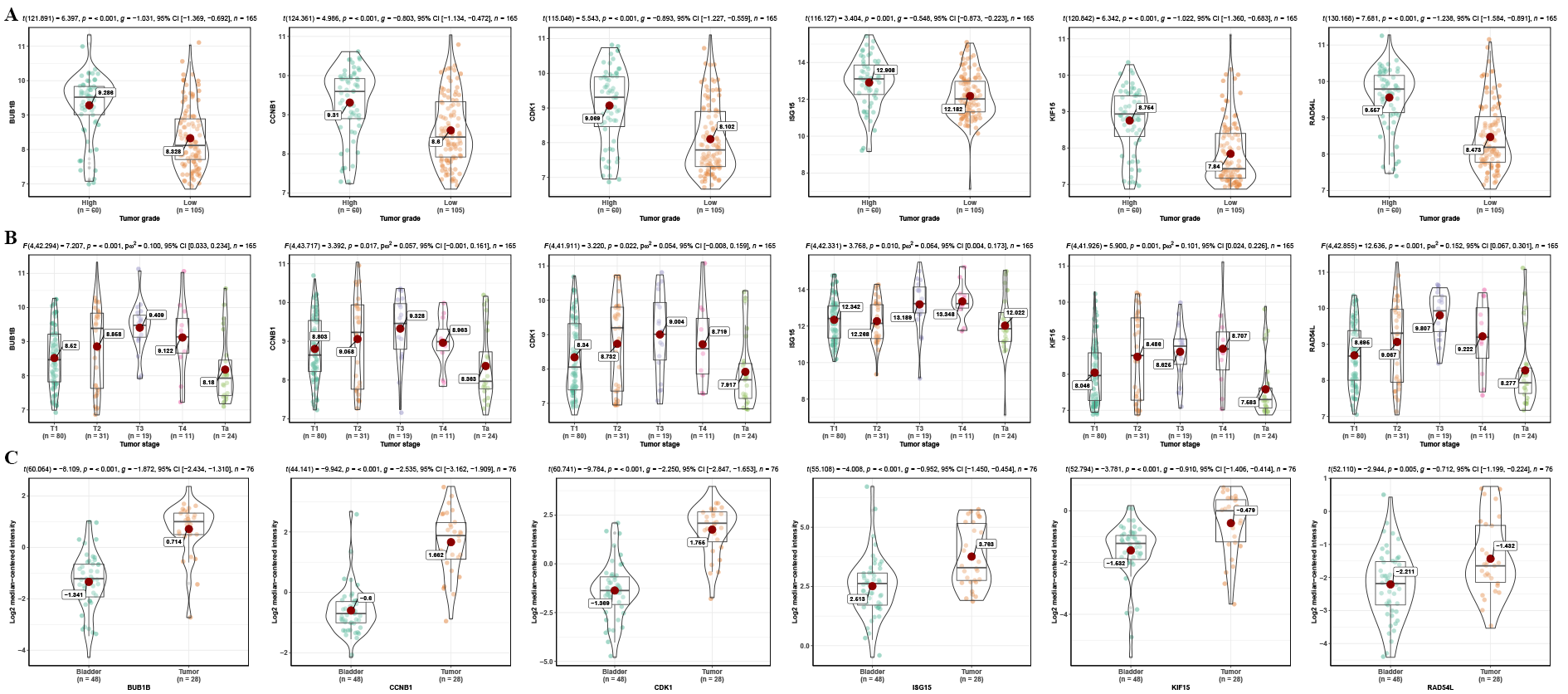

Supplement: FIGURE S5 — Validation of the six hub genes. (A) Grade plot of the hub genes using GSE13507. (B) Stage plot of the hub genes using GSE13507. (C) mRNA expression levels of hub genes suggested by Oncomine database. [file Image_5.TIF]
