# Supplementary material for: Identification of Hub Genes Associated With Progression and Prognosis in Patients With Bladder Cancer
Source: Front Genet. 2019 May 7;10:408. doi: 10.3389/fgene.2019.00408 (PMC6513982; doi:10.3389/fgene.2019.00408)
Supplement: TABLE S1 — Differentially expressed genes (DEGs) between BC samples and normal bladder tissues. [file Table_1.DOC]

**Table S 1：Differentially expressed genes (DEGs) between BC samples and normal bladder tissues.**

| up-regulated DEGs | logFC | logCPM | PValue | FDR | down-regulated DEGs | logFC | logCPM | PValue | FDR |
| --- | --- | --- | --- | --- | --- | --- | --- | --- | --- |
| KRTAP19-1 | 17.11263 | 1.0983 | 0.007423 | 0.019752 | CRNN | -8.50152 | 6.061628 | 4.39E-24 | 2.19E-22 |
| SPANXD | 15.96873 | -0.0454 | 0.013613 | 0.033049 | TMPRSS11B | -8.07762 | 0.437918 | 9.99E-17 | 2.86E-15 |
| HIST1H4F | 14.92595 | -1.08781 | 0.004597 | 0.013085 | GUCA1C | -6.98371 | -1.22132 | 1.28E-09 | 1.56E-08 |
| APCS | 14.60561 | -1.40797 | 0.007476 | 0.019872 | AC079355.1 | -6.46209 | -9.99814 | 4.46E-07 | 3.48E-06 |
| CT45A1 | 14.42625 | -1.5872 | 0.011125 | 0.027886 | MUC21 | -6.35773 | 3.562783 | 7.80E-32 | 6.39E-30 |
| LCE1A | 14.34878 | -1.66463 | 0.011287 | 0.02825 | RD3L | -5.88885 | -4.2919 | 1.41E-06 | 9.87E-06 |
| OTX2 | 13.10754 | -2.90441 | 0.002473 | 0.007644 | PI16 | -5.54365 | 4.42922 | 1.01E-45 | 1.91E-43 |
| MYOD1 | 13.07584 | -2.93602 | 0.002527 | 0.007784 | MYOC | -5.52718 | 1.270146 | 2.89E-29 | 2.05E-27 |
| APOBEC1 | 13.04403 | -2.96776 | 0.011739 | 0.029182 | SYNM | -5.43901 | 5.898858 | 9.06E-79 | 1.97E-75 |
| R3HDML | 12.98291 | -3.02878 | 0.011558 | 0.028799 | GLP2R | -5.41185 | 0.16359 | 4.56E-61 | 3.08E-58 |
| METTL21C | 12.70145 | -3.30962 | 4.81E-06 | 3.01E-05 | MYH11 | -5.36748 | 8.00624 | 7.29E-58 | 3.85E-55 |
| TEX37 | 12.50832 | -3.50227 | 0.003465 | 0.01026 | OR4F6 | -5.34194 | -9.87842 | 5.11E-05 | 0.000253 |
| PSG8 | 12.43708 | -3.5733 | 0.000744 | 0.00268 | ACTC1 | -5.301 | 5.87509 | 5.24E-34 | 4.86E-32 |
| OTOS | 12.29657 | -3.71339 | 0.001568 | 0.005139 | GPR112 | -5.26501 | -6.32209 | 3.71E-13 | 7.23E-12 |
| DPPA5 | 12.23028 | -3.77947 | 0.016417 | 0.038783 | RBFOX3 | -5.24363 | 0.704685 | 1.60E-48 | 3.90E-46 |
| FKSG70 | 12.15234 | -3.85721 | 0.009161 | 0.023696 | MYH2 | -5.23347 | -3.1529 | 2.19E-16 | 6.08E-15 |
| AQP12A | 12.04273 | -3.9664 | 0.000952 | 0.003327 | SCARA5 | -5.19627 | 2.687564 | 1.32E-58 | 7.84E-56 |
| TLX1NB | 11.86783 | -4.14059 | 0.000501 | 0.00189 | LRRC3B | -5.17018 | -1.80043 | 3.81E-17 | 1.13E-15 |
| ENSG00000268975 | 11.75555 | -4.25241 | 0.000655 | 0.002396 | SYNPO2 | -5.13838 | 5.201701 | 2.90E-72 | 3.55E-69 |
| GPR6 | 11.75272 | -4.25515 | 0.010352 | 0.026256 | TMEM252 | -5.11431 | 0.516744 | 2.12E-17 | 6.43E-16 |
| PSG3 | 11.7305 | -4.27731 | 0.000245 | 0.00101 | FAM180B | -5.08414 | -1.78528 | 2.83E-24 | 1.45E-22 |
| OR2J3 | 11.68573 | -4.32186 | 0.001535 | 0.005048 | ASB5 | -5.06056 | 1.566366 | 1.37E-16 | 3.89E-15 |
| PRSS56 | 11.65848 | 3.52052 | 0.013502 | 0.032815 | FLNC | -5.03975 | 5.464499 | 2.48E-62 | 1.80E-59 |
| PTH2 | 11.49124 | -4.51538 | 0.011594 | 0.028871 | OSTN | -5.01532 | -1.50734 | 5.03E-12 | 8.56E-11 |
| KCNV1 | 11.48526 | -4.52128 | 0.001051 | 0.003623 | MUC22 | -4.93291 | -1.29401 | 5.85E-13 | 1.12E-11 |
| AP000350.10 | 11.40177 | -4.60437 | 0.000148 | 0.000648 | MORN5 | -4.89025 | -0.26477 | 7.35E-16 | 1.94E-14 |
| SSX2 | 11.24239 | -4.76273 | 0.019025 | 0.043993 | OR51L1 | -4.88564 | -10.027 | 0.000319 | 0.001272 |
| CTB-60B18.6 | 11.06074 | -4.94314 | 0.015646 | 0.037234 | HPR | -4.87412 | -2.03666 | 2.65E-13 | 5.31E-12 |
| KRTAP9-1 | 11.05165 | -4.95217 | 0.002927 | 0.008849 | PDZRN4 | -4.85339 | 1.120135 | 2.48E-34 | 2.37E-32 |
| OR51Q1 | 10.99806 | -5.00533 | 0.004135 | 0.011944 | KLHL41 | -4.85304 | 0.442212 | 3.64E-90 | 2.37E-86 |
| ADAD1 | 10.7789 | -5.2227 | 0.019635 | 0.045154 | KLK13 | -4.83148 | 3.460787 | 2.90E-17 | 8.71E-16 |
| GC | 10.76615 | 2.098653 | 0.017733 | 0.041382 | MYOCD | -4.81363 | 1.784527 | 2.67E-57 | 1.34E-54 |
| SSX1 | 10.73598 | 1.725709 | 0.005502 | 0.015272 | CASQ2 | -4.81341 | 3.818218 | 2.43E-34 | 2.33E-32 |
| OR8D1 | 10.67845 | -5.32219 | 0.010213 | 0.025936 | PGM5 | -4.80903 | 4.13595 | 2.48E-52 | 7.96E-50 |
| OR51J1 | 10.5297 | -5.46946 | 0.016207 | 0.038348 | SLC2A4 | -4.79776 | 2.005366 | 9.56E-64 | 7.20E-61 |
| DMRTB1 | 10.49538 | -5.50342 | 0.012649 | 0.031094 | KCNB1 | -4.7675 | -1.3305 | 3.22E-43 | 5.16E-41 |
| KRT38 | 10.32187 | 1.072174 | 0.00962 | 0.024687 | HSPB6 | -4.74058 | 6.229893 | 1.25E-48 | 3.09E-46 |
| OR13H1 | 10.29572 | -5.70072 | 0.021751 | 0.04926 | KRT4 | -4.73053 | 8.01779 | 1.75E-22 | 8.00E-21 |
| PRODH2 | 10.2076 | -5.78775 | 0.001768 | 0.005713 | ATP1A2 | -4.73001 | 2.456566 | 1.64E-34 | 1.60E-32 |
| TBC1D3G | 10.13478 | -5.85964 | 0.00022 | 0.000917 | HLF | -4.71447 | 0.77404 | 8.73E-68 | 8.99E-65 |
| C20orf173 | 9.960637 | -6.03119 | 0.002485 | 0.007678 | PYGM | -4.69979 | 1.430024 | 1.69E-80 | 4.73E-77 |
| POTEG | 9.959076 | -6.0327 | 0.012837 | 0.031478 | RANBP3L | -4.66301 | 0.600391 | 5.55E-50 | 1.49E-47 |
| OR2A25 | 9.871974 | -6.1185 | 0.020209 | 0.046262 | FGF6 | -4.64554 | -5.86829 | 7.67E-05 | 0.000363 |
| OR10G2 | 9.731652 | -6.25629 | 0.01885 | 0.043661 | CNN1 | -4.63423 | 7.757547 | 2.35E-44 | 4.07E-42 |
| GCM2 | 9.571924 | -6.41296 | 0.013175 | 0.032164 | C2orf40 | -4.63306 | 2.27005 | 1.53E-29 | 1.12E-27 |
| ENSG00000264813 | 9.475223 | -6.50767 | 0.012481 | 0.030739 | VIT | -4.61395 | 0.666547 | 2.30E-20 | 9.05E-19 |
| AWAT1 | 9.368503 | -6.612 | 0.012019 | 0.029806 | OGN | -4.59725 | 2.795269 | 2.11E-30 | 1.60E-28 |
| FRG2B | 9.345977 | -6.63409 | 0.019045 | 0.04402 | MYOM1 | -4.58765 | 1.571697 | 5.50E-94 | 1.08E-89 |
| POTEC | 9.20138 | -6.77516 | 0.019807 | 0.045479 | ADH1B | -4.5469 | 3.01834 | 1.55E-27 | 9.85E-26 |
| ERVV-2 | 8.745847 | 0.608707 | 7.28E-06 | 4.37E-05 | ACTN2 | -4.5278 | 0.137919 | 1.80E-33 | 1.60E-31 |
| LIN28A | 8.522697 | -0.29489 | 0.00167 | 0.005432 | TACR3 | -4.52401 | -1.75948 | 1.60E-16 | 4.53E-15 |
| CST4 | 8.403416 | 3.063874 | 1.12E-06 | 8.05E-06 | HAND1 | -4.51837 | 0.774569 | 6.69E-12 | 1.12E-10 |
| CGB5 | 8.131025 | 3.419219 | 0.000106 | 0.000484 | PRUNE2 | -4.4938 | 2.438644 | 1.07E-44 | 1.88E-42 |
| PSG5 | 8.074422 | 0.58285 | 6.32E-05 | 0.000306 | OR2T27 | -4.48847 | -9.07395 | 0.001307 | 0.004393 |
| MUC17 | 7.938344 | -0.25741 | 0.015502 | 0.036947 | TBX20 | -4.48748 | 0.575322 | 5.25E-22 | 2.31E-20 |
| CHRND | 7.850057 | -1.66634 | 0.015818 | 0.037583 | SPINK5 | -4.47561 | 4.865411 | 1.98E-43 | 3.24E-41 |
| CST1 | 7.815487 | 6.857136 | 8.82E-10 | 1.11E-08 | KLK12 | -4.47043 | 1.982186 | 7.35E-09 | 7.99E-08 |
| ALPP | 7.73778 | 4.088692 | 1.17E-07 | 1.02E-06 | P2RX1 | -4.45646 | 3.140349 | 6.23E-44 | 1.05E-41 |
| PRSS1 | 7.678397 | 4.312884 | 0.000459 | 0.001752 | CLEC3B | -4.45213 | 3.995587 | 3.31E-91 | 3.23E-87 |
| FABP7 | 7.676821 | 3.95249 | 0.008934 | 0.023206 | DES | -4.44936 | 9.973406 | 8.55E-26 | 4.91E-24 |
| CGB8 | 7.503006 | 1.778536 | 0.001512 | 0.004982 | VSTM2A | -4.44248 | -1.9944 | 2.95E-12 | 5.18E-11 |
| APOA2 | 7.298138 | 5.95322 | 0.001809 | 0.005833 | LMOD1 | -4.43435 | 6.060991 | 4.24E-50 | 1.15E-47 |
| CGB | 7.278913 | 1.104109 | 0.000589 | 0.002181 | TACR2 | -4.40013 | 2.315022 | 1.14E-57 | 5.89E-55 |
| ALB | 7.277768 | 3.182157 | 1.43E-05 | 8.07E-05 | TMEM132C | -4.38809 | -0.87627 | 6.45E-18 | 2.07E-16 |
| KRT81 | 7.187528 | 6.031455 | 2.94E-07 | 2.37E-06 | SORCS1 | -4.38699 | -1.79186 | 2.13E-26 | 1.27E-24 |
| PAEP | 7.105477 | 1.426443 | 0.000146 | 0.000642 | FHL1 | -4.38558 | 5.348456 | 5.82E-56 | 2.59E-53 |
| AHSG | 7.031752 | -0.60023 | 0.011054 | 0.027742 | FILIP1 | -4.37677 | 1.604316 | 3.02E-76 | 5.37E-73 |
| HAVCR1 | 6.935391 | 2.692703 | 5.45E-05 | 0.000268 | SORBS1 | -4.36256 | 4.519248 | 3.29E-58 | 1.79E-55 |
| FGF19 | 6.916719 | 1.995047 | 0.011725 | 0.029153 | CHRM2 | -4.35482 | 0.589014 | 1.51E-20 | 6.02E-19 |
| COL10A1 | 6.834767 | 4.325347 | 3.16E-12 | 5.53E-11 | KIAA1644 | -4.3497 | 1.557444 | 8.36E-53 | 2.87E-50 |
| IGFL2 | 6.656028 | 3.063808 | 2.83E-07 | 2.29E-06 | MMP27 | -4.34954 | -4.56211 | 4.35E-06 | 2.75E-05 |
| MYBPH | 6.64876 | 2.468712 | 0.00189 | 0.006053 | OR4F17 | -4.34451 | -10.0771 | 0.001572 | 0.005149 |
| KRT85 | 6.627838 | -0.69238 | 0.011037 | 0.027715 | PLP1 | -4.32682 | -0.192 | 4.86E-28 | 3.18E-26 |
| FGL1 | 6.623061 | -2.10393 | 0.001833 | 0.005898 | ACTG2 | -4.32616 | 8.448872 | 6.11E-38 | 7.38E-36 |
| ALPPL2 | 6.469011 | 1.106799 | 0.000185 | 0.000786 | SCN11A | -4.32382 | 0.264209 | 1.38E-30 | 1.06E-28 |
| SLC1A6 | 6.464064 | 4.012973 | 2.36E-06 | 1.58E-05 | PTGS1 | -4.32361 | 5.250918 | 6.88E-53 | 2.40E-50 |
| ROS1 | 6.38519 | 0.230301 | 6.30E-05 | 0.000305 | C16orf89 | -4.32147 | 2.040131 | 9.69E-39 | 1.23E-36 |
| HSD3B1 | 6.369744 | 2.390983 | 0.000999 | 0.003467 | TNS1 | -4.32006 | 5.366724 | 6.06E-78 | 1.19E-74 |
| MAGEB2 | 6.351967 | 2.277284 | 0.004997 | 0.014066 | TNXB | -4.3055 | 1.993422 | 5.75E-54 | 2.21E-51 |
| KCNH6 | 6.349611 | 1.30296 | 0.000555 | 0.002072 | ADAMTSL3 | -4.28455 | 0.101239 | 2.82E-49 | 7.25E-47 |
| APOH | 6.316778 | 0.318918 | 0.014456 | 0.034789 | MYLK | -4.2739 | 4.77778 | 5.67E-66 | 5.05E-63 |
| COL2A1 | 6.304477 | 1.004163 | 2.35E-06 | 1.57E-05 | TAAR6 | -4.26444 | -7.60377 | 0.002138 | 0.006733 |
| IL37 | 6.279353 | 1.01863 | 6.73E-07 | 5.05E-06 | TCF21 | -4.2469 | 2.018917 | 2.11E-75 | 3.17E-72 |
| ESX1 | 6.274093 | -1.0899 | 0.014804 | 0.035495 | PCP4 | -4.24383 | 5.858031 | 4.18E-17 | 1.24E-15 |
| NLRP7 | 6.271616 | 3.794198 | 9.83E-07 | 7.13E-06 | FAM135B | -4.24087 | -4.27557 | 5.18E-15 | 1.24E-13 |
| KLK2 | 6.165763 | 0.536389 | 0.001128 | 0.003857 | ANGPTL7 | -4.2331 | -0.5033 | 2.78E-19 | 1.02E-17 |
| NAA11 | 6.133939 | 0.546067 | 0.012806 | 0.031425 | COL19A1 | -4.21526 | -1.39023 | 4.33E-21 | 1.79E-19 |
| PIWIL3 | 6.121098 | -1.15606 | 0.005503 | 0.015272 | NEGR1 | -4.16826 | 1.043096 | 2.65E-51 | 7.75E-49 |
| CST2 | 6.110661 | 2.498545 | 3.13E-08 | 3.03E-07 | GPR133 | -4.14122 | 0.616981 | 7.69E-37 | 8.75E-35 |
| PNMA5 | 6.034265 | 3.625068 | 0.000695 | 0.002523 | KCNMA1 | -4.13636 | 1.360141 | 1.61E-60 | 1.02E-57 |
| HMGA2 | 5.969033 | 2.011791 | 4.90E-07 | 3.81E-06 | KIAA0408 | -4.13313 | -4.89032 | 1.53E-11 | 2.45E-10 |
| KRT79 | 5.961687 | 3.1492 | 8.14E-06 | 4.83E-05 | PMP2 | -4.12035 | -1.95737 | 7.83E-15 | 1.83E-13 |
| GUCA2A | 5.955366 | 4.547262 | 0.001868 | 0.005991 | AARD | -4.10362 | 0.72429 | 5.27E-21 | 2.15E-19 |
| VIL1 | 5.955071 | 0.068338 | 0.000151 | 0.000658 | POPDC2 | -4.09809 | 3.43067 | 1.62E-65 | 1.27E-62 |
| MS4A15 | 5.951188 | 1.947011 | 0.00031 | 0.00124 | PLN | -4.09686 | 4.487101 | 4.62E-31 | 3.63E-29 |
| SERPINA6 | 5.921001 | 0.389494 | 0.00835 | 0.021882 | TMPRSS11A | -4.05777 | 2.093562 | 3.57E-13 | 7.00E-12 |
| SPDYC | 5.906811 | 1.638707 | 5.15E-05 | 0.000254 | BHMT2 | -4.04289 | 0.97587 | 1.13E-46 | 2.37E-44 |
| 4-Mar | 5.88959 | 0.480868 | 5.66E-08 | 5.25E-07 | C7 | -4.01332 | 4.101123 | 2.53E-24 | 1.30E-22 |
| TNNI3 | 5.879278 | 1.642907 | 1.38E-07 | 1.18E-06 | FAM107A | -4.00565 | 2.359533 | 8.39E-50 | 2.22E-47 |
| TMEM82 | 5.846294 | -2.37999 | 0.001029 | 0.003556 | CPED1 | -3.99887 | 1.992797 | 7.41E-62 | 5.18E-59 |
| GBX1 | 5.816471 | -0.76222 | 0.001834 | 0.005901 | CABS1 | -3.98072 | -6.7387 | 0.001507 | 0.004971 |
| NKX2-5 | 5.807412 | 0.122063 | 0.004005 | 0.011617 | DTNA | -3.96968 | 1.492915 | 3.46E-44 | 5.89E-42 |
| BARX1 | 5.766021 | 3.267283 | 2.25E-07 | 1.86E-06 | PPP1R12B | -3.96817 | 3.876708 | 2.93E-86 | 1.44E-82 |
| COL11A1 | 5.760636 | 3.822084 | 2.87E-10 | 3.85E-09 | OR4S1 | -3.96244 | -9.15793 | 0.005624 | 0.015565 |
| CLDN6 | 5.753646 | 2.768837 | 0.000182 | 0.000778 | DYNAP | -3.96064 | -3.57235 | 0.000268 | 0.00109 |
| FOXA2 | 5.747931 | 0.55476 | 0.006408 | 0.017387 | SERPINB12 | -3.94861 | 0.096118 | 1.04E-05 | 6.07E-05 |
| PRAME | 5.744619 | 3.628671 | 0.00014 | 0.000617 | FGF10 | -3.94536 | -0.23165 | 3.98E-15 | 9.61E-14 |
| SOHLH1 | 5.734067 | 0.242692 | 0.004553 | 0.012967 | CNGA3 | -3.9433 | -2.85599 | 1.70E-16 | 4.76E-15 |
| PRDM13 | 5.726197 | -2.14236 | 0.005004 | 0.014079 | SERTM1 | -3.94325 | -2.9025 | 2.14E-06 | 1.44E-05 |
| TRIM71 | 5.659195 | -1.40484 | 0.000443 | 0.001701 | NTRK3 | -3.93162 | -2.44809 | 3.14E-31 | 2.51E-29 |
| HIST1H1B | 5.642546 | 2.896132 | 3.15E-06 | 2.05E-05 | SGCG | -3.92771 | -0.30781 | 1.63E-14 | 3.69E-13 |
| EPYC | 5.634125 | 1.478103 | 0.000367 | 0.001437 | DGKB | -3.92525 | -1.88529 | 4.59E-17 | 1.35E-15 |
| HIST1H3B | 5.60531 | 3.207472 | 4.80E-07 | 3.74E-06 | RHCG | -3.91694 | 6.869863 | 1.32E-19 | 4.97E-18 |
| MUC16 | 5.596237 | 1.104132 | 5.48E-06 | 3.38E-05 | C1orf95 | -3.91162 | 0.643667 | 6.14E-26 | 3.56E-24 |
| CPLX2 | 5.59334 | 0.62515 | 0.001189 | 0.004039 | KCNMB1 | -3.90684 | 2.89203 | 6.04E-46 | 1.16E-43 |
| PAGE2B | 5.592295 | 2.695443 | 0.008819 | 0.022955 | LMOD3 | -3.90156 | -1.75402 | 5.43E-75 | 7.59E-72 |
| C4orf26 | 5.573202 | 1.24879 | 3.12E-07 | 2.50E-06 | FSHR | -3.89975 | -6.56915 | 0.000425 | 0.001638 |
| BEST3 | 5.569283 | -1.45436 | 0.001315 | 0.004418 | AKAP6 | -3.89804 | -0.25112 | 2.04E-54 | 8.16E-52 |
| HMX2 | 5.543549 | -1.31195 | 0.001704 | 0.005526 | DMD | -3.8972 | 1.552228 | 2.14E-51 | 6.34E-49 |
| LCN15 | 5.542981 | 0.41579 | 0.016293 | 0.038522 | DHRS7C | -3.8902 | -4.96569 | 0.000387 | 0.001509 |
| BAAT | 5.475001 | 0.264057 | 0.000689 | 0.002505 | LRRC2 | -3.88644 | -1.00149 | 1.73E-32 | 1.45E-30 |
| CYP2B6 | 5.45923 | 0.679117 | 0.013482 | 0.032777 | MYL9 | -3.88616 | 9.141713 | 8.78E-53 | 2.92E-50 |
| MMP11 | 5.423538 | 6.749308 | 2.64E-13 | 5.29E-12 | RHAG | -3.88429 | -4.92667 | 0.000116 | 0.000524 |
| IBSP | 5.398899 | 2.646572 | 6.79E-07 | 5.08E-06 | PSD | -3.85973 | 3.02728 | 2.06E-53 | 7.46E-51 |
| PPY | 5.338375 | 0.87406 | 0.014035 | 0.033922 | REEP1 | -3.85863 | 1.721139 | 4.93E-33 | 4.28E-31 |
| PSG4 | 5.335412 | 0.837242 | 0.000557 | 0.002079 | NR4A3 | -3.85296 | 3.151426 | 2.11E-45 | 3.89E-43 |
| ENSG00000274997 | 5.314616 | 1.346132 | 2.04E-05 | 0.000111 | SPINK7 | -3.85094 | 1.679879 | 7.96E-10 | 1.01E-08 |
| HIST1H2BM | 5.25509 | -0.14694 | 0.000271 | 0.001101 | CYP2A6 | -3.85034 | -0.07555 | 5.99E-11 | 8.87E-10 |
| DGKK | 5.235905 | -1.85096 | 0.009856 | 0.025203 | AFF3 | -3.84072 | 0.037127 | 1.05E-34 | 1.07E-32 |
| ITLN1 | 5.23263 | 6.316293 | 0.001012 | 0.003506 | SDPR | -3.8349 | 4.145398 | 1.89E-53 | 6.99E-51 |
| KRTAP2-3 | 5.217339 | 0.279021 | 0.012855 | 0.031508 | GATA5 | -3.82413 | 1.257136 | 4.86E-16 | 1.31E-14 |
| NXPH1 | 5.205756 | -2.44539 | 0.00271 | 0.008274 | PCOLCE2 | -3.8189 | 2.700712 | 3.51E-34 | 3.29E-32 |
| SLC22A11 | 5.185756 | -0.43948 | 0.000444 | 0.001704 | HSPB7 | -3.81376 | 3.728004 | 2.11E-28 | 1.42E-26 |
| C1orf105 | 5.166915 | -1.34701 | 0.002212 | 0.006933 | DPCR1 | -3.80534 | -1.7478 | 8.15E-10 | 1.03E-08 |
| PRR9 | 5.158294 | 4.299639 | 0.003201 | 0.009575 | CLEC3A | -3.80524 | 1.072849 | 6.36E-08 | 5.84E-07 |
| TTR | 5.09831 | 4.607884 | 0.012127 | 0.030033 | C1orf177 | -3.80415 | -0.349 | 6.64E-25 | 3.56E-23 |
| B4GALNT2 | 5.091092 | -1.63057 | 0.001892 | 0.006055 | NR4A1 | -3.79952 | 5.875979 | 3.32E-56 | 1.51E-53 |
| PPP1R14D | 5.084531 | 3.907791 | 6.18E-07 | 4.68E-06 | LMO3 | -3.79293 | 1.766827 | 1.34E-34 | 1.35E-32 |
| PSORS1C2 | 5.059376 | 5.978314 | 1.44E-06 | 1.01E-05 | PDE1C | -3.79185 | -0.34033 | 4.93E-46 | 9.55E-44 |
| FGF8 | 5.045533 | 1.324301 | 0.001166 | 0.003972 | CMTM5 | -3.78711 | -2.54589 | 5.86E-18 | 1.89E-16 |
| GFY | 5.045386 | -0.2151 | 0.001192 | 0.004048 | SCN7A | -3.77665 | -0.48604 | 6.47E-16 | 1.72E-14 |
| HIST1H4B | 5.030493 | 1.740226 | 0.000302 | 0.001209 | FAM129A | -3.77255 | 4.856405 | 7.73E-66 | 6.57E-63 |
| KRT14 | 5.01395 | 10.53658 | 4.08E-05 | 0.000206 | FOSB | -3.76134 | 6.800297 | 1.94E-38 | 2.44E-36 |
| ARX | 4.97673 | -1.40586 | 0.000624 | 0.002295 | ADRB3 | -3.75629 | -0.29584 | 2.09E-16 | 5.83E-15 |
| A1CF | 4.962542 | -1.94701 | 0.001369 | 0.004575 | HPSE2 | -3.75544 | 2.003115 | 1.56E-20 | 6.21E-19 |
| HABP2 | 4.956287 | -0.28807 | 0.001116 | 0.003821 | ZBTB16 | -3.73566 | 0.029928 | 9.91E-27 | 6.01E-25 |
| AMTN | 4.943714 | 2.387188 | 0.009412 | 0.024237 | C1QTNF7 | -3.73043 | 0.686212 | 1.65E-46 | 3.35E-44 |
| TRIML2 | 4.895997 | -0.24255 | 0.014893 | 0.035662 | CAMK2A | -3.71608 | -0.97484 | 2.81E-26 | 1.66E-24 |
| PIP | 4.850006 | 2.689773 | 0.008798 | 0.022904 | METTL24 | -3.71251 | -0.32927 | 3.48E-24 | 1.76E-22 |
| ZIC5 | 4.842013 | -0.11755 | 0.00047 | 0.001789 | EGR3 | -3.71139 | 3.167938 | 1.52E-47 | 3.42E-45 |
| MTNR1B | 4.840367 | -1.44117 | 0.011287 | 0.02825 | CPNE6 | -3.70991 | -2.24018 | 1.19E-12 | 2.19E-11 |
| HIST1H2BI | 4.834422 | 1.301405 | 8.00E-05 | 0.000377 | KLF17 | -3.70009 | -1.60873 | 9.33E-11 | 1.34E-09 |
| NR5A1 | 4.821296 | -0.49205 | 0.002775 | 0.008456 | PRELP | -3.69884 | 4.230124 | 1.57E-36 | 1.75E-34 |
| ORM1 | 4.796208 | 1.682264 | 0.020652 | 0.04715 | XPNPEP2 | -3.69771 | 0.875379 | 4.51E-17 | 1.33E-15 |
| SERPINA10 | 4.793964 | -2.71208 | 0.00946 | 0.024339 | CRYAB | -3.69761 | 5.081168 | 1.33E-43 | 2.21E-41 |
| CLPSL2 | 4.770821 | -0.57065 | 0.00133 | 0.004465 | INMT-FAM188B | -3.69655 | -6.29203 | 7.76E-05 | 0.000367 |
| PRSS33 | 4.75804 | 0.492709 | 0.013824 | 0.033487 | FIGF | -3.68617 | 1.118144 | 1.84E-36 | 2.05E-34 |
| HIST1H2BO | 4.745084 | 2.49882 | 4.45E-08 | 4.20E-07 | ABCB5 | -3.6806 | -3.78828 | 2.45E-10 | 3.32E-09 |
| RDH8 | 4.69509 | -2.27398 | 0.01968 | 0.045241 | RBPMS2 | -3.67994 | 4.15125 | 2.15E-46 | 4.29E-44 |
| FOLR1 | 4.669913 | 3.448225 | 9.55E-05 | 0.00044 | HMCN2 | -3.67878 | -0.15211 | 1.32E-41 | 1.94E-39 |
| MAGEA11 | 4.659225 | 1.748537 | 0.000757 | 0.00272 | DOK6 | -3.66772 | 0.196788 | 3.56E-39 | 4.58E-37 |
| IGF2BP1 | 4.632766 | 1.565449 | 7.03E-05 | 0.000337 | SPRR3 | -3.66502 | 8.009331 | 2.58E-18 | 8.60E-17 |
| CASP14 | 4.62733 | 6.884576 | 0.000233 | 0.000963 | ELANE | -3.66451 | -1.20763 | 1.91E-12 | 3.41E-11 |
| LHX5 | 4.625498 | 0.565018 | 8.37E-07 | 6.16E-06 | CFD | -3.66259 | 6.510227 | 1.24E-46 | 2.59E-44 |
| ELAVL3 | 4.605992 | -0.35416 | 0.005973 | 0.016376 | KRT78 | -3.66025 | 1.94353 | 3.23E-19 | 1.18E-17 |
| SPP1 | 4.589762 | 8.840686 | 3.60E-08 | 3.45E-07 | NCAM1 | -3.65795 | 1.937367 | 1.02E-22 | 4.71E-21 |
| CALB1 | 4.575921 | 3.360996 | 0.000219 | 0.000912 | F10 | -3.65043 | 1.530077 | 1.60E-47 | 3.51E-45 |
| LHX1 | 4.567067 | -0.16015 | 0.002212 | 0.006933 | DCAF12L1 | -3.63548 | -4.20825 | 0.000171 | 0.000733 |
| VAX1 | 4.555796 | -2.28316 | 0.018824 | 0.043622 | SGCA | -3.63267 | 2.766643 | 3.23E-27 | 2.03E-25 |
| HRG | 4.51031 | -2.369 | 0.015681 | 0.037296 | SORBS2 | -3.62394 | 2.422916 | 2.12E-46 | 4.28E-44 |
| MAGEA4 | 4.497956 | 4.763386 | 0.011041 | 0.027715 | PDK4 | -3.61555 | 4.773952 | 3.87E-41 | 5.57E-39 |
| ESM1 | 4.490398 | 4.088966 | 1.89E-16 | 5.28E-15 | FGL2 | -3.6066 | 4.366934 | 1.08E-47 | 2.48E-45 |
| HIST1H1D | 4.47364 | 2.282096 | 3.18E-07 | 2.55E-06 | XKR4 | -3.60654 | -3.87536 | 2.00E-13 | 4.08E-12 |
| NGB | 4.426041 | 0.435899 | 0.003329 | 0.009909 | IGSF10 | -3.60301 | -0.27657 | 3.69E-19 | 1.34E-17 |
| TNNT1 | 4.402716 | 4.124778 | 1.37E-08 | 1.42E-07 | LPPR4 | -3.60226 | 0.862566 | 1.23E-49 | 3.22E-47 |
| IGF2BP3 | 4.39437 | 1.325566 | 9.82E-07 | 7.12E-06 | MAMDC2 | -3.59987 | 2.43468 | 5.66E-34 | 5.23E-32 |
| CHRNA9 | 4.384357 | -0.59001 | 0.00624 | 0.017004 | AOX1 | -3.59843 | 1.43405 | 7.17E-28 | 4.66E-26 |
| SYT13 | 4.371374 | 0.145332 | 0.006296 | 0.017137 | RAB9B | -3.59431 | 1.379703 | 1.08E-51 | 3.34E-49 |
| OBP2A | 4.338969 | -1.43645 | 0.002562 | 0.007876 | RNF150 | -3.59107 | 1.155403 | 1.57E-45 | 2.93E-43 |
| HIST1H3F | 4.332886 | 1.678218 | 0.000266 | 0.001085 | NRXN1 | -3.58966 | -2.356 | 2.05E-11 | 3.19E-10 |
| GAL | 4.329946 | 3.376803 | 0.000105 | 0.000481 | KCNE4 | -3.58903 | 2.897069 | 1.09E-45 | 2.06E-43 |
| VCX3A | 4.323855 | 1.304205 | 0.013769 | 0.033374 | TMOD1 | -3.58811 | 2.025075 | 1.12E-37 | 1.32E-35 |
| HIST1H3I | 4.319907 | 0.431536 | 0.001076 | 0.003702 | MSRB3 | -3.58028 | 4.299989 | 6.15E-47 | 1.31E-44 |
| KRT39 | 4.319067 | -0.45126 | 0.00979 | 0.025065 | LMX1A | -3.58011 | -4.50677 | 4.31E-05 | 0.000216 |
| NEFL | 4.311752 | 2.414011 | 0.008086 | 0.021287 | C1QTNF9 | -3.57866 | -2.32176 | 3.63E-10 | 4.78E-09 |
| LBP | 4.292835 | 2.471267 | 7.52E-05 | 0.000357 | CACNA1H | -3.57558 | 3.347471 | 1.61E-46 | 3.32E-44 |
| OR2B6 | 4.273229 | 0.679806 | 2.38E-07 | 1.95E-06 | ITIH5 | -3.55749 | 2.75985 | 7.86E-49 | 2.00E-46 |
| GGT2 | 4.269475 | -0.25837 | 0.001534 | 0.005045 | STON1-GTF2A1L | -3.55422 | -3.00177 | 1.64E-14 | 3.71E-13 |
| ACTL6B | 4.256655 | -0.74652 | 0.016218 | 0.038361 | ENDOU | -3.55302 | 0.688105 | 2.82E-18 | 9.36E-17 |
| HIST1H3G | 4.250244 | 1.651722 | 3.74E-10 | 4.92E-09 | FETUB | -3.54288 | -2.19879 | 2.74E-05 | 0.000144 |
| PPAPDC1A | 4.229557 | 2.903 | 1.14E-07 | 9.97E-07 | IGSF9B | -3.53383 | -0.76587 | 1.26E-32 | 1.06E-30 |
| EN2 | 4.226865 | 0.170799 | 2.73E-05 | 0.000144 | JPH2 | -3.53097 | 3.193081 | 1.11E-33 | 1.00E-31 |
| HIST1H2AD | 4.224034 | 3.203243 | 1.63E-11 | 2.59E-10 | WBSCR17 | -3.52923 | 1.389797 | 3.24E-24 | 1.65E-22 |
| IGFL4 | 4.219246 | -1.63942 | 3.54E-05 | 0.000181 | RP11-766F14.2 | -3.52303 | -2.63076 | 4.61E-10 | 5.99E-09 |
| CALHM3 | 4.215973 | 2.075902 | 0.000147 | 0.000644 | FBXL22 | -3.52216 | 1.651053 | 3.09E-56 | 1.44E-53 |
| ZG16 | 4.179014 | -2.91504 | 0.012549 | 0.030883 | AOC3 | -3.51517 | 5.055845 | 1.17E-44 | 2.05E-42 |
| TMEM52B | 4.171028 | 2.016522 | 7.12E-08 | 6.48E-07 | PTGFR | -3.50381 | 1.097125 | 1.98E-24 | 1.02E-22 |
| ENTPD8 | 4.155404 | 1.62129 | 5.42E-05 | 0.000266 | RXRG | -3.50077 | -1.59922 | 2.58E-12 | 4.53E-11 |
| HIST1H4C | 4.151738 | 2.063969 | 4.13E-05 | 0.000209 | IL6 | -3.49341 | 4.565798 | 2.91E-20 | 1.13E-18 |
| PGC | 4.149714 | 0.971257 | 0.008305 | 0.021789 | RERGL | -3.48106 | 0.268895 | 1.98E-15 | 4.96E-14 |
| CLDN9 | 4.148413 | 2.955364 | 4.42E-06 | 2.79E-05 | ASB2 | -3.48028 | 2.368638 | 6.48E-38 | 7.78E-36 |
| CHRNA1 | 4.14114 | 0.187352 | 3.79E-08 | 3.62E-07 | GDF10 | -3.47698 | -1.39004 | 3.95E-11 | 5.95E-10 |
| HIST1H4E | 4.114413 | 3.680726 | 1.08E-07 | 9.50E-07 | VIPR2 | -3.46749 | -1.34579 | 3.16E-22 | 1.42E-20 |
| KRT37 | 4.109606 | -1.29614 | 0.009226 | 0.023814 | FAIM2 | -3.46079 | 0.204625 | 5.72E-23 | 2.69E-21 |
| TAS2R38 | 4.106601 | -1.44249 | 0.00198 | 0.006295 | GNAO1 | -3.46075 | 0.918746 | 1.25E-32 | 1.06E-30 |
| POU4F3 | 4.105901 | 0.59124 | 0.003524 | 0.010427 | CPEB1 | -3.45306 | -1.01942 | 9.82E-31 | 7.60E-29 |
| POU3F2 | 4.0896 | -0.53734 | 0.002352 | 0.007318 | TPM1 | -3.45071 | 6.201001 | 1.56E-67 | 1.53E-64 |
| CDKN2A | 4.081105 | 5.527707 | 3.82E-07 | 3.01E-06 | DPT | -3.44433 | 4.845441 | 1.40E-16 | 3.98E-15 |
| GCM1 | 4.078827 | -0.13313 | 0.000876 | 0.003085 | CD300LG | -3.43886 | -1.94483 | 6.33E-12 | 1.06E-10 |
| HIST1H2BF | 4.075647 | 2.605694 | 3.20E-07 | 2.56E-06 | GKN2 | -3.4348 | -3.89017 | 0.001122 | 0.003841 |
| IGFL1 | 4.067599 | 7.96512 | 0.000305 | 0.001222 | ITGA8 | -3.42522 | 2.267307 | 2.19E-50 | 6.04E-48 |
| CYP2C9 | 4.067157 | 1.128192 | 0.003801 | 0.011128 | CSF3 | -3.42167 | 4.01379 | 6.23E-12 | 1.05E-10 |
| HIST1H2AI | 4.052661 | 2.698298 | 5.80E-07 | 4.42E-06 | FXYD6 | -3.41876 | 3.567394 | 6.58E-33 | 5.69E-31 |
| SLCO1B1 | 4.050869 | -1.87392 | 0.016886 | 0.039725 | ABCA8 | -3.41807 | 0.58266 | 1.76E-28 | 1.19E-26 |
| ZNF114 | 4.044781 | 2.116559 | 2.27E-08 | 2.25E-07 | SMPX | -3.41519 | 0.791313 | 1.32E-10 | 1.86E-09 |
| HIST1H1E | 4.038805 | 3.700055 | 1.97E-07 | 1.64E-06 | ACER1 | -3.40316 | -1.72637 | 1.19E-08 | 1.25E-07 |
| HIST1H2AB | 4.037255 | 0.853616 | 0.002374 | 0.007376 | LIMS2 | -3.40189 | 3.568286 | 1.68E-51 | 5.15E-49 |
| SOX11 | 4.035456 | -1.04638 | 1.15E-07 | 1.00E-06 | PTGIS | -3.40099 | 4.807565 | 1.30E-21 | 5.60E-20 |
| MMP13 | 4.017629 | 5.712894 | 3.04E-05 | 0.000158 | EGR1 | -3.40056 | 8.148213 | 2.41E-54 | 9.43E-52 |
| TEX19 | 3.999343 | -2.75887 | 0.000699 | 0.002535 | SPATA31A7 | -3.39966 | -9.58731 | 0.011146 | 0.02793 |
| SLC35D3 | 3.998844 | -0.36357 | 0.003134 | 0.009395 | AGTR1 | -3.39489 | 0.980878 | 3.13E-19 | 1.14E-17 |
| TM4SF19 | 3.997681 | 1.622988 | 2.96E-06 | 1.94E-05 | ARSF | -3.39321 | -1.18854 | 2.25E-09 | 2.65E-08 |
| PAGE5 | 3.989546 | 2.031201 | 0.004605 | 0.013101 | SBSPON | -3.39219 | 3.230461 | 8.01E-33 | 6.88E-31 |
| LEMD1 | 3.987791 | 0.858454 | 0.000148 | 0.000649 | KLHL40 | -3.39107 | -6.46433 | 0.002197 | 0.006894 |
| RNF186 | 3.984409 | 1.699725 | 0.007948 | 0.020974 | TPPP | -3.39057 | 1.995205 | 7.06E-39 | 9.03E-37 |
| C6orf223 | 3.978867 | 1.435694 | 1.11E-06 | 7.98E-06 | HAND2 | -3.39053 | 1.979558 | 3.88E-24 | 1.95E-22 |
| MEP1A | 3.961922 | -0.19172 | 0.001109 | 0.003799 | ADAMTS1 | -3.38438 | 5.275573 | 7.02E-51 | 1.99E-48 |
| PLEKHG4B | 3.960089 | 1.406629 | 2.54E-06 | 1.68E-05 | MAP1B | -3.37152 | 3.628803 | 1.40E-41 | 2.05E-39 |
| HIST1H2AL | 3.950099 | 1.062457 | 0.000103 | 0.000471 | PGR | -3.36949 | -1.57357 | 1.53E-34 | 1.51E-32 |
| HIST2H2AB | 3.948076 | 1.180573 | 0.000424 | 0.001635 | CSRP1 | -3.36655 | 6.845934 | 4.13E-82 | 1.61E-78 |
| TAC3 | 3.9392 | 6.901288 | 0.004273 | 0.012277 | ABI3BP | -3.36411 | 2.169807 | 2.52E-35 | 2.70E-33 |
| HIST1H4A | 3.935979 | 0.28407 | 0.015938 | 0.037821 | KCNA5 | -3.35873 | -0.61206 | 4.59E-24 | 2.28E-22 |
| ATP4A | 3.935817 | -1.87755 | 0.003312 | 0.009864 | ADCY5 | -3.35229 | 2.180348 | 1.01E-27 | 6.54E-26 |
| AIRE | 3.934096 | 0.500547 | 0.006721 | 0.018135 | CTNNA3 | -3.3514 | -1.67905 | 3.83E-15 | 9.27E-14 |
| HOXC5 | 3.92023 | -2.35765 | 0.003969 | 0.011528 | OTC | -3.35062 | -3.70131 | 0.000684 | 0.00249 |
| UCP1 | 3.919886 | -0.25006 | 0.005749 | 0.015848 | NEXN | -3.35061 | 3.617112 | 2.89E-45 | 5.28E-43 |
| KRT34 | 3.914254 | 1.554632 | 0.008401 | 0.021999 | KY | -3.34814 | -2.64362 | 1.55E-23 | 7.50E-22 |
| MOGAT3 | 3.913686 | -2.84701 | 0.013808 | 0.033459 | ACTA2 | -3.34381 | 8.823143 | 4.85E-36 | 5.25E-34 |
| CST6 | 3.910423 | 7.047595 | 2.83E-07 | 2.29E-06 | RYR2 | -3.34338 | -0.6194 | 2.79E-29 | 1.99E-27 |
| LCT | 3.868911 | -4.02263 | 0.000114 | 0.000515 | TRIM63 | -3.34137 | 0.62727 | 9.67E-14 | 2.03E-12 |
| CLPSL1 | 3.867792 | 0.115266 | 0.004245 | 0.012211 | MAB21L2 | -3.33129 | -2.37647 | 2.97E-07 | 2.40E-06 |
| CXCL5 | 3.860886 | 3.863173 | 0.000166 | 0.000717 | BMP5 | -3.32904 | 1.392423 | 7.13E-15 | 1.69E-13 |
| NXPH4 | 3.85719 | 3.902107 | 6.76E-09 | 7.38E-08 | CRISP3 | -3.3225 | 4.505659 | 2.35E-09 | 2.75E-08 |
| KRT40 | 3.852941 | 0.392026 | 0.004459 | 0.012725 | ANKS1B | -3.31292 | -2.1155 | 8.81E-24 | 4.32E-22 |
| KLHDC7B | 3.84873 | 6.930033 | 8.51E-07 | 6.24E-06 | MICU3 | -3.29635 | -0.10641 | 1.63E-42 | 2.49E-40 |
| SLC39A5 | 3.844425 | 0.596752 | 1.02E-05 | 5.98E-05 | BCHE | -3.29474 | 1.895758 | 1.21E-17 | 3.79E-16 |
| SLC5A5 | 3.842048 | 0.603529 | 2.65E-05 | 0.00014 | TAGLN | -3.29425 | 8.664566 | 2.32E-31 | 1.87E-29 |
| OR7A5 | 3.83825 | -2.48908 | 0.006261 | 0.017052 | TMEM35 | -3.29048 | 2.061929 | 8.07E-17 | 2.32E-15 |
| ACTBL2 | 3.829284 | -1.51001 | 0.009089 | 0.023544 | ENSG00000268434 | -3.29028 | -3.79051 | 2.10E-05 | 0.000114 |
| CNGB1 | 3.821244 | 0.280117 | 2.34E-08 | 2.32E-07 | LRFN5 | -3.28749 | 0.191162 | 2.08E-20 | 8.18E-19 |
| LCN1 | 3.815227 | -1.65978 | 0.003274 | 0.009769 | FLNA | -3.28616 | 8.771541 | 2.74E-51 | 7.90E-49 |
| MIOX | 3.786247 | 0.015364 | 6.61E-06 | 4.00E-05 | PRDM6 | -3.28594 | 1.087361 | 5.86E-35 | 6.10E-33 |
| EVA1A | 3.780234 | 2.664713 | 1.66E-09 | 1.99E-08 | ASPA | -3.28582 | 0.039815 | 1.54E-24 | 8.04E-23 |
| DMRTA2 | 3.772755 | 1.641545 | 5.11E-05 | 0.000253 | TSHB | -3.28006 | -3.09395 | 0.000364 | 0.001429 |
| SIGLEC5 | 3.764145 | 2.433739 | 7.11E-05 | 0.00034 | CFL2 | -3.27866 | 3.506045 | 1.56E-70 | 1.69E-67 |
| AMBP | 3.757589 | 0.680203 | 0.003774 | 0.011059 | ARHGAP20 | -3.27718 | -0.22711 | 3.07E-36 | 3.35E-34 |
| ANKRD34B | 3.747062 | -0.8211 | 0.001637 | 0.005335 | B3GALT2 | -3.27434 | -1.61593 | 8.80E-25 | 4.69E-23 |
| KLK4 | 3.745704 | 1.167667 | 0.009042 | 0.023453 | PPP1R1A | -3.27309 | 0.290751 | 7.81E-15 | 1.83E-13 |
| DEFB126 | 3.735513 | 1.314525 | 0.006641 | 0.017938 | RGS22 | -3.27251 | -2.55976 | 4.37E-19 | 1.57E-17 |
| CCER2 | 3.730334 | 3.671001 | 0.000193 | 0.000817 | SGCD | -3.27182 | 1.076204 | 5.26E-25 | 2.84E-23 |
| SLC10A1 | 3.727855 | 1.267842 | 0.00262 | 0.008038 | TCEAL2 | -3.27151 | 1.774634 | 1.04E-10 | 1.48E-09 |
| SULT1E1 | 3.724577 | 4.028264 | 0.000778 | 0.002784 | SMTN | -3.27051 | 5.318644 | 1.93E-81 | 6.28E-78 |
| HIST1H4D | 3.705064 | 2.525763 | 6.23E-06 | 3.79E-05 | CGNL1 | -3.26305 | 1.875262 | 3.29E-46 | 6.43E-44 |
| RLBP1 | 3.69897 | -1.3615 | 0.013799 | 0.033441 | CCL2 | -3.25531 | 5.725862 | 1.05E-33 | 9.52E-32 |
| KRT31 | 3.695977 | 3.632709 | 0.022102 | 0.049946 | CH25H | -3.25232 | 3.14971 | 4.86E-36 | 5.25E-34 |
| GRIN2D | 3.695386 | 3.989725 | 3.11E-10 | 4.13E-09 | CMA1 | -3.25161 | 1.830612 | 6.49E-09 | 7.10E-08 |
| SLC34A2 | 3.691253 | 2.388335 | 0.009804 | 0.025091 | TPSG1 | -3.24528 | -0.60445 | 1.34E-20 | 5.37E-19 |
| PDX1 | 3.683505 | 0.534843 | 0.003683 | 0.010836 | EPHA7 | -3.24358 | 1.365937 | 3.93E-21 | 1.63E-19 |
| RGAG1 | 3.683479 | 0.074606 | 0.005005 | 0.01408 | CHRM3 | -3.24129 | 0.846872 | 6.92E-21 | 2.80E-19 |
| HEPHL1 | 3.668306 | 2.113512 | 0.000135 | 0.0006 | MAOB | -3.24031 | 4.002645 | 3.44E-22 | 1.53E-20 |
| FXYD4 | 3.657672 | 5.903869 | 0.001692 | 0.005493 | SOBP | -3.23209 | 1.335932 | 9.70E-45 | 1.73E-42 |
| C5orf46 | 3.644693 | 2.095043 | 0.000243 | 0.001 | BAI3 | -3.23198 | -1.0239 | 2.50E-14 | 5.56E-13 |
| RFPL2 | 3.644407 | -2.62536 | 0.011337 | 0.028349 | RCAN2 | -3.23152 | 3.707475 | 1.14E-50 | 3.18E-48 |
| FAM57B | 3.63493 | -0.82841 | 2.18E-05 | 0.000117 | SLURP1 | -3.2277 | 4.429778 | 5.74E-08 | 5.32E-07 |
| SPATA21 | 3.623105 | -2.44706 | 0.004363 | 0.012501 | MAP1A | -3.22663 | 2.505825 | 3.42E-39 | 4.44E-37 |
| VCX | 3.613535 | 1.403775 | 0.00676 | 0.018234 | RP4-533D7.6 | -3.22654 | -10.1233 | 0.021673 | 0.049141 |
| CA9 | 3.612959 | 5.997727 | 2.05E-06 | 1.39E-05 | NKAPL | -3.22413 | -1.11022 | 5.42E-29 | 3.73E-27 |
| HOXC9 | 3.601562 | 2.725008 | 1.48E-06 | 1.04E-05 | LGI1 | -3.21786 | -4.16755 | 3.21E-06 | 2.08E-05 |
| CHIT1 | 3.591829 | 1.426451 | 1.21E-05 | 6.96E-05 | OTOP3 | -3.21609 | -0.58545 | 0.000666 | 0.002432 |
| GTSF1 | 3.591259 | 3.122928 | 0.000241 | 0.000992 | TMEM100 | -3.21563 | 0.769766 | 5.95E-21 | 2.42E-19 |
| CATSPER1 | 3.581457 | 2.149677 | 3.50E-08 | 3.37E-07 | MAS1L | -3.2141 | -3.78644 | 0.001382 | 0.004616 |
| HIST1H2BH | 3.581224 | 4.452642 | 8.96E-08 | 7.98E-07 | ANGPTL1 | -3.20982 | 2.016776 | 7.74E-18 | 2.47E-16 |
| POU4F1 | 3.566143 | -0.49881 | 0.001632 | 0.005324 | GFRA1 | -3.20459 | 0.412083 | 6.08E-18 | 1.96E-16 |
| FOXB1 | 3.565893 | -1.29218 | 0.015929 | 0.037804 | MKX | -3.20381 | 0.312946 | 1.62E-16 | 4.56E-15 |
| MYPN | 3.564601 | -1.13181 | 0.004282 | 0.012297 | DIXDC1 | -3.20294 | 2.418603 | 1.63E-54 | 6.64E-52 |
| ABCG8 | 3.563367 | -3.26579 | 0.011141 | 0.027921 | DACT3 | -3.20292 | 2.460809 | 3.16E-31 | 2.51E-29 |
| EPHA8 | 3.563237 | -2.851 | 0.006966 | 0.018683 | GEM | -3.20183 | 4.704685 | 2.34E-34 | 2.25E-32 |
| RP11-315D16.2 | 3.545452 | -2.7052 | 0.009256 | 0.023879 | PDLIM3 | -3.20113 | 4.173005 | 6.02E-28 | 3.93E-26 |
| HIST2H2BF | 3.539733 | 1.821918 | 3.75E-11 | 5.67E-10 | CHRDL1 | -3.19949 | 2.670223 | 1.43E-10 | 2.00E-09 |
| HIST1H2BE | 3.537255 | 1.607422 | 1.54E-08 | 1.57E-07 | NECAB1 | -3.19819 | 0.76487 | 6.88E-30 | 5.16E-28 |
| MSLN | 3.531325 | 4.738783 | 0.000176 | 0.000753 | GYS2 | -3.19212 | -2.65732 | 4.37E-13 | 8.47E-12 |
| CDK5R2 | 3.525116 | -1.00932 | 0.003957 | 0.011503 | NKX6-2 | -3.18944 | -4.89938 | 7.47E-06 | 4.47E-05 |
| NOTUM | 3.51561 | 5.186167 | 0.001862 | 0.005975 | CACNB2 | -3.18741 | 0.832092 | 2.10E-41 | 3.04E-39 |
| DLL3 | 3.50341 | 0.692828 | 0.000124 | 0.000555 | CCL14 | -3.18261 | 0.167674 | 1.22E-15 | 3.14E-14 |
| HIST1H2BG | 3.49924 | 4.09204 | 6.13E-09 | 6.74E-08 | CALD1 | -3.1769 | 6.514307 | 5.09E-47 | 1.10E-44 |
| SCGB3A2 | 3.481708 | -0.36147 | 0.010381 | 0.026322 | ADAM33 | -3.1718 | 2.081927 | 2.82E-27 | 1.78E-25 |
| HIST1H2AE | 3.480358 | 4.730379 | 1.57E-10 | 2.19E-09 | CILP | -3.17122 | 3.770672 | 1.34E-14 | 3.05E-13 |
| SYNGR4 | 3.47601 | -0.05279 | 5.41E-06 | 3.34E-05 | KCNJ3 | -3.16911 | -1.97543 | 8.95E-08 | 7.98E-07 |
| MUCL1 | 3.474334 | 3.153151 | 0.005423 | 0.01508 | P2RY14 | -3.16675 | 0.99341 | 7.91E-40 | 1.10E-37 |
| HMP19 | 3.467205 | -1.07713 | 0.021148 | 0.048112 | PLA2G5 | -3.1658 | 1.555673 | 3.55E-24 | 1.79E-22 |
| HNF4A | 3.458752 | 0.00741 | 0.001325 | 0.004448 | PRIMA1 | -3.16136 | 0.738433 | 3.78E-14 | 8.26E-13 |
| GBX2 | 3.451147 | -2.06974 | 0.001704 | 0.005526 | HIF3A | -3.15799 | 0.992181 | 2.40E-17 | 7.26E-16 |
| PCSK1N | 3.444532 | 3.302614 | 2.06E-05 | 0.000112 | PALLD | -3.15329 | 5.89011 | 8.81E-53 | 2.92E-50 |
| TCF24 | 3.442263 | -0.86408 | 3.77E-09 | 4.28E-08 | SPEG | -3.15302 | 1.822663 | 5.77E-27 | 3.55E-25 |
| CLEC18C | 3.430437 | -2.65135 | 0.014369 | 0.034618 | NR3C2 | -3.14249 | 1.381806 | 3.80E-29 | 2.65E-27 |
| TERT | 3.429099 | 0.921163 | 1.89E-07 | 1.58E-06 | CCDC27 | -3.14179 | -4.31724 | 2.81E-05 | 0.000148 |
| TAGLN3 | 3.415336 | 1.087528 | 0.002765 | 0.008429 | SPARCL1 | -3.13937 | 7.172746 | 1.43E-43 | 2.35E-41 |
| LRRN4 | 3.415161 | -0.81004 | 3.22E-05 | 0.000167 | IL13 | -3.13646 | -3.25174 | 9.51E-09 | 1.02E-07 |
| CDX2 | 3.411952 | 1.116629 | 0.005389 | 0.015004 | HSPB8 | -3.13599 | 6.503128 | 1.67E-26 | 9.99E-25 |
| CXorf22 | 3.402396 | -0.57743 | 0.000175 | 0.000751 | RBM24 | -3.13305 | 0.850978 | 2.01E-18 | 6.82E-17 |
| HIST1H3D | 3.398519 | 3.82821 | 5.29E-12 | 8.96E-11 | FOXF1 | -3.13135 | 3.868938 | 5.70E-41 | 8.14E-39 |
| LHX9 | 3.396528 | -2.72056 | 0.000879 | 0.003096 | TPM2 | -3.12905 | 8.005026 | 3.03E-34 | 2.86E-32 |
| HIST1H3C | 3.395418 | 1.697226 | 2.14E-05 | 0.000115 | DCHS2 | -3.12857 | -2.85528 | 2.78E-14 | 6.13E-13 |
| CDX1 | 3.39013 | 0.963293 | 0.000175 | 0.000751 | MFAP4 | -3.12503 | 6.928241 | 7.43E-26 | 4.27E-24 |
| DMRT2 | 3.388788 | 0.848756 | 0.001091 | 0.003748 | PPP1R14A | -3.12493 | 4.732058 | 8.61E-38 | 1.03E-35 |
| MNX1 | 3.387426 | 0.564806 | 1.34E-07 | 1.15E-06 | ATF3 | -3.12486 | 6.464697 | 1.50E-34 | 1.49E-32 |
| IGF2 | 3.367252 | 10.19964 | 0.000355 | 0.001395 | MYH3 | -3.12144 | 1.694265 | 1.97E-29 | 1.44E-27 |
| CXCL11 | 3.356734 | 5.384193 | 5.14E-05 | 0.000254 | C11orf96 | -3.12138 | 5.871101 | 1.53E-34 | 1.51E-32 |
| IL36G | 3.350721 | 3.029606 | 0.004424 | 0.012644 | SRL | -3.12108 | -1.25059 | 9.11E-33 | 7.79E-31 |
| KISS1 | 3.337301 | 4.774586 | 0.000209 | 0.000876 | GREM2 | -3.11392 | 0.717837 | 9.80E-14 | 2.05E-12 |
| MYRFL | 3.337157 | -0.38555 | 1.44E-05 | 8.12E-05 | CNFN | -3.11126 | 6.798889 | 3.06E-19 | 1.12E-17 |
| IGDCC3 | 3.334652 | 1.020193 | 0.000746 | 0.002687 | CNTN1 | -3.11126 | 2.964703 | 1.28E-14 | 2.93E-13 |
| PM20D1 | 3.332869 | 6.429922 | 0.001832 | 0.005898 | RASL12 | -3.11077 | 3.731022 | 1.21E-39 | 1.62E-37 |
| CLDN2 | 3.331781 | -0.6035 | 2.49E-05 | 0.000132 | SVEP1 | -3.10963 | 1.507907 | 4.92E-45 | 8.92E-43 |
| LRRC15 | 3.330432 | 3.222094 | 3.18E-05 | 0.000164 | pk | -3.10946 | 3.891254 | 1.39E-65 | 1.13E-62 |
| PNMT | 3.324203 | 4.854213 | 0.000159 | 0.00069 | SLIT3 | -3.10929 | 3.325823 | 3.45E-35 | 3.63E-33 |
| GRM4 | 3.316797 | -2.25683 | 0.000164 | 0.00071 | OMD | -3.10834 | 1.078525 | 1.37E-10 | 1.93E-09 |
| KRT9 | 3.309829 | 1.035757 | 9.12E-05 | 0.000424 | GPRASP1 | -3.10589 | 1.804459 | 1.03E-52 | 3.35E-50 |
| ACTL8 | 3.295185 | 1.024848 | 0.015647 | 0.037234 | ZNF728 | -3.10115 | -3.61843 | 0.000306 | 0.001224 |
| FAM19A3 | 3.290607 | -0.22376 | 1.65E-05 | 9.20E-05 | HAS1 | -3.09616 | 2.196461 | 4.72E-12 | 8.06E-11 |
| ATP6V0D2 | 3.286815 | -0.09056 | 2.72E-05 | 0.000143 | MTTP | -3.09502 | -2.14789 | 5.58E-17 | 1.63E-15 |
| C19orf67 | 3.285278 | -1.90703 | 2.96E-05 | 0.000155 | PID1 | -3.09446 | 2.154986 | 1.21E-32 | 1.03E-30 |
| SLC30A10 | 3.274735 | -2.60069 | 0.004503 | 0.012838 | FAM46B | -3.09296 | 4.000341 | 1.62E-24 | 8.40E-23 |
| IGSF23 | 3.268268 | -2.31997 | 0.010445 | 0.026468 | MRVI1 | -3.08775 | 3.60044 | 4.81E-38 | 5.88E-36 |
| FOXL2 | 3.263406 | 1.282556 | 0.000111 | 0.000503 | ACKR1 | -3.085 | 5.040445 | 2.10E-17 | 6.40E-16 |
| FSTL5 | 3.256297 | -0.57859 | 0.017798 | 0.041515 | DUSP1 | -3.08228 | 8.763138 | 6.38E-42 | 9.53E-40 |
| FBN2 | 3.245759 | 3.073964 | 7.87E-06 | 4.69E-05 | GABRG1 | -3.07808 | -6.29854 | 0.002129 | 0.006712 |
| TMEM213 | 3.24187 | -3.09672 | 0.005389 | 0.015004 | ADAMTS8 | -3.07578 | 1.119235 | 3.58E-21 | 1.49E-19 |
| HDGFL1 | 3.240311 | -2.01223 | 0.019877 | 0.045624 | LRRTM1 | -3.07377 | -3.57284 | 3.34E-05 | 0.000172 |
| LPO | 3.234453 | -3.45104 | 0.000194 | 0.00082 | FOS | -3.06344 | 8.978161 | 2.08E-38 | 2.59E-36 |
| PDZK1 | 3.23206 | 0.695563 | 1.06E-06 | 7.66E-06 | MMRN1 | -3.06192 | 1.125715 | 9.22E-21 | 3.72E-19 |
| HIST1H3J | 3.22422 | -0.31728 | 0.000941 | 0.003294 | ASTL | -3.05868 | 0.235188 | 6.18E-15 | 1.47E-13 |
| OTOF | 3.223086 | 0.45484 | 2.07E-06 | 1.40E-05 | LONRF2 | -3.05264 | 0.1999 | 4.92E-15 | 1.18E-13 |
| ZPLD1 | 3.21512 | -2.30551 | 0.00186 | 0.005974 | FOXP2 | -3.05098 | 0.101177 | 3.94E-23 | 1.87E-21 |
| MYT1 | 3.215039 | 1.588049 | 0.001064 | 0.003663 | FXYD1 | -3.04921 | 0.081328 | 1.21E-27 | 7.71E-26 |
| CACNA1E | 3.214329 | -2.93194 | 0.000901 | 0.003166 | PDZRN3 | -3.0487 | 2.899991 | 2.62E-29 | 1.89E-27 |
| SERPINB7 | 3.212711 | 2.929772 | 0.007783 | 0.020602 | NDNF | -3.04576 | 1.148353 | 1.27E-18 | 4.40E-17 |
| SEZ6 | 3.205704 | -1.22944 | 0.003633 | 0.010704 | PRKG1 | -3.0434 | 1.584101 | 1.44E-36 | 1.62E-34 |
| MAGEB17 | 3.202606 | -0.53814 | 0.009365 | 0.024127 | CTGF | -3.04042 | 8.016586 | 1.59E-30 | 1.21E-28 |
| ITLN2 | 3.201704 | 1.14875 | 0.008896 | 0.023121 | PLCB4 | -3.04011 | 1.875851 | 4.47E-27 | 2.78E-25 |
| GRM3 | 3.201568 | -0.01692 | 0.001431 | 0.004754 | TBX4 | -3.03576 | 1.425245 | 4.03E-17 | 1.20E-15 |
| CALHM1 | 3.188619 | -1.27001 | 0.002808 | 0.008541 | MRGPRF | -3.03375 | 4.263309 | 2.84E-29 | 2.02E-27 |
| RHBG | 3.18392 | 3.688414 | 2.87E-05 | 0.00015 | CACNA1C | -3.03307 | 1.129794 | 7.47E-35 | 7.65E-33 |
| KRT33B | 3.183354 | 1.356237 | 0.006199 | 0.016911 | HMGCLL1 | -3.03224 | -2.43921 | 7.78E-10 | 9.88E-09 |
| SALL4 | 3.17761 | 2.024552 | 3.35E-10 | 4.43E-09 | TNNT2 | -3.02733 | 1.604134 | 6.10E-22 | 2.68E-20 |
| ERVW-1 | 3.172146 | -1.49913 | 0.010808 | 0.027211 | RYR3 | -3.02589 | -1.38926 | 2.04E-56 | 9.75E-54 |
| LMX1B | 3.16988 | -1.02006 | 0.003581 | 0.010575 | NLGN1 | -3.01671 | -1.05117 | 6.34E-17 | 1.84E-15 |
| PKLR | 3.163853 | -2.15577 | 0.008784 | 0.022877 | ANK2 | -3.01146 | 0.400886 | 1.78E-20 | 7.07E-19 |
| TBR1 | 3.163383 | -3.36822 | 0.007315 | 0.019495 | IL36A | -3.00479 | -1.3827 | 0.018044 | 0.042002 |
| VGF | 3.162988 | 1.850816 | 1.30E-06 | 9.23E-06 | ADCYAP1R1 | -2.99785 | -1.8019 | 3.21E-15 | 7.83E-14 |
| MMP9 | 3.15856 | 7.393658 | 0.00016 | 0.000692 | RAB23 | -2.99769 | 3.131505 | 4.16E-48 | 9.81E-46 |
| KRT83 | 3.153852 | 1.104883 | 0.002846 | 0.008634 | ZEB1 | -2.99416 | 3.007064 | 9.19E-48 | 2.14E-45 |
| DCSTAMP | 3.153334 | -0.74888 | 0.003872 | 0.011294 | GPM6A | -2.98961 | -1.726 | 4.96E-12 | 8.44E-11 |
| GPC2 | 3.140216 | 2.391748 | 1.19E-08 | 1.25E-07 | PLAC9 | -2.98826 | 3.69776 | 9.20E-34 | 8.42E-32 |
| ASCL5 | 3.132554 | -0.03026 | 6.88E-05 | 0.00033 | PDE1A | -2.98226 | 1.020901 | 7.10E-33 | 6.12E-31 |
| NKX3-2 | 3.125879 | -0.72399 | 5.79E-06 | 3.55E-05 | CYR61 | -2.97606 | 8.113287 | 7.21E-35 | 7.43E-33 |
| WNT7A | 3.124007 | 2.735978 | 0.000263 | 0.001075 | TBX5 | -2.97591 | 0.692632 | 7.42E-23 | 3.47E-21 |
| INSRR | 3.122886 | -1.07819 | 0.015079 | 0.036058 | CTSG | -2.96575 | 2.858898 | 1.56E-11 | 2.49E-10 |
| HOXC6 | 3.114212 | 1.798513 | 3.94E-07 | 3.10E-06 | SFRP5 | -2.96302 | -0.61136 | 1.16E-05 | 6.68E-05 |
| ULBP1 | 3.11306 | 0.229919 | 9.36E-09 | 1.00E-07 | SMOC2 | -2.96262 | 4.854806 | 5.69E-24 | 2.81E-22 |
| HIST1H2BJ | 3.111359 | 4.088467 | 2.16E-10 | 2.94E-09 | NMRK2 | -2.959 | -3.21456 | 0.000559 | 0.002085 |
| LGR5 | 3.109218 | 0.796186 | 0.000464 | 0.00177 | ADAMTS4 | -2.95724 | 3.649192 | 8.79E-31 | 6.83E-29 |
| FCRLB | 3.108665 | 4.587022 | 4.03E-07 | 3.17E-06 | KIAA1210 | -2.95108 | -3.67066 | 1.45E-08 | 1.49E-07 |
| CYP26A1 | 3.087145 | 0.346842 | 0.00362 | 0.010672 | SVIL | -2.95041 | 5.375499 | 2.03E-60 | 1.24E-57 |
| NTS | 3.085662 | 4.366683 | 0.011674 | 0.029043 | SCN2B | -2.94129 | -2.33908 | 2.85E-09 | 3.31E-08 |
| TMEM145 | 3.084689 | 0.866759 | 3.18E-07 | 2.54E-06 | EPHB1 | -2.94104 | 0.079828 | 2.08E-22 | 9.46E-21 |
| TMEM249 | 3.08212 | -1.44206 | 4.65E-09 | 5.23E-08 | CPXM2 | -2.93678 | 3.785629 | 4.34E-22 | 1.92E-20 |
| WDR66 | 3.078802 | 1.213479 | 2.10E-07 | 1.74E-06 | ADRA1D | -2.93285 | -0.9111 | 9.69E-17 | 2.78E-15 |
| ATP6V1B1 | 3.078657 | 2.591898 | 7.80E-06 | 4.65E-05 | MASP1 | -2.92947 | -0.87475 | 2.35E-18 | 7.89E-17 |
| MT1H | 3.071256 | 2.409105 | 0.000307 | 0.001227 | CLIP3 | -2.92401 | 4.184328 | 3.04E-31 | 2.45E-29 |
| CACNA1B | 3.060786 | -1.65407 | 0.005569 | 0.015432 | ABRA | -2.91792 | -2.971 | 3.97E-11 | 5.97E-10 |
| CSAG1 | 3.056714 | 4.65327 | 0.020338 | 0.04653 | PLCD4 | -2.91697 | 1.184772 | 7.16E-75 | 9.34E-72 |
| RGS20 | 3.047973 | 1.806257 | 5.64E-07 | 4.31E-06 | ODF3L1 | -2.91103 | 0.594662 | 2.44E-24 | 1.25E-22 |
| PNPLA3 | 3.047339 | -0.80698 | 1.90E-05 | 0.000104 | GNAL | -2.90758 | 1.617431 | 8.99E-38 | 1.07E-35 |
| HOXC8 | 3.046851 | 0.93969 | 0.000163 | 0.000706 | SH3BGR | -2.90613 | 3.285247 | 2.07E-58 | 1.16E-55 |
| SLC13A5 | 3.035768 | -1.511 | 0.000323 | 0.001285 | PRRG3 | -2.90524 | -1.43816 | 3.87E-15 | 9.37E-14 |
| RDM1 | 3.035633 | 2.089527 | 2.04E-11 | 3.18E-10 | ITGA7 | -2.90246 | 3.748263 | 2.68E-29 | 1.92E-27 |
| OTP | 3.033261 | -3.98706 | 0.012193 | 0.030167 | ESR1 | -2.88759 | 0.63778 | 6.16E-22 | 2.70E-20 |
| PNLDC1 | 3.032376 | 0.723796 | 0.001489 | 0.004915 | COL14A1 | -2.87404 | 4.166674 | 2.42E-26 | 1.43E-24 |
| RNASE7 | 3.03206 | 3.292148 | 0.000104 | 0.000473 | PCSK2 | -2.87253 | -1.02904 | 5.36E-05 | 0.000263 |
| SULT4A1 | 3.027265 | 0.768476 | 0.014567 | 0.035001 | ZFP36 | -2.87222 | 9.127029 | 2.89E-46 | 5.72E-44 |
| TLX2 | 3.001609 | -0.54111 | 1.45E-05 | 8.16E-05 | LPP | -2.86959 | 4.358025 | 4.00E-71 | 4.61E-68 |
| CELF5 | 2.998282 | -1.17382 | 0.000141 | 0.000621 | EMP1 | -2.86864 | 6.269891 | 1.99E-34 | 1.92E-32 |
| CHRNA6 | 2.996402 | -1.38294 | 0.006505 | 0.017617 | DNAJB5 | -2.8664 | 3.01524 | 8.53E-40 | 1.16E-37 |
| MKRN3 | 2.996142 | 0.176975 | 0.004013 | 0.011635 | LCN6 | -2.86549 | -1.0436 | 3.43E-07 | 2.72E-06 |
| PIWIL1 | 2.995722 | -2.14111 | 0.018869 | 0.043694 | SOX17 | -2.86409 | 2.145892 | 2.20E-39 | 2.89E-37 |
| KISS1R | 2.994958 | 0.210548 | 0.000706 | 0.002556 | FERMT2 | -2.86041 | 3.88704 | 8.52E-40 | 1.16E-37 |
| GLP1R | 2.993751 | -0.82787 | 0.010121 | 0.025739 | CDH19 | -2.85888 | -0.23172 | 7.46E-07 | 5.54E-06 |
| GRIN3B | 2.993152 | -0.27909 | 8.28E-09 | 8.93E-08 | NR4A2 | -2.85829 | 4.209672 | 2.24E-36 | 2.46E-34 |
| DRAXIN | 2.986339 | -0.24555 | 5.40E-07 | 4.14E-06 | KLF2 | -2.85539 | 4.974522 | 1.21E-41 | 1.80E-39 |
| PPP4R4 | 2.983945 | -0.20543 | 0.001541 | 0.005066 | FGF9 | -2.85467 | -0.79492 | 1.92E-09 | 2.28E-08 |
| ABCA4 | 2.981492 | 1.891377 | 0.000144 | 0.000633 | IL1RAPL1 | -2.852 | -3.1094 | 2.84E-07 | 2.30E-06 |
| TENM2 | 2.980312 | 2.358573 | 0.004159 | 0.012004 | PAMR1 | -2.85009 | 3.147959 | 4.93E-40 | 6.93E-38 |
| SLC45A2 | 2.974699 | -1.5048 | 0.000606 | 0.002234 | GPIHBP1 | -2.84873 | 1.422861 | 5.85E-21 | 2.39E-19 |
| SHISA2 | 2.972716 | 4.129479 | 0.000163 | 0.000705 | EPHA6 | -2.84704 | -1.96114 | 1.16E-08 | 1.22E-07 |
| MUC6 | 2.972463 | -0.29811 | 0.004107 | 0.011877 | GATA6 | -2.84356 | 2.599576 | 2.07E-28 | 1.39E-26 |
| GREB1L | 2.972197 | -0.44722 | 5.33E-05 | 0.000262 | RGS2 | -2.84304 | 6.429205 | 1.86E-30 | 1.42E-28 |
| METTL11B | 2.960072 | -1.5371 | 0.004297 | 0.012329 | KLHL33 | -2.84276 | -3.36098 | 0.000145 | 0.000635 |
| PRAP1 | 2.957844 | 3.73187 | 0.00109 | 0.003745 | KANK2 | -2.84126 | 5.216129 | 2.92E-55 | 1.24E-52 |
| KCNG1 | 2.957706 | 4.709339 | 3.83E-06 | 2.46E-05 | C3orf70 | -2.84041 | 2.021305 | 6.55E-30 | 4.93E-28 |
| ADAMTS20 | 2.957044 | -1.00163 | 0.006997 | 0.018754 | TRPC3 | -2.83889 | -0.71083 | 5.99E-21 | 2.43E-19 |
| TMEM191B | 2.956119 | -0.06986 | 2.24E-05 | 0.00012 | LDB3 | -2.8382 | 0.340154 | 3.69E-24 | 1.85E-22 |
| ENSG00000183248 | 2.955673 | 3.402164 | 1.90E-06 | 1.30E-05 | SHISA6 | -2.83746 | -1.78764 | 4.39E-10 | 5.73E-09 |
| PDIA2 | 2.955122 | 0.589275 | 3.37E-05 | 0.000174 | LYVE1 | -2.83355 | 2.784249 | 9.92E-17 | 2.84E-15 |
| CSTL1 | 2.954024 | -0.93992 | 0.001253 | 0.004233 | ADCYAP1 | -2.83006 | -0.02276 | 8.36E-13 | 1.56E-11 |
| SAA2 | 2.952562 | 3.462915 | 0.001362 | 0.004555 | NFASC | -2.82544 | 0.929512 | 3.24E-32 | 2.70E-30 |
| DMRT3 | 2.952432 | -1.40472 | 0.016447 | 0.038849 | BVES | -2.82341 | 1.881686 | 9.93E-22 | 4.32E-20 |
| ATP12A | 2.95216 | 1.168527 | 0.013142 | 0.032101 | FAXDC2 | -2.82169 | 1.80682 | 1.16E-43 | 1.93E-41 |
| AL589743.1 | 2.951285 | 0.48257 | 4.12E-05 | 0.000208 | SFRP1 | -2.81953 | 3.64358 | 1.25E-12 | 2.28E-11 |
| AMH | 2.947281 | 2.20955 | 2.41E-06 | 1.61E-05 | SLIT2 | -2.81523 | 1.441473 | 2.84E-19 | 1.04E-17 |
| HIST1H3H | 2.94642 | 4.21243 | 2.60E-08 | 2.56E-07 | DMGDH | -2.81277 | -1.59222 | 9.68E-41 | 1.37E-38 |
| SLC24A2 | 2.945467 | -1.77627 | 1.90E-06 | 1.30E-05 | FGF2 | -2.81209 | 1.458126 | 2.19E-22 | 9.95E-21 |
| CACNG4 | 2.944953 | 3.670984 | 0.000269 | 0.001093 | HBEGF | -2.80536 | 5.543752 | 1.59E-33 | 1.42E-31 |
| CELF3 | 2.943777 | -1.28397 | 0.013464 | 0.032736 | GALNT15 | -2.80166 | 1.406342 | 6.42E-20 | 2.45E-18 |
| PTPRN | 2.941911 | -0.35912 | 0.0001 | 0.000461 | ITPR1 | -2.79863 | 2.746603 | 6.17E-45 | 1.11E-42 |
| TMEM88B | 2.939276 | 0.366325 | 0.012859 | 0.031511 | TMEM132D | -2.79569 | -6.33502 | 0.003708 | 0.010902 |
| CHRNB2 | 2.9346 | -1.3562 | 0.000279 | 0.001127 | CKMT2 | -2.79334 | 0.258684 | 1.13E-30 | 8.67E-29 |
| ENSG00000271698 | 2.933272 | -1.93778 | 3.52E-06 | 2.26E-05 | SHISA3 | -2.79328 | 0.18661 | 6.59E-07 | 4.95E-06 |
| ZNF556 | 2.93205 | 0.062098 | 0.00017 | 0.000731 | KCND3 | -2.79277 | 1.586677 | 1.47E-19 | 5.50E-18 |
| TMEM74B | 2.928657 | 3.376572 | 9.35E-10 | 1.17E-08 | NOVA1 | -2.78721 | -0.63863 | 3.28E-17 | 9.81E-16 |
| TFF2 | 2.927623 | 4.428407 | 0.020421 | 0.046695 | CACNB4 | -2.78588 | -0.64181 | 8.25E-19 | 2.90E-17 |
| CCR3 | 2.925249 | -0.00138 | 7.02E-07 | 5.23E-06 | NT5DC3 | -2.7832 | 2.378287 | 1.04E-55 | 4.53E-53 |
| HIST1H2AM | 2.923687 | 1.740349 | 2.50E-06 | 1.66E-05 | DDR2 | -2.78253 | 2.936988 | 8.82E-28 | 5.71E-26 |
| SH2D5 | 2.919869 | 0.78675 | 8.46E-05 | 0.000397 | ENPP6 | -2.78221 | -1.27254 | 7.47E-16 | 1.97E-14 |
| DMBX1 | 2.915373 | 2.651441 | 0.000229 | 0.000949 | JAM3 | -2.78029 | 3.728895 | 1.24E-37 | 1.45E-35 |
| KLF14 | 2.913573 | -1.00203 | 0.003007 | 0.009052 | NBEA | -2.77993 | 0.999374 | 1.98E-21 | 8.44E-20 |
| TRPM8 | 2.913493 | -1.96117 | 0.00199 | 0.006319 | GNG7 | -2.77931 | 1.70688 | 8.18E-31 | 6.38E-29 |
| LPPR3 | 2.910441 | -1.30603 | 0.010086 | 0.025669 | THBS1 | -2.77882 | 7.169865 | 5.36E-29 | 3.71E-27 |
| SLCO6A1 | 2.908043 | -1.21812 | 0.02197 | 0.049682 | ZFPM2 | -2.77579 | 0.890943 | 6.00E-27 | 3.67E-25 |
| CYP4F2 | 2.905993 | 1.183386 | 0.016732 | 0.039424 | HHIP | -2.77348 | -0.17201 | 1.82E-12 | 3.25E-11 |
| KIF18B | 2.903271 | 4.166489 | 2.18E-19 | 8.12E-18 | HSPB2-C11orf52 | -2.77153 | -6.66105 | 0.011064 | 0.027762 |
| HIST2H3D | 2.898411 | 0.546726 | 0.000244 | 0.001003 | LCN10 | -2.76699 | -3.10397 | 4.46E-05 | 0.000223 |
| S100A3 | 2.894198 | 6.4632 | 0.000549 | 0.002051 | LCN8 | -2.76577 | -4.72969 | 0.003182 | 0.009526 |
| TRIM72 | 2.894088 | -1.81153 | 0.000275 | 0.001113 | ACACB | -2.76395 | 1.723876 | 5.56E-42 | 8.37E-40 |
| CXCL10 | 2.892984 | 7.40919 | 6.94E-05 | 0.000333 | DCT | -2.76334 | -4.84608 | 1.35E-05 | 7.68E-05 |
| MMP1 | 2.89194 | 7.649237 | 0.000137 | 0.000605 | CSRNP1 | -2.75502 | 5.615473 | 5.01E-61 | 3.27E-58 |
| LRP2 | 2.889451 | -1.90445 | 0.000249 | 0.001019 | STAB2 | -2.75389 | -2.85713 | 3.68E-18 | 1.21E-16 |
| FOXH1 | 2.887475 | 0.669989 | 1.79E-05 | 9.85E-05 | SCRG1 | -2.75263 | 0.447875 | 1.23E-08 | 1.29E-07 |
| CNTN5 | 2.886097 | -1.51039 | 0.018404 | 0.04277 | CCDC69 | -2.75257 | 4.825412 | 3.31E-44 | 5.69E-42 |
| TMEM151A | 2.883589 | -0.55742 | 0.001144 | 0.003906 | NXPH3 | -2.74783 | 1.502242 | 3.42E-22 | 1.53E-20 |
| NHLH1 | 2.883095 | 0.468805 | 0.000483 | 0.001831 | TMPRSS11F | -2.74507 | -0.80686 | 0.002782 | 0.008472 |
| ELAVL2 | 2.861951 | 0.492063 | 0.00058 | 0.00215 | MYOZ2 | -2.7448 | -3.3166 | 9.21E-07 | 6.71E-06 |
| MAPK15 | 2.860964 | 3.259431 | 2.83E-08 | 2.76E-07 | MAPT | -2.74228 | 0.125401 | 1.17E-19 | 4.44E-18 |
| KANK4 | 2.860646 | 0.885474 | 0.002274 | 0.007109 | FAM189A2 | -2.74009 | 1.513233 | 4.40E-26 | 2.56E-24 |
| MUC3A | 2.855756 | 1.597915 | 0.000258 | 0.001055 | RAI2 | -2.73952 | 3.101428 | 3.27E-31 | 2.59E-29 |
| SLC12A3 | 2.852979 | -0.04361 | 0.00353 | 0.010443 | AKAP2 | -2.73697 | -2.81426 | 1.75E-12 | 3.15E-11 |
| DLX2 | 2.849853 | -1.15085 | 0.004585 | 0.013052 | ACOX2 | -2.73681 | 1.029988 | 1.48E-23 | 7.17E-22 |
| HIST3H2BB | 2.848993 | 1.646257 | 1.33E-09 | 1.62E-08 | MYOT | -2.7327 | -0.6501 | 9.89E-18 | 3.13E-16 |
| SMIM24 | 2.844482 | 1.642916 | 2.11E-05 | 0.000114 | CTD-2207O23.3 | -2.73169 | -5.42356 | 0.004342 | 0.012448 |
| SMC1B | 2.837502 | 1.11086 | 9.79E-07 | 7.10E-06 | LRRN4CL | -2.73087 | 1.978101 | 1.59E-25 | 8.92E-24 |
| ECEL1 | 2.836869 | 1.204832 | 0.000264 | 0.001078 | ROR1 | -2.72709 | 0.641065 | 1.19E-21 | 5.17E-20 |
| MMP8 | 2.836231 | -3.0959 | 0.020044 | 0.045953 | MYOM2 | -2.72563 | -0.53626 | 5.06E-27 | 3.14E-25 |
| NRCAM | 2.835894 | 1.996955 | 1.69E-05 | 9.38E-05 | PRKAA2 | -2.72467 | 0.412197 | 2.01E-17 | 6.16E-16 |
| FSD1 | 2.83525 | 2.365708 | 1.67E-05 | 9.27E-05 | ZCCHC24 | -2.72119 | 4.038936 | 1.22E-36 | 1.38E-34 |
| NKX2-8 | 2.829342 | 1.228044 | 0.016811 | 0.039577 | PARK2 | -2.71698 | 0.075507 | 2.39E-29 | 1.73E-27 |
| ST8SIA2 | 2.827449 | -1.1877 | 0.0034 | 0.010092 | C8orf88 | -2.7165 | 2.528234 | 2.87E-16 | 7.94E-15 |
| GNG8 | 2.826781 | 4.062237 | 0.020124 | 0.04611 | PKDCC | -2.71434 | 3.264013 | 3.13E-14 | 6.89E-13 |
| SERPINA1 | 2.817995 | 5.687449 | 1.53E-05 | 8.59E-05 | RERG | -2.71242 | 2.037082 | 1.05E-26 | 6.32E-25 |
| RBP3 | 2.817019 | -1.81554 | 0.004379 | 0.012536 | RNF222 | -2.70898 | -0.40905 | 3.05E-11 | 4.66E-10 |
| SFTPB | 2.816824 | 2.089036 | 0.021341 | 0.048502 | KLF9 | -2.7047 | 4.363896 | 2.34E-39 | 3.06E-37 |
| ALDOB | 2.812127 | 0.267403 | 0.008704 | 0.0227 | PDZD4 | -2.70365 | 1.571622 | 2.18E-18 | 7.39E-17 |
| RAC3 | 2.807507 | 4.994805 | 8.40E-14 | 1.77E-12 | SLC25A25 | -2.70117 | 3.965737 | 9.38E-67 | 8.74E-64 |
| C9orf171 | 2.806646 | -0.71979 | 0.002855 | 0.008654 | FABP12 | -2.6975 | -2.10361 | 0.013364 | 0.032546 |
| FOXD1 | 2.805822 | 2.231968 | 0.000312 | 0.001245 | ITGB1BP2 | -2.69619 | 1.33869 | 1.05E-39 | 1.42E-37 |
| CCNA1 | 2.805176 | 2.015066 | 0.01102 | 0.027685 | CCDC80 | -2.69044 | 4.661085 | 2.11E-17 | 6.43E-16 |
| LAMC2 | 2.803296 | 7.355989 | 1.02E-05 | 5.98E-05 | KCNK3 | -2.69019 | 1.170435 | 3.95E-13 | 7.68E-12 |
| KRT86 | 2.802534 | 3.603855 | 0.000132 | 0.000587 | EPHA3 | -2.68466 | 1.888588 | 5.96E-24 | 2.94E-22 |
| B4GALNT4 | 2.802246 | 3.969762 | 1.45E-05 | 8.16E-05 | RUNX1T1 | -2.68169 | -0.48426 | 3.53E-21 | 1.48E-19 |
| CSF2 | 2.802035 | 2.259031 | 0.000662 | 0.002418 | HTR1B | -2.676 | -0.4458 | 1.54E-08 | 1.58E-07 |
| CDH3 | 2.799086 | 6.619089 | 7.70E-07 | 5.70E-06 | JAM2 | -2.67576 | 2.002362 | 9.21E-34 | 8.42E-32 |
| DLX6 | 2.783615 | 1.599466 | 3.33E-05 | 0.000171 | COX7A1 | -2.67522 | 4.486347 | 5.69E-32 | 4.68E-30 |
| RSPO4 | 2.782977 | 2.178395 | 0.001082 | 0.00372 | GADD45B | -2.67178 | 6.547291 | 1.47E-34 | 1.47E-32 |
| HOXC13 | 2.780571 | 2.623504 | 0.000101 | 0.000462 | CRCT1 | -2.67001 | 5.33587 | 1.14E-05 | 6.59E-05 |
| RNF182 | 2.778301 | 0.889454 | 0.000169 | 0.000729 | EPM2A | -2.6677 | 1.568349 | 1.63E-75 | 2.66E-72 |
| FAM101A | 2.778151 | 2.125986 | 0.000233 | 0.000966 | BAG2 | -2.66689 | 3.09077 | 3.57E-35 | 3.73E-33 |
| OLR1 | 2.771763 | 4.775507 | 4.42E-05 | 0.000221 | UGT1A7 | -2.66597 | -0.29052 | 1.19E-09 | 1.46E-08 |
| FAM72D | 2.7717 | 0.946646 | 6.78E-15 | 1.61E-13 | HSD17B6 | -2.66305 | 3.14044 | 9.86E-25 | 5.25E-23 |
| MSI1 | 2.770684 | 2.435523 | 2.83E-05 | 0.000149 | RSPO2 | -2.66198 | -0.31601 | 1.41E-05 | 7.95E-05 |
| NAT16 | 2.77052 | -3.26054 | 0.001895 | 0.006063 | PDE2A | -2.66051 | 2.028256 | 5.71E-31 | 4.47E-29 |
| F2 | 2.766774 | -0.3655 | 0.016512 | 0.038992 | ANKRD62 | -2.6585 | -3.99802 | 2.93E-05 | 0.000153 |
| CYP3A43 | 2.765513 | -2.50763 | 0.015321 | 0.036575 | CELF2 | -2.65848 | 2.274676 | 4.85E-28 | 3.18E-26 |
| GPRIN1 | 2.75926 | 2.940194 | 1.70E-11 | 2.68E-10 | AKAP12 | -2.65801 | 4.212005 | 1.56E-24 | 8.10E-23 |
| KIF26B | 2.75341 | 2.372044 | 1.15E-10 | 1.64E-09 | TPRG1 | -2.65288 | 0.755408 | 3.21E-23 | 1.53E-21 |
| PTCHD2 | 2.744768 | -0.98023 | 3.66E-05 | 0.000187 | TMEM229A | -2.64856 | -1.75852 | 0.000882 | 0.003106 |
| PYCR1 | 2.740635 | 5.984494 | 3.60E-12 | 6.25E-11 | ADARB1 | -2.64846 | 3.289942 | 3.78E-38 | 4.66E-36 |
| HIST1H2BC | 2.740395 | 4.084131 | 1.28E-08 | 1.33E-07 | EBF2 | -2.64791 | -1.22001 | 5.19E-20 | 2.00E-18 |
| APOA1 | 2.738505 | 1.558852 | 0.002737 | 0.008343 | PHYHIP | -2.64633 | 2.027091 | 9.87E-16 | 2.56E-14 |
| KIF12 | 2.738432 | 1.702246 | 0.000306 | 0.001224 | PDE5A | -2.64091 | 2.454732 | 3.06E-37 | 3.50E-35 |
| CYP11A1 | 2.738202 | 2.564457 | 0.000625 | 0.002297 | RCAN1 | -2.63837 | 4.737312 | 5.32E-40 | 7.43E-38 |
| ZNF488 | 2.735455 | 3.41671 | 3.32E-06 | 2.14E-05 | TMPRSS11D | -2.63774 | 2.532929 | 0.000201 | 0.000846 |
| HIST1H2AG | 2.72721 | 2.253132 | 3.60E-09 | 4.11E-08 | SLMAP | -2.63676 | 4.040515 | 6.33E-79 | 1.55E-75 |
| CNTD2 | 2.726728 | 0.862113 | 3.86E-06 | 2.47E-05 | CADM3 | -2.63124 | 1.955752 | 2.47E-07 | 2.02E-06 |
| LRRC31 | 2.722254 | -1.40368 | 0.005974 | 0.016376 | SYPL2 | -2.62958 | 0.179383 | 1.54E-18 | 5.30E-17 |
| FAM72C | 2.721265 | 0.407371 | 3.62E-11 | 5.48E-10 | DCN | -2.62737 | 6.337164 | 1.10E-18 | 3.82E-17 |
| VEPH1 | 2.717579 | 0.426015 | 1.51E-05 | 8.50E-05 | MAGEE2 | -2.61953 | -3.688 | 0.001628 | 0.005315 |
| DIO1 | 2.716374 | 0.437009 | 0.000573 | 0.00213 | FILIP1L | -2.61304 | 4.504047 | 5.70E-29 | 3.90E-27 |
| SBK1 | 2.715548 | 3.931258 | 3.37E-06 | 2.17E-05 | ALDH1B1 | -2.6115 | 5.992292 | 8.46E-43 | 1.31E-40 |
| NKAIN4 | 2.712985 | -0.3355 | 0.000132 | 0.000586 | FAM180A | -2.61117 | 0.408549 | 2.56E-10 | 3.46E-09 |
| KRT33A | 2.709612 | 2.696411 | 0.016072 | 0.038087 | RNF180 | -2.6098 | 0.472896 | 3.31E-28 | 2.21E-26 |
| TROAP | 2.70497 | 4.581084 | 1.03E-18 | 3.58E-17 | LRCH2 | -2.60935 | 1.215075 | 1.79E-22 | 8.20E-21 |
| DNAJB13 | 2.704164 | 0.443603 | 6.42E-05 | 0.00031 | FBXL7 | -2.60603 | 2.336624 | 1.13E-31 | 9.20E-30 |
| KCNK13 | 2.702695 | 1.749679 | 1.39E-05 | 7.87E-05 | ABCC8 | -2.60564 | -3.34189 | 4.19E-05 | 0.000211 |
| ENSG00000248329 | 2.701012 | -0.91271 | 0.010115 | 0.025728 | KLK11 | -2.60542 | 3.21493 | 2.36E-05 | 0.000126 |
| TSPEAR | 2.699161 | -0.88239 | 0.011466 | 0.028617 | TGM1 | -2.60268 | 4.610061 | 1.92E-09 | 2.28E-08 |
| TBX1 | 2.698584 | 4.768624 | 1.70E-05 | 9.45E-05 | TSPAN18 | -2.60031 | 3.140881 | 7.08E-27 | 4.30E-25 |
| CYP4F8 | 2.695596 | 4.926478 | 0.015654 | 0.037237 | ANO5 | -2.59593 | 0.192632 | 3.23E-14 | 7.09E-13 |
| CTSE | 2.689556 | 6.182055 | 0.006844 | 0.018413 | PTGES3L | -2.59261 | 0.073314 | 2.67E-33 | 2.36E-31 |
| CDH15 | 2.688861 | -1.95575 | 0.000173 | 0.000744 | SNTG2 | -2.58903 | -3.15115 | 2.20E-07 | 1.82E-06 |
| DDN | 2.687212 | -0.4024 | 3.12E-06 | 2.03E-05 | ABCD2 | -2.58592 | -1.38624 | 3.26E-15 | 7.93E-14 |
| CELSR3 | 2.681411 | 2.549307 | 1.86E-10 | 2.56E-09 | GPR15 | -2.58446 | 0.261629 | 2.88E-06 | 1.89E-05 |
| UBE2C | 2.677826 | 7.710241 | 9.96E-18 | 3.14E-16 | KCNQ4 | -2.58312 | 0.879539 | 3.16E-20 | 1.23E-18 |
| CENPA | 2.673539 | 4.208245 | 6.22E-16 | 1.66E-14 | GTF2A1L | -2.58192 | -4.18912 | 0.000561 | 0.00209 |
| ACKR2 | 2.668323 | 2.928212 | 9.12E-05 | 0.000424 | SALL3 | -2.5818 | -2.96855 | 0.002904 | 0.008791 |
| DLX4 | 2.665825 | 1.157778 | 1.24E-05 | 7.10E-05 | KCNH2 | -2.57946 | 2.228311 | 3.58E-11 | 5.43E-10 |
| GPR83 | 2.660339 | -0.69419 | 0.001423 | 0.004733 | COL21A1 | -2.57193 | 0.848189 | 1.65E-15 | 4.18E-14 |
| IFI6 | 2.659613 | 10.13349 | 2.72E-07 | 2.21E-06 | RGN | -2.56981 | 0.7976 | 2.15E-15 | 5.38E-14 |
| GABBR2 | 2.65742 | 3.547917 | 0.003094 | 0.009291 | WFDC1 | -2.5687 | 2.431624 | 1.11E-25 | 6.35E-24 |
| CLEC18A | 2.654015 | -2.17016 | 0.000218 | 0.000907 | RPTN | -2.5672 | 0.375131 | 0.004285 | 0.012302 |
| LIPG | 2.651951 | 2.860071 | 5.29E-07 | 4.07E-06 | STON1 | -2.56657 | 2.276036 | 7.87E-32 | 6.41E-30 |
| NT5DC4 | 2.651572 | -1.7838 | 1.20E-07 | 1.04E-06 | MYO18B | -2.5663 | -0.84076 | 5.33E-09 | 5.95E-08 |
| ETV4 | 2.649439 | 5.121766 | 2.92E-10 | 3.91E-09 | ABCC9 | -2.56502 | 0.646876 | 3.37E-22 | 1.51E-20 |
| SLC15A1 | 2.649195 | 3.518042 | 1.72E-05 | 9.52E-05 | SCUBE1 | -2.56413 | -0.85759 | 7.91E-18 | 2.52E-16 |
| ITIH6 | 2.645462 | -2.06907 | 0.000404 | 0.001567 | TCEAL7 | -2.56278 | 2.332931 | 9.90E-23 | 4.61E-21 |
| CGREF1 | 2.636255 | 2.884225 | 7.64E-06 | 4.57E-05 | SLC8A1 | -2.56263 | 0.978424 | 5.42E-29 | 3.73E-27 |
| ZNF695 | 2.636054 | 0.724186 | 1.68E-12 | 3.02E-11 | GABRA4 | -2.56253 | -6.68836 | 0.0116 | 0.028882 |
| EME1 | 2.635347 | 2.540721 | 7.43E-19 | 2.63E-17 | TCHH | -2.56163 | 0.828662 | 1.51E-09 | 1.83E-08 |
| FNDC1 | 2.63457 | 3.219847 | 0.000197 | 0.000833 | KBTBD13 | -2.55879 | -5.16143 | 0.013739 | 0.033327 |
| MEX3A | 2.631631 | 4.516801 | 8.56E-09 | 9.22E-08 | PLIN4 | -2.55632 | 4.245389 | 5.06E-17 | 1.48E-15 |
| PTH2R | 2.63119 | 1.457711 | 0.013504 | 0.032815 | NTNG1 | -2.55369 | -0.35146 | 1.14E-08 | 1.20E-07 |
| ENSG00000267303 | 2.63093 | -4.05529 | 0.003081 | 0.009257 | EPHA5 | -2.55366 | -4.00563 | 0.000142 | 0.000627 |
| SLC30A3 | 2.627867 | -0.62704 | 0.000824 | 0.002927 | EBF1 | -2.55007 | 1.67742 | 3.18E-27 | 2.00E-25 |
| RAPSN | 2.627191 | 0.822931 | 2.52E-05 | 0.000134 | DLG2 | -2.54816 | -1.56395 | 4.21E-24 | 2.11E-22 |
| HIST1H3A | 2.625236 | 1.176548 | 0.001507 | 0.004971 | RGS5 | -2.54662 | 5.681518 | 1.24E-25 | 6.99E-24 |
| HIST1H2BL | 2.620665 | 1.013627 | 0.000907 | 0.003183 | MUSK | -2.5439 | -2.62181 | 1.42E-09 | 1.72E-08 |
| FAM72B | 2.612757 | 1.257375 | 2.86E-13 | 5.70E-12 | FGF7 | -2.54198 | 2.577972 | 6.80E-12 | 1.14E-10 |
| SNCB | 2.607812 | -2.30968 | 0.021912 | 0.049568 | KIAA2022 | -2.54011 | -2.70268 | 4.87E-08 | 4.57E-07 |
| NUF2 | 2.60668 | 4.462226 | 2.26E-15 | 5.65E-14 | LIPI | -2.53904 | -2.47109 | 5.68E-07 | 4.33E-06 |
| PLA2G2F | 2.601556 | 5.738186 | 0.000957 | 0.003342 | BDKRB1 | -2.53379 | 1.912925 | 2.65E-18 | 8.84E-17 |
| GBP5 | 2.601455 | 4.030868 | 0.000235 | 0.000971 | LAMA2 | -2.53039 | 2.551687 | 4.38E-29 | 3.04E-27 |
| KCNA7 | 2.588331 | -2.72225 | 9.40E-05 | 0.000435 | GHR | -2.53009 | 0.465679 | 3.93E-17 | 1.17E-15 |
| CLEC2L | 2.587765 | 1.278995 | 0.014846 | 0.035565 | SELE | -2.53001 | 3.442498 | 6.81E-11 | 1.00E-09 |
| TLL2 | 2.586041 | -0.19679 | 8.49E-06 | 5.02E-05 | BNC2 | -2.52796 | 0.7116 | 2.39E-16 | 6.64E-15 |
| FOXE3 | 2.585541 | -1.94581 | 0.007246 | 0.019344 | DGKG | -2.5266 | 0.129734 | 9.14E-19 | 3.20E-17 |
| MFI2 | 2.584079 | 3.79098 | 4.29E-06 | 2.72E-05 | SYNC | -2.52652 | 1.028837 | 3.72E-19 | 1.35E-17 |
| ZNF730 | 2.581112 | -0.47215 | 0.001466 | 0.004851 | GCSAML | -2.52319 | -2.53781 | 1.81E-10 | 2.49E-09 |
| SCTR | 2.579743 | -1.41999 | 0.010317 | 0.026183 | TNFRSF13B | -2.52309 | -0.43374 | 7.17E-07 | 5.34E-06 |
| CAMK2N2 | 2.578928 | 1.940347 | 2.27E-06 | 1.53E-05 | FP325317.1 | -2.5219 | -3.95279 | 0.004901 | 0.013818 |
| ANKUB1 | 2.575881 | -3.21064 | 0.013264 | 0.032327 | TACR1 | -2.51861 | -0.40405 | 5.46E-14 | 1.17E-12 |
| UCN2 | 2.57493 | 2.924632 | 1.36E-06 | 9.55E-06 | OLFM4 | -2.51824 | 7.059099 | 0.000122 | 0.000546 |
| HIST2H4A | 2.573017 | -0.75044 | 6.35E-06 | 3.85E-05 | ITGA5 | -2.51451 | 6.267025 | 6.54E-25 | 3.52E-23 |
| LRAT | 2.570556 | -0.07556 | 0.016175 | 0.038282 | SERPINB11 | -2.51235 | 0.363995 | 0.001148 | 0.003917 |
| USH2A | 2.569763 | -2.2543 | 0.002427 | 0.007521 | MYL3 | -2.51161 | -0.9656 | 1.59E-08 | 1.62E-07 |
| METTL7B | 2.566746 | 3.487637 | 1.92E-05 | 0.000105 | VSTM4 | -2.5101 | 2.095234 | 9.27E-30 | 6.90E-28 |
| APOC2 | 2.56495 | -1.7962 | 0.002449 | 0.007577 | BOC | -2.51004 | 2.010509 | 4.39E-16 | 1.19E-14 |
| EPS8L3 | 2.563095 | 2.514851 | 0.007995 | 0.021086 | MFAP5 | -2.50629 | 3.734582 | 1.15E-08 | 1.21E-07 |
| SLCO1A2 | 2.562196 | -0.69083 | 0.00926 | 0.023886 | RGR | -2.50579 | -7.18047 | 0.018473 | 0.042916 |
| SLC22A31 | 2.560264 | -0.86594 | 0.010472 | 0.026518 | FAT4 | -2.50484 | 0.255094 | 1.26E-23 | 6.16E-22 |
| HAP1 | 2.559552 | 1.186429 | 3.08E-05 | 0.00016 | DUSP2 | -2.50267 | 6.307858 | 3.03E-16 | 8.34E-15 |
| C1QTNF6 | 2.556976 | 4.731588 | 1.89E-11 | 2.96E-10 | CHD5 | -2.50253 | -2.61143 | 3.04E-11 | 4.65E-10 |
| SPAG6 | 2.554934 | -1.22514 | 0.00556 | 0.015414 | ATP1B2 | -2.50004 | 0.855404 | 1.13E-25 | 6.42E-24 |
| C4orf48 | 2.54846 | 5.628857 | 5.11E-09 | 5.72E-08 | TNFAIP8L3 | -2.49974 | 2.707478 | 2.37E-18 | 7.93E-17 |
| SEC14L4 | 2.547203 | 0.54057 | 0.00711 | 0.019022 | LGALS7 | -2.49848 | 3.767166 | 0.003981 | 0.011558 |
| MEST | 2.547106 | 5.636092 | 2.96E-07 | 2.38E-06 | C2CD4B | -2.49794 | 2.319968 | 2.78E-15 | 6.90E-14 |
| SLC28A3 | 2.543652 | 2.276881 | 0.000282 | 0.001138 | SYNE1 | -2.49763 | 1.061257 | 1.66E-34 | 1.62E-32 |
| KRTAP4-1 | 2.542343 | 1.089869 | 0.000239 | 0.000985 | NACC2 | -2.49751 | 3.660434 | 1.83E-51 | 5.51E-49 |
| TDRD12 | 2.542281 | 0.1487 | 0.000629 | 0.002307 | TPSD1 | -2.49567 | 1.264265 | 1.01E-06 | 7.28E-06 |
| EMX1 | 2.542238 | -0.31152 | 0.002239 | 0.007009 | TMEM108 | -2.49478 | 0.336657 | 5.43E-12 | 9.20E-11 |
| ISG15 | 2.541634 | 9.417759 | 1.18E-08 | 1.24E-07 | LRRK2 | -2.49418 | 0.103622 | 3.11E-22 | 1.40E-20 |
| HJURP | 2.541531 | 4.323524 | 7.08E-19 | 2.51E-17 | PLCL1 | -2.49206 | 0.094854 | 1.49E-28 | 1.01E-26 |
| SAPCD2 | 2.541409 | 4.718714 | 3.62E-13 | 7.10E-12 | SPATA4 | -2.49064 | -4.00579 | 7.66E-05 | 0.000363 |
| HIST1H1C | 2.538183 | 8.630211 | 3.50E-09 | 4.00E-08 | MT1A | -2.48692 | 3.754583 | 6.66E-07 | 4.99E-06 |
| KCNS1 | 2.536202 | 0.977193 | 8.16E-05 | 0.000384 | SLC8A3 | -2.48585 | -3.26874 | 2.02E-08 | 2.02E-07 |
| COMP | 2.534474 | 6.321466 | 0.001547 | 0.005081 | PRR26 | -2.48158 | -1.81892 | 1.18E-15 | 3.04E-14 |
| CAMK2B | 2.53167 | -0.42316 | 0.000264 | 0.001077 | GNAZ | -2.48136 | 1.724567 | 6.44E-16 | 1.72E-14 |
| TH | 2.531146 | 3.654902 | 0.004697 | 0.013322 | LTBP4 | -2.47669 | 5.213915 | 2.02E-36 | 2.23E-34 |
| LRFN2 | 2.530543 | 0.596773 | 0.005807 | 0.015974 | ANTXR2 | -2.47551 | 4.118702 | 3.88E-32 | 3.22E-30 |
| CTSV | 2.528995 | 3.829162 | 3.77E-05 | 0.000192 | NHSL2 | -2.47184 | -0.32761 | 1.33E-15 | 3.39E-14 |
| FAM171A2 | 2.524232 | 2.445475 | 4.25E-08 | 4.02E-07 | GRIK3 | -2.4718 | -1.67477 | 1.88E-08 | 1.89E-07 |
| MYEOV | 2.521605 | 4.692587 | 0.000766 | 0.002745 | GPR17 | -2.46957 | -1.54605 | 4.90E-12 | 8.35E-11 |
| APOC1 | 2.518785 | 6.806504 | 1.44E-08 | 1.48E-07 | CXCR2 | -2.46938 | 1.454881 | 4.09E-14 | 8.87E-13 |
| MYBL2 | 2.513636 | 6.893664 | 1.38E-11 | 2.23E-10 | LAMC3 | -2.46863 | 2.776682 | 3.37E-24 | 1.71E-22 |
| TFR2 | 2.512678 | 1.760468 | 1.10E-08 | 1.17E-07 | TPSAB1 | -2.46798 | 4.344413 | 1.10E-15 | 2.84E-14 |
| KREMEN2 | 2.506131 | 2.218396 | 7.70E-06 | 4.60E-05 | PRLHR | -2.4679 | -5.91381 | 0.016039 | 0.038025 |
| C21orf58 | 2.504486 | 2.888299 | 6.82E-17 | 1.97E-15 | KCNK2 | -2.46483 | -0.75084 | 3.21E-07 | 2.56E-06 |
| KIF1A | 2.50293 | 0.913554 | 0.015589 | 0.037115 | GPR26 | -2.46209 | -5.95598 | 0.002447 | 0.007572 |
| DNAI1 | 2.499078 | -2.80276 | 0.018254 | 0.042436 | RP11-903H12.5 | -2.45967 | -2.52556 | 1.71E-08 | 1.73E-07 |
| TRIM17 | 2.497924 | 3.026382 | 5.56E-06 | 3.43E-05 | EML1 | -2.45338 | 2.350443 | 4.76E-28 | 3.13E-26 |
| HIST3H2A | 2.497629 | 6.102557 | 1.00E-12 | 1.85E-11 | MYRIP | -2.45169 | -0.81242 | 1.47E-12 | 2.67E-11 |
| UHRF1 | 2.491954 | 4.068126 | 1.50E-18 | 5.18E-17 | CBX7 | -2.4475 | 3.57379 | 2.08E-48 | 4.96E-46 |
| LHFPL5 | 2.48886 | -3.11005 | 0.018133 | 0.042181 | RDH12 | -2.44441 | 1.680635 | 2.23E-10 | 3.04E-09 |
| DOC2A | 2.48625 | -0.00078 | 0.000313 | 0.001249 | TSPAN2 | -2.44235 | 3.603916 | 5.54E-18 | 1.80E-16 |
| ONECUT2 | 2.483775 | 0.812781 | 0.000979 | 0.003405 | IER3 | -2.44049 | 7.832336 | 1.07E-28 | 7.25E-27 |
| CDC20 | 2.482421 | 7.031218 | 2.56E-14 | 5.66E-13 | ARHGEF25 | -2.4399 | 3.983814 | 3.87E-29 | 2.70E-27 |
| DLX1 | 2.480761 | -0.60822 | 0.002189 | 0.006873 | CCDC136 | -2.43858 | 0.936525 | 1.30E-24 | 6.84E-23 |
| GPR78 | 2.47865 | 1.984257 | 0.000399 | 0.001551 | PTGS2 | -2.4364 | 5.665841 | 1.24E-14 | 2.84E-13 |
| CYP27B1 | 2.473721 | 2.291801 | 2.13E-11 | 3.31E-10 | FCN2 | -2.43509 | -3.59439 | 0.011154 | 0.027937 |
| TAT | 2.472969 | -2.25349 | 0.000487 | 0.001843 | CACNA2D1 | -2.43493 | 1.888053 | 7.91E-16 | 2.07E-14 |
| MYADML2 | 2.468591 | -2.72544 | 0.012248 | 0.030271 | SPON1 | -2.43488 | 4.480401 | 9.52E-15 | 2.21E-13 |
| KRT12 | 2.467293 | 0.369663 | 0.006443 | 0.017465 | MYZAP | -2.43333 | 3.690657 | 1.65E-21 | 7.04E-20 |
| LHX2 | 2.467239 | 0.144175 | 0.00737 | 0.019624 | APOLD1 | -2.43204 | 3.785354 | 4.42E-32 | 3.65E-30 |
| TMPRSS3 | 2.466728 | 2.185676 | 0.000235 | 0.000971 | FBLN5 | -2.43181 | 4.042365 | 7.72E-25 | 4.13E-23 |
| OTX1 | 2.462976 | 1.736569 | 7.47E-10 | 9.49E-09 | GFRA2 | -2.43154 | -0.64227 | 1.06E-16 | 3.01E-15 |
| PTPRR | 2.461879 | 3.466306 | 0.000583 | 0.002161 | TMPRSS11E | -2.43065 | 4.824286 | 2.32E-07 | 1.90E-06 |
| ULBP2 | 2.459123 | 4.109086 | 5.87E-09 | 6.51E-08 | MS4A2 | -2.42958 | -0.00668 | 1.09E-14 | 2.52E-13 |
| ENSG00000135747 | 2.452656 | -1.48549 | 1.23E-10 | 1.75E-09 | SRPX | -2.42726 | 4.919463 | 2.02E-14 | 4.54E-13 |
| REG4 | 2.448889 | 1.208482 | 0.00994 | 0.025385 | AQP1 | -2.42536 | 6.447188 | 1.28E-33 | 1.16E-31 |
| TRIM67 | 2.44828 | -3.82613 | 0.000299 | 0.0012 | WISP2 | -2.42532 | 2.522338 | 2.19E-09 | 2.57E-08 |
| DCDC1 | 2.447537 | -3.03907 | 0.000537 | 0.002014 | FNBP1 | -2.41917 | 4.628556 | 9.10E-52 | 2.87E-49 |
| TPX2 | 2.4473 | 6.64268 | 1.08E-14 | 2.50E-13 | SLC51B | -2.41464 | 0.160437 | 2.07E-13 | 4.21E-12 |
| HOXC10 | 2.444596 | 2.600091 | 0.007563 | 0.020074 | DCLK1 | -2.41204 | 0.305104 | 1.33E-11 | 2.14E-10 |
| TUBB3 | 2.440984 | 2.247551 | 7.37E-07 | 5.48E-06 | SOX5 | -2.41083 | -1.26058 | 9.49E-22 | 4.14E-20 |
| PRR5-ARHGAP8 | 2.438637 | -2.21598 | 6.42E-07 | 4.84E-06 | CLDN5 | -2.41043 | 3.869933 | 5.27E-20 | 2.02E-18 |
| MELK | 2.436361 | 4.960577 | 4.56E-13 | 8.82E-12 | RORB | -2.4074 | -1.42091 | 7.01E-09 | 7.64E-08 |
| GTSE1 | 2.432923 | 3.85567 | 6.44E-18 | 2.07E-16 | GSN | -2.40735 | 7.092172 | 4.77E-43 | 7.59E-41 |
| GRAMD2 | 2.427509 | 1.821519 | 0.000106 | 0.000484 | DYNC1I1 | -2.40686 | 2.48453 | 4.43E-17 | 1.31E-15 |
| WNT3 | 2.423693 | 1.864415 | 6.97E-11 | 1.02E-09 | KLF4 | -2.40669 | 5.527584 | 1.12E-27 | 7.18E-26 |
| INHBA | 2.41966 | 4.106291 | 6.37E-06 | 3.87E-05 | CRYM | -2.40414 | 1.923548 | 2.92E-10 | 3.91E-09 |
| VSTM2L | 2.419463 | 3.664531 | 0.001254 | 0.004235 | ITGB3 | -2.40397 | 0.786902 | 2.96E-15 | 7.29E-14 |
| KIF2C | 2.413625 | 5.362423 | 5.23E-18 | 1.70E-16 | RNF112 | -2.40215 | 0.204136 | 2.65E-20 | 1.03E-18 |
| SERPINC1 | 2.411893 | -0.33292 | 0.001578 | 0.005165 | MAPK4 | -2.40185 | 0.535919 | 4.35E-07 | 3.41E-06 |
| EEF1A2 | 2.410458 | 6.636393 | 0.001632 | 0.005324 | PODN | -2.40117 | 4.440149 | 4.04E-16 | 1.10E-14 |
| PFKFB4 | 2.410031 | 3.476522 | 4.99E-15 | 1.20E-13 | STARD9 | -2.40062 | 0.015095 | 1.42E-33 | 1.28E-31 |
| ATP6V0A4 | 2.408587 | 1.765905 | 0.005001 | 0.014074 | SEMA3E | -2.39738 | 0.940931 | 2.16E-11 | 3.35E-10 |
| AURKA | 2.408484 | 5.190765 | 2.84E-18 | 9.42E-17 | MYADM | -2.39287 | 6.480985 | 3.69E-22 | 1.64E-20 |
| GPR158 | 2.408451 | 0.166557 | 0.000688 | 0.002504 | C2orf71 | -2.39071 | -4.78425 | 0.000335 | 0.001326 |
| XRCC2 | 2.408304 | 2.456839 | 2.47E-18 | 8.24E-17 | PTH1R | -2.38777 | 0.742341 | 1.89E-18 | 6.43E-17 |
| SPTA1 | 2.407428 | -2.96611 | 0.019923 | 0.045702 | GPR124 | -2.38757 | 3.951805 | 3.38E-25 | 1.85E-23 |
| NPW | 2.407287 | 0.562849 | 0.000257 | 0.001051 | DPYSL3 | -2.38458 | 5.980603 | 1.67E-17 | 5.14E-16 |
| HIST1H2BD | 2.404444 | 6.28092 | 5.70E-11 | 8.45E-10 | FAM163B | -2.38341 | -1.62267 | 5.71E-05 | 0.000279 |
| PPFIA4 | 2.402946 | 0.391371 | 3.34E-07 | 2.66E-06 | KCNS2 | -2.38302 | -3.04149 | 2.77E-08 | 2.71E-07 |
| HOXC11 | 2.397934 | 1.206881 | 0.002119 | 0.006683 | FHL5 | -2.38105 | 0.527824 | 1.71E-15 | 4.33E-14 |
| PNPLA1 | 2.397052 | 0.283795 | 0.000152 | 0.000663 | HDC | -2.37977 | 0.343209 | 3.81E-14 | 8.31E-13 |
| C1QL4 | 2.396814 | -1.26083 | 0.01477 | 0.035445 | PTGER3 | -2.37882 | 0.529515 | 5.18E-11 | 7.70E-10 |
| KCNK9 | 2.392823 | -0.91508 | 0.002014 | 0.00639 | C15orf59 | -2.36931 | 0.713708 | 1.33E-10 | 1.87E-09 |
| GNG4 | 2.391885 | 2.495508 | 0.001105 | 0.003785 | RASGRP2 | -2.36872 | 1.7853 | 6.00E-18 | 1.94E-16 |
| PABPC1L | 2.389162 | 4.942266 | 1.17E-11 | 1.91E-10 | CPA3 | -2.36529 | 3.933548 | 1.11E-12 | 2.04E-11 |
| COL7A1 | 2.387075 | 6.163864 | 3.17E-07 | 2.54E-06 | RASSF3 | -2.36425 | 5.10188 | 8.13E-54 | 3.06E-51 |
| CDCA8 | 2.38661 | 5.379122 | 1.44E-17 | 4.48E-16 | SEL1L2 | -2.3629 | -4.55216 | 0.000422 | 0.001628 |
| C4orf22 | 2.38618 | -4.01947 | 0.006925 | 0.01859 | PARD3B | -2.36259 | 1.326791 | 5.11E-27 | 3.16E-25 |
| CXCL9 | 2.383308 | 6.158563 | 0.000807 | 0.002872 | LDLRAD2 | -2.36227 | 0.032835 | 6.94E-35 | 7.19E-33 |
| DUSP9 | 2.382601 | 3.524036 | 0.000246 | 0.00101 | VSIG1 | -2.35589 | 1.799984 | 8.28E-10 | 1.04E-08 |
| 3-Sep | 2.376966 | 2.323893 | 3.77E-05 | 0.000192 | ZNF536 | -2.35528 | -1.71832 | 3.55E-06 | 2.28E-05 |
| LBX2 | 2.376891 | 1.3005 | 8.20E-14 | 1.73E-12 | PPAP2B | -2.35518 | 5.041402 | 1.24E-47 | 2.82E-45 |
| TMSB15A | 2.370721 | 3.642608 | 0.000352 | 0.001385 | FAM150B | -2.35425 | -0.70275 | 7.49E-14 | 1.59E-12 |
| POLQ | 2.370533 | 2.237875 | 4.72E-16 | 1.27E-14 | ADCY2 | -2.35302 | -1.66453 | 7.82E-10 | 9.92E-09 |
| IGF2BP2 | 2.368763 | 4.294908 | 7.30E-05 | 0.000348 | CADM2 | -2.35004 | -1.92064 | 3.72E-05 | 0.000189 |
| GP6 | 2.367655 | 0.458314 | 0.001426 | 0.004741 | SOCS3 | -2.3459 | 7.498683 | 1.41E-25 | 7.95E-24 |
| KCNJ6 | 2.367143 | -4.46894 | 0.001129 | 0.003861 | RSPO1 | -2.3448 | -1.55337 | 1.14E-06 | 8.15E-06 |
| TNN | 2.362945 | 0.331839 | 0.004242 | 0.012205 | PRICKLE2 | -2.34415 | 2.350828 | 3.02E-29 | 2.13E-27 |
| CDK1 | 2.361584 | 5.541293 | 1.16E-18 | 4.04E-17 | IL2 | -2.33765 | -3.28756 | 0.008005 | 0.021099 |
| GCKR | 2.359687 | 0.297764 | 0.001648 | 0.005369 | EGR2 | -2.33685 | 3.290996 | 3.79E-17 | 1.13E-15 |
| NPIPB15 | 2.35681 | 3.413632 | 0.000502 | 0.001895 | NTF3 | -2.33551 | 0.198705 | 7.48E-11 | 1.09E-09 |
| CAGE1 | 2.355156 | -2.05147 | 0.002637 | 0.00808 | TRPA1 | -2.33373 | 2.251371 | 1.51E-09 | 1.83E-08 |
| NEURL3 | 2.354443 | 1.553235 | 0.000631 | 0.002316 | PTX3 | -2.33023 | 3.612469 | 8.02E-08 | 7.22E-07 |
| 10-Mar | 2.352455 | -1.24353 | 4.22E-06 | 2.68E-05 | SLC24A3 | -2.32881 | 3.105915 | 1.24E-16 | 3.54E-15 |
| LKAAEAR1 | 2.351529 | -0.48838 | 0.011338 | 0.028349 | PELI2 | -2.32694 | 2.158979 | 3.08E-21 | 1.29E-19 |
| CASP5 | 2.351369 | -0.49751 | 0.003252 | 0.009712 | SAMD4A | -2.32472 | 2.114286 | 1.11E-26 | 6.68E-25 |
| NEK2 | 2.350199 | 4.415722 | 1.81E-14 | 4.09E-13 | GPBAR1 | -2.32193 | 0.880843 | 1.86E-20 | 7.36E-19 |
| SLC10A4 | 2.348698 | 0.828416 | 0.00105 | 0.00362 | ANXA6 | -2.32175 | 5.942701 | 2.84E-21 | 1.20E-19 |
| INHA | 2.347122 | 1.662881 | 0.000121 | 0.000544 | CKB | -2.3211 | 7.049773 | 2.46E-15 | 6.12E-14 |
| IDO1 | 2.344391 | 5.529163 | 0.002122 | 0.006693 | MGARP | -2.31917 | -0.61306 | 9.10E-13 | 1.69E-11 |
| TMEM52 | 2.341852 | 2.21764 | 2.22E-06 | 1.50E-05 | KCNN2 | -2.31906 | -0.04884 | 5.77E-15 | 1.38E-13 |
| KIF4A | 2.340885 | 4.461251 | 1.26E-15 | 3.22E-14 | RAMP1 | -2.31875 | 5.495602 | 4.68E-12 | 8.01E-11 |
| GJB7 | 2.338177 | 1.775367 | 0.000289 | 0.001166 | MEDAG | -2.31802 | 3.817478 | 5.41E-12 | 9.17E-11 |
| DKK2 | 2.336533 | 0.890515 | 4.92E-05 | 0.000244 | PDE4D | -2.31558 | 2.25719 | 1.04E-35 | 1.12E-33 |
| TDO2 | 2.33572 | 1.7811 | 3.68E-07 | 2.91E-06 | PENK | -2.31492 | -1.12727 | 0.000822 | 0.002922 |
| LAMP5 | 2.335545 | 2.43283 | 0.00018 | 0.000768 | MPZ | -2.31465 | 1.867639 | 3.94E-26 | 2.31E-24 |
| TMEM151B | 2.334031 | -2.30432 | 0.002278 | 0.007119 | RBMS3 | -2.31371 | 1.271595 | 2.00E-20 | 7.89E-19 |
| DRC1 | 2.333993 | -1.74849 | 0.001673 | 0.00544 | PTGDS | -2.31353 | 5.89643 | 1.24E-10 | 1.76E-09 |
| RAD54L | 2.332358 | 3.587457 | 1.03E-15 | 2.66E-14 | ITGA9 | -2.31217 | 2.139944 | 3.95E-26 | 2.31E-24 |
| ZNF492 | 2.330616 | -0.6481 | 0.005707 | 0.01575 | DAAM2 | -2.31028 | 1.812627 | 9.85E-23 | 4.60E-21 |
| CPB2 | 2.329267 | -1.53542 | 0.014885 | 0.035645 | FBLN2 | -2.31 | 5.59312 | 6.72E-15 | 1.60E-13 |
| PEX5L | 2.324763 | -2.49882 | 0.008684 | 0.022651 | PRKCB | -2.30928 | 1.675378 | 9.37E-15 | 2.18E-13 |
| VSIG8 | 2.324432 | 1.684775 | 0.004632 | 0.013166 | MEOX1 | -2.30865 | 1.750835 | 3.16E-13 | 6.27E-12 |
| SGOL1 | 2.320944 | 3.015658 | 4.29E-14 | 9.31E-13 | SSTR1 | -2.30768 | -0.40437 | 9.96E-07 | 7.21E-06 |
| IQGAP3 | 2.318639 | 4.384639 | 7.32E-14 | 1.56E-12 | OLFM1 | -2.30748 | 1.478746 | 1.33E-12 | 2.42E-11 |
| C15orf48 | 2.318492 | 6.535343 | 4.05E-05 | 0.000205 | SLITRK3 | -2.3072 | -1.22326 | 0.000956 | 0.003339 |
| KIRREL2 | 2.318181 | -1.23736 | 0.016963 | 0.039867 | KLHL10 | -2.30608 | -2.96727 | 2.86E-10 | 3.83E-09 |
| RTN4R | 2.314711 | 2.780365 | 5.19E-10 | 6.70E-09 | MATN2 | -2.30602 | 4.558096 | 5.60E-16 | 1.50E-14 |
| SLC47A2 | 2.313 | 0.307733 | 0.010732 | 0.027063 | ANGPTL5 | -2.30518 | -2.10288 | 0.003352 | 0.009973 |
| RARRES1 | 2.311947 | 8.058532 | 0.000699 | 0.002535 | SHH | -2.30415 | 4.192141 | 1.57E-05 | 8.80E-05 |
| CELF4 | 2.308972 | -1.19809 | 0.000799 | 0.002846 | PCDH9 | -2.30249 | -2.34456 | 3.40E-08 | 3.27E-07 |
| FMO3 | 2.302377 | 2.57212 | 0.002884 | 0.008732 | CRLF2 | -2.30169 | -2.83087 | 1.61E-08 | 1.64E-07 |
| MYOM3 | 2.299256 | -0.65571 | 2.09E-05 | 0.000113 | TTLL7 | -2.29898 | 1.465261 | 1.20E-19 | 4.53E-18 |
| PLK1 | 2.295143 | 5.120705 | 2.44E-14 | 5.43E-13 | HSPB2 | -2.29396 | 0.963204 | 1.85E-16 | 5.18E-15 |
| HOXC4 | 2.294975 | 2.102157 | 7.15E-05 | 0.000342 | SCN4B | -2.29215 | 0.977942 | 2.75E-19 | 1.01E-17 |
| DRC7 | 2.294519 | -3.16043 | 0.005891 | 0.01617 | FOXD3 | -2.29202 | -1.45258 | 0.000198 | 0.000836 |
| ADM2 | 2.291774 | 2.12638 | 5.55E-07 | 4.25E-06 | TGFB1I1 | -2.28602 | 4.531409 | 7.03E-26 | 4.06E-24 |
| NAT8 | 2.289044 | -0.30754 | 0.01163 | 0.02895 | NCALD | -2.28367 | 2.084494 | 4.50E-24 | 2.24E-22 |
| CCNE1 | 2.288697 | 5.112548 | 1.60E-11 | 2.55E-10 | PABPC5 | -2.28299 | -1.06466 | 1.35E-11 | 2.18E-10 |
| TG | 2.28522 | 1.253016 | 0.003366 | 0.010012 | RSPO3 | -2.28256 | 1.762898 | 3.05E-07 | 2.45E-06 |
| TTK | 2.284831 | 3.830036 | 1.05E-14 | 2.43E-13 | HAAO | -2.27853 | 2.701386 | 3.53E-25 | 1.92E-23 |
| B3GNT4 | 2.282906 | 0.760065 | 1.17E-08 | 1.23E-07 | TP53INP2 | -2.27429 | 5.103224 | 2.59E-34 | 2.46E-32 |
| SYCP2 | 2.278825 | 1.655633 | 3.55E-06 | 2.28E-05 | CLCA4 | -2.27306 | 5.850942 | 3.17E-05 | 0.000164 |
| MAPK8IP2 | 2.278634 | 2.169018 | 1.01E-05 | 5.91E-05 | CPA6 | -2.27102 | 0.211423 | 5.02E-06 | 3.12E-05 |
| TMEM132A | 2.277697 | 5.263432 | 4.40E-14 | 9.53E-13 | MXRA7 | -2.26703 | 4.396508 | 1.98E-37 | 2.28E-35 |
| ZIC2 | 2.277422 | 2.360105 | 0.002116 | 0.006676 | OLFML1 | -2.26589 | 2.348447 | 2.26E-21 | 9.57E-20 |
| MGAM | 2.275516 | -0.21411 | 0.002663 | 0.008146 | A2M | -2.26434 | 8.040885 | 2.73E-21 | 1.15E-19 |
| STRIP2 | 2.274763 | 1.744799 | 5.93E-09 | 6.56E-08 | ARC | -2.26238 | 1.153881 | 4.03E-08 | 3.84E-07 |
| CA2 | 2.274521 | 5.6082 | 0.000167 | 0.000721 | GALR1 | -2.26139 | -6.4182 | 0.002837 | 0.008613 |
| HGD | 2.270241 | -0.46364 | 0.003566 | 0.010535 | ACTA1 | -2.26125 | 1.060345 | 1.19E-14 | 2.73E-13 |
| MAST1 | 2.269208 | 0.637389 | 6.38E-07 | 4.81E-06 | TUSC5 | -2.26099 | -0.78036 | 0.006673 | 0.018012 |
| PCSK9 | 2.268887 | 2.371282 | 0.003388 | 0.010064 | CYBRD1 | -2.25829 | 5.144385 | 3.78E-21 | 1.57E-19 |
| C10orf67 | 2.266221 | -0.70421 | 0.005327 | 0.014856 | CBLN1 | -2.24805 | -1.12811 | 0.000191 | 0.000811 |
| ZWINT | 2.26359 | 6.066178 | 6.93E-18 | 2.22E-16 | SRD5A2 | -2.24447 | 0.081257 | 3.17E-05 | 0.000164 |
| HSD17B1 | 2.262909 | 2.806886 | 2.87E-06 | 1.88E-05 | DCHS1 | -2.2403 | 2.606135 | 3.30E-23 | 1.57E-21 |
| OXTR | 2.262332 | 1.51496 | 0.00013 | 0.000579 | CAP2 | -2.23857 | 3.166309 | 2.11E-21 | 8.97E-20 |
| IYD | 2.259395 | -0.62965 | 0.008846 | 0.023012 | SCEL | -2.23833 | 3.473267 | 6.82E-06 | 4.12E-05 |
| SLX1A | 2.258268 | -3.90784 | 0.02117 | 0.048145 | ATP13A4 | -2.23641 | -0.38803 | 2.09E-07 | 1.73E-06 |
| ASPM | 2.256042 | 3.181221 | 9.24E-12 | 1.52E-10 | DAPL1 | -2.23438 | 3.685235 | 0.000186 | 0.000791 |
| ERVMER34-1 | 2.255596 | 2.778952 | 1.30E-06 | 9.18E-06 | ARMC4 | -2.23385 | -1.22887 | 1.91E-06 | 1.30E-05 |
| RLN2 | 2.252995 | 0.576414 | 0.000334 | 0.001322 | THSD4 | -2.2311 | 2.243903 | 1.05E-24 | 5.57E-23 |
| CLEC12B | 2.250838 | -1.92941 | 0.019647 | 0.045175 | KCNA2 | -2.23071 | -4.0033 | 0.000875 | 0.003081 |
| AURKB | 2.249861 | 5.62809 | 1.79E-13 | 3.67E-12 | CDON | -2.22256 | 0.676895 | 1.10E-15 | 2.84E-14 |
| DNMT3B | 2.24588 | 3.705626 | 1.64E-10 | 2.27E-09 | CLIC4 | -2.22108 | 6.71918 | 3.24E-25 | 1.78E-23 |
| C19orf45 | 2.244443 | 1.564711 | 0.000342 | 0.001351 | PKHD1L1 | -2.21994 | -3.49478 | 1.21E-07 | 1.05E-06 |
| CCDC150 | 2.243892 | 0.170708 | 5.31E-13 | 1.02E-11 | COL4A4 | -2.2175 | 0.670619 | 4.75E-11 | 7.08E-10 |
| PALM3 | 2.243663 | 5.196633 | 0.000574 | 0.002132 | CXCL2 | -2.20714 | 4.397025 | 3.73E-09 | 4.24E-08 |
| FAM111B | 2.239612 | 3.802202 | 1.36E-10 | 1.91E-09 | DUSP5 | -2.20685 | 6.853527 | 2.22E-19 | 8.26E-18 |
| SULT1B1 | 2.236246 | 0.534099 | 0.002455 | 0.00759 | DNAJB4 | -2.20395 | 3.951985 | 8.44E-40 | 1.16E-37 |
| SUN3 | 2.236072 | -1.02881 | 0.002777 | 0.008459 | TPSB2 | -2.20092 | 4.355633 | 9.78E-12 | 1.61E-10 |
| DEPDC1B | 2.234924 | 3.310076 | 8.73E-14 | 1.84E-12 | LGI4 | -2.19699 | 1.788408 | 6.28E-19 | 2.23E-17 |
| ENKUR | 2.234117 | -1.29077 | 2.59E-05 | 0.000137 | RASL11A | -2.19569 | 3.925246 | 1.49E-17 | 4.60E-16 |
| CYP3A7 | 2.233146 | 0.340624 | 0.000538 | 0.002015 | GAS7 | -2.19369 | 2.73561 | 4.12E-15 | 9.95E-14 |
| RNFT2 | 2.233089 | 1.470401 | 9.17E-10 | 1.15E-08 | ALOX15B | -2.19243 | 2.797794 | 2.25E-08 | 2.23E-07 |
| CDC45 | 2.23269 | 4.6107 | 1.97E-14 | 4.44E-13 | CCDC178 | -2.19215 | -1.94463 | 3.08E-07 | 2.47E-06 |
| LCN12 | 2.230245 | 0.659952 | 6.98E-05 | 0.000335 | VSIG10L | -2.19138 | 3.509833 | 1.31E-11 | 2.12E-10 |
| MYO1A | 2.227386 | -0.78703 | 0.003523 | 0.010425 | GSTM5 | -2.18508 | 1.696261 | 2.64E-09 | 3.07E-08 |
| CYP8B1 | 2.226405 | -0.84161 | 0.007535 | 0.020006 | BTG2 | -2.18378 | 8.550526 | 1.91E-20 | 7.54E-19 |
| CHAC1 | 2.225204 | 3.22102 | 2.99E-10 | 3.98E-09 | HIPK4 | -2.18316 | -1.68317 | 1.18E-18 | 4.10E-17 |
| TLE6 | 2.222021 | 2.548318 | 3.33E-05 | 0.000171 | PRSS27 | -2.18272 | 2.725161 | 1.04E-12 | 1.92E-11 |
| GPR19 | 2.219833 | 0.708192 | 5.26E-08 | 4.90E-07 | CNTN4 | -2.18218 | -0.49621 | 1.72E-13 | 3.52E-12 |
| NCAPH | 2.218476 | 4.499117 | 2.91E-14 | 6.43E-13 | PER2 | -2.1809 | 3.15654 | 2.32E-43 | 3.76E-41 |
| E2F7 | 2.218275 | 2.421089 | 1.11E-09 | 1.37E-08 | CXCL12 | -2.18053 | 4.266346 | 1.52E-11 | 2.44E-10 |
| STIL | 2.216689 | 2.853798 | 4.15E-17 | 1.23E-15 | COL4A6 | -2.17872 | 4.001735 | 1.83E-11 | 2.87E-10 |
| CDCA3 | 2.215589 | 3.909427 | 1.22E-15 | 3.13E-14 | MRGPRE | -2.17823 | -3.65006 | 0.010041 | 0.025594 |
| SMKR1 | 2.214307 | 2.135399 | 4.15E-05 | 0.000209 | ITGA1 | -2.17338 | 3.048532 | 6.09E-27 | 3.72E-25 |
| FANCD2OS | 2.212733 | -2.66391 | 0.000758 | 0.002722 | INMT | -2.1729 | 2.170309 | 7.07E-17 | 2.04E-15 |
| VAX2 | 2.21221 | 1.985659 | 0.000149 | 0.000652 | SLC7A3 | -2.17151 | -1.16957 | 0.004478 | 0.012774 |
| CKAP2L | 2.21174 | 3.442596 | 3.68E-12 | 6.37E-11 | SLC25A23 | -2.16781 | 4.710202 | 1.30E-29 | 9.58E-28 |
| MYH7B | 2.211646 | 1.859669 | 0.001313 | 0.004412 | RCVRN | -2.16717 | -3.42241 | 2.38E-05 | 0.000127 |
| MATN3 | 2.203506 | 2.177281 | 1.16E-08 | 1.22E-07 | CSTA | -2.16581 | 7.7166 | 1.24E-10 | 1.76E-09 |
| FAM64A | 2.202443 | 3.939285 | 2.22E-10 | 3.02E-09 | BIN1 | -2.16554 | 3.963175 | 1.52E-17 | 4.69E-16 |
| NAGS | 2.201595 | 2.341194 | 1.44E-11 | 2.32E-10 | NFIX | -2.16269 | 4.479572 | 2.68E-29 | 1.92E-27 |
| RIPPLY3 | 2.199326 | 3.170827 | 1.16E-05 | 6.68E-05 | TGFBR3 | -2.16211 | 4.135236 | 1.52E-18 | 5.23E-17 |
| SHD | 2.19813 | -0.21878 | 0.013993 | 0.033845 | CNNM1 | -2.16178 | -0.52727 | 4.48E-08 | 4.22E-07 |
| SAPCD1 | 2.196227 | 0.528468 | 3.17E-08 | 3.07E-07 | SSC5D | -2.16095 | 2.821431 | 4.64E-12 | 7.96E-11 |
| SLC30A2 | 2.190856 | 4.00805 | 0.005558 | 0.01541 | C1QTNF2 | -2.16071 | 1.042111 | 6.88E-19 | 2.45E-17 |
| IGSF9 | 2.18996 | 4.790286 | 1.37E-07 | 1.18E-06 | KLHL13 | -2.15998 | 1.258864 | 6.17E-12 | 1.04E-10 |
| GBP7 | 2.188707 | -1.54216 | 0.000274 | 0.001109 | RHOB | -2.15974 | 8.476725 | 4.22E-34 | 3.94E-32 |
| STEAP1B | 2.188173 | 1.33139 | 0.004111 | 0.011885 | HTR2A | -2.15871 | -2.27746 | 1.20E-05 | 6.92E-05 |
| RECQL4 | 2.184643 | 5.194642 | 9.79E-16 | 2.55E-14 | LIFR | -2.15799 | 2.172031 | 6.61E-15 | 1.57E-13 |
| SKA3 | 2.18453 | 3.956038 | 8.89E-16 | 2.32E-14 | SLC8A2 | -2.1531 | 0.03538 | 3.08E-09 | 3.55E-08 |
| CDT1 | 2.184285 | 5.133737 | 6.62E-14 | 1.41E-12 | FCER1A | -2.15189 | 2.236044 | 1.20E-08 | 1.26E-07 |
| EXO1 | 2.18121 | 3.268782 | 2.11E-13 | 4.30E-12 | CRISPLD2 | -2.15166 | 4.83378 | 2.66E-17 | 8.04E-16 |
| ZP1 | 2.18043 | 0.295355 | 0.000767 | 0.002746 | ELN | -2.14843 | 4.598999 | 3.94E-11 | 5.93E-10 |
| NEUROD2 | 2.179274 | -3.69555 | 0.02154 | 0.048879 | BPIFB1 | -2.14704 | 4.873288 | 0.001234 | 0.004177 |
| RSAD2 | 2.178338 | 4.281329 | 2.95E-05 | 0.000154 | ABCA9 | -2.14469 | 0.598053 | 3.30E-10 | 4.37E-09 |
| TMEM139 | 2.178164 | 3.953542 | 0.000402 | 0.001559 | JUN | -2.14302 | 7.927863 | 5.88E-27 | 3.61E-25 |
| KLHL35 | 2.177579 | 0.907807 | 1.81E-07 | 1.52E-06 | TMEM220 | -2.1426 | 0.955755 | 1.59E-22 | 7.32E-21 |
| STK31 | 2.177558 | 0.434838 | 4.64E-07 | 3.63E-06 | CIDEC | -2.13777 | 0.87013 | 0.003068 | 0.009222 |
| RHPN1 | 2.17685 | 4.983949 | 8.31E-08 | 7.46E-07 | TGM5 | -2.13726 | 0.674411 | 7.14E-06 | 4.29E-05 |
| HTR2C | 2.17617 | 0.425169 | 0.013296 | 0.032396 | COLEC12 | -2.13575 | 2.738393 | 3.21E-10 | 4.26E-09 |
| PAQR4 | 2.176108 | 4.210435 | 2.45E-13 | 4.93E-12 | TSLP | -2.13388 | 0.675931 | 2.55E-13 | 5.13E-12 |
| CASKIN1 | 2.172816 | -0.76679 | 0.000367 | 0.001437 | TUB | -2.13273 | 1.399966 | 2.45E-14 | 5.46E-13 |
| HRASLS2 | 2.172779 | 3.974106 | 0.009642 | 0.024723 | ANKRD29 | -2.12628 | 0.705435 | 1.43E-09 | 1.74E-08 |
| CNIH2 | 2.172525 | 2.240722 | 2.67E-06 | 1.77E-05 | KRT13 | -2.12465 | 10.85757 | 2.40E-05 | 0.000128 |
| CDHR5 | 2.171689 | 0.516905 | 0.003801 | 0.011128 | LEPR | -2.12248 | 1.232113 | 2.91E-22 | 1.31E-20 |
| C16orf59 | 2.170622 | 3.517503 | 1.49E-14 | 3.38E-13 | ITPKB | -2.12209 | 4.267808 | 1.72E-58 | 9.90E-56 |
| CDC25C | 2.168435 | 3.016369 | 3.88E-14 | 8.44E-13 | HPGDS | -2.12043 | 0.785334 | 3.08E-15 | 7.57E-14 |
| DNASE1L2 | 2.164946 | 1.256372 | 5.96E-08 | 5.50E-07 | C16orf45 | -2.11949 | 3.652037 | 1.48E-19 | 5.54E-18 |
| C19orf84 | 2.164305 | -1.13582 | 0.006411 | 0.017391 | NCS1 | -2.11825 | 5.071807 | 2.66E-30 | 2.01E-28 |
| PLAU | 2.161994 | 7.805147 | 4.73E-07 | 3.69E-06 | MROH7-TTC4 | -2.11575 | -4.82875 | 0.000255 | 0.001044 |
| TRIP13 | 2.161698 | 4.837274 | 1.97E-11 | 3.08E-10 | BDKRB2 | -2.11534 | 3.180557 | 2.29E-19 | 8.48E-18 |
| IL31RA | 2.160678 | -1.04282 | 0.019371 | 0.044615 | FGFR1 | -2.11213 | 3.698467 | 4.33E-13 | 8.39E-12 |
| SLAMF9 | 2.160495 | 0.838399 | 0.00231 | 0.007199 | WWTR1 | -2.11184 | 4.470062 | 2.93E-35 | 3.11E-33 |
| PDZD3 | 2.160332 | 0.791986 | 0.014472 | 0.03481 | GCNT3 | -2.11077 | 1.876659 | 8.12E-06 | 4.82E-05 |
| POU5F1 | 2.158827 | 3.842464 | 0.000103 | 0.000471 | KIAA1462 | -2.1094 | 3.525861 | 2.56E-19 | 9.45E-18 |
| IFI27 | 2.158781 | 8.094446 | 4.01E-05 | 0.000203 | PPM1L | -2.10838 | 2.766128 | 1.18E-22 | 5.44E-21 |
| DNAAF3 | 2.158261 | 0.134343 | 5.17E-05 | 0.000255 | GYPC | -2.10752 | 3.948544 | 1.30E-14 | 2.98E-13 |
| ORC1 | 2.158054 | 3.463268 | 6.52E-14 | 1.39E-12 | ACSM5 | -2.10283 | -2.00513 | 5.49E-09 | 6.11E-08 |
| ORC6 | 2.157799 | 3.562609 | 7.21E-15 | 1.70E-13 | CSGALNACT1 | -2.10036 | 2.633638 | 3.40E-29 | 2.39E-27 |
| HAPLN1 | 2.15524 | 0.934592 | 0.000863 | 0.003047 | HSPG2 | -2.09702 | 5.219757 | 2.59E-24 | 1.33E-22 |
| S1PR5 | 2.154661 | 4.241376 | 2.47E-06 | 1.64E-05 | STK32B | -2.09674 | -0.13259 | 1.13E-13 | 2.35E-12 |
| FAM72A | 2.152617 | 0.538551 | 9.74E-13 | 1.80E-11 | PKIG | -2.09672 | 5.821701 | 1.55E-29 | 1.13E-27 |
| SPAG5 | 2.150652 | 4.924584 | 2.41E-16 | 6.70E-15 | SETBP1 | -2.0966 | 1.623576 | 3.38E-16 | 9.26E-15 |
| CBX2 | 2.150129 | 3.861269 | 7.00E-07 | 5.23E-06 | ENPP3 | -2.09515 | -2.29469 | 2.09E-08 | 2.09E-07 |
| GABRQ | 2.149973 | 1.543232 | 0.01855 | 0.043073 | TSHZ3 | -2.09333 | 2.625527 | 2.58E-15 | 6.39E-14 |
| MCM10 | 2.149534 | 3.233431 | 1.47E-11 | 2.35E-10 | RGAG4 | -2.09315 | 1.831944 | 9.10E-14 | 1.91E-12 |
| PODXL2 | 2.149311 | 6.260403 | 3.51E-07 | 2.78E-06 | FAXC | -2.09264 | 0.424343 | 9.95E-14 | 2.08E-12 |
| APLN | 2.146748 | 3.164618 | 5.54E-06 | 3.41E-05 | CXCL13 | -2.09247 | 6.074614 | 6.10E-06 | 3.72E-05 |
| ASF1B | 2.144291 | 5.71276 | 5.21E-16 | 1.40E-14 | CSDC2 | -2.09221 | 2.180895 | 3.17E-08 | 3.06E-07 |
| SKA1 | 2.14315 | 3.919042 | 1.56E-13 | 3.22E-12 | HDAC4 | -2.08836 | 1.735504 | 1.75E-39 | 2.31E-37 |
| HPDL | 2.141493 | 2.824377 | 0.000154 | 0.000668 | MROH2B | -2.0875 | -6.46124 | 0.012628 | 0.031055 |
| HES6 | 2.136118 | 4.463756 | 3.87E-05 | 0.000197 | COL6A5 | -2.08727 | -0.9772 | 6.33E-06 | 3.84E-05 |
| GLRA3 | 2.135975 | -2.15837 | 0.007095 | 0.01899 | CREB5 | -2.08489 | 0.402372 | 9.71E-12 | 1.60E-10 |
| RDH16 | 2.135694 | 1.536549 | 2.89E-05 | 0.000151 | ITM2A | -2.08309 | 4.460046 | 1.75E-10 | 2.41E-09 |
| TUBB8 | 2.135139 | -0.95568 | 0.000642 | 0.002351 | PER1 | -2.08119 | 4.665516 | 1.02E-24 | 5.43E-23 |
| C6orf222 | 2.134466 | -0.45273 | 0.017095 | 0.040133 | ILK | -2.08115 | 3.208514 | 9.94E-39 | 1.26E-36 |
| MESP1 | 2.133732 | 2.857511 | 0.001132 | 0.003871 | TNS2 | -2.08056 | 4.025015 | 3.08E-33 | 2.71E-31 |
| FANCA | 2.133049 | 2.870054 | 7.04E-17 | 2.03E-15 | DMPK | -2.07985 | 5.00539 | 1.46E-42 | 2.26E-40 |
| C9orf173 | 2.13289 | -0.88418 | 0.000239 | 0.000985 | ENSG00000263264 | -2.07979 | -0.44103 | 8.50E-10 | 1.07E-08 |
| FOXM1 | 2.132264 | 5.295536 | 2.09E-11 | 3.26E-10 | APBB1 | -2.07792 | 3.093758 | 2.02E-17 | 6.18E-16 |
| KIF18A | 2.131962 | 2.620712 | 2.79E-12 | 4.90E-11 | LMCD1 | -2.07742 | 3.733315 | 7.20E-16 | 1.90E-14 |
| AIFM3 | 2.131317 | 2.954445 | 5.97E-05 | 0.000291 | VSX1 | -2.07706 | -4.35575 | 0.004372 | 0.012519 |
| FAM132A | 2.130014 | 2.159853 | 1.82E-05 | 0.0001 | CKM | -2.07705 | 0.993915 | 1.49E-09 | 1.80E-08 |
| INA | 2.129062 | 5.449599 | 0.002488 | 0.007686 | ZFHX4 | -2.07682 | 0.60912 | 2.18E-09 | 2.57E-08 |
| SIM2 | 2.128807 | 2.64896 | 3.30E-08 | 3.18E-07 | JAZF1 | -2.07595 | 3.083338 | 2.75E-25 | 1.52E-23 |
| LRRIQ4 | 2.122958 | 0.191789 | 0.001636 | 0.005335 | RNF122 | -2.07573 | 4.243088 | 3.29E-35 | 3.48E-33 |
| CDCA2 | 2.119177 | 3.08723 | 4.80E-12 | 8.19E-11 | RECK | -2.07026 | 1.841085 | 1.24E-21 | 5.35E-20 |
| PRSS8 | 2.119085 | 6.658787 | 2.90E-06 | 1.90E-05 | CD34 | -2.06991 | 3.936331 | 9.17E-30 | 6.85E-28 |
| RLN1 | 2.119042 | -0.15763 | 0.002212 | 0.006934 | KLHL30 | -2.06807 | -0.24217 | 3.67E-12 | 6.37E-11 |
| ENSG00000281370 | 2.1186 | 1.499622 | 0.018813 | 0.043601 | NRXN2 | -2.06702 | -0.11188 | 1.07E-10 | 1.53E-09 |
| GALR2 | 2.118573 | 1.232174 | 0.000788 | 0.002814 | ARID5A | -2.06633 | 4.786997 | 2.95E-33 | 2.60E-31 |
| PCP2 | 2.114834 | 2.612885 | 4.23E-05 | 0.000213 | ENSG00000146197 | -2.06538 | 1.488704 | 1.67E-09 | 2.01E-08 |
| UPK2 | 2.114182 | 10.01058 | 0.006648 | 0.017954 | MEOX2 | -2.06519 | 1.063477 | 5.71E-06 | 3.50E-05 |
| FABP6 | 2.114122 | 5.295018 | 0.001699 | 0.005512 | KLRB1 | -2.0651 | 2.149954 | 3.94E-14 | 8.55E-13 |
| HS3ST3A1 | 2.113081 | 0.946596 | 0.012132 | 0.030042 | HPD | -2.06131 | 0.642672 | 2.32E-11 | 3.58E-10 |
| IER5L | 2.112764 | 5.090684 | 2.16E-13 | 4.37E-12 | WDR49 | -2.06096 | -3.52374 | 0.000246 | 0.001012 |
| CFAP74 | 2.112449 | -2.18356 | 0.000216 | 0.000901 | PDE4B | -2.05937 | 2.027886 | 1.17E-14 | 2.69E-13 |
| RAB40AL | 2.110608 | -3.51109 | 0.008291 | 0.021768 | PITX2 | -2.05843 | 2.134438 | 2.29E-12 | 4.07E-11 |
| LHB | 2.110255 | 1.429546 | 4.15E-05 | 0.000209 | CCDC141 | -2.05768 | -3.59671 | 1.39E-08 | 1.44E-07 |
| KLC3 | 2.109557 | 3.238288 | 3.68E-05 | 0.000187 | CYTL1 | -2.05738 | 2.493298 | 6.07E-09 | 6.68E-08 |
| MTFR2 | 2.109057 | 3.174901 | 2.27E-15 | 5.67E-14 | TTLL2 | -2.05575 | -4.85219 | 0.013916 | 0.033666 |
| CNGB3 | 2.108338 | -0.65279 | 3.81E-06 | 2.44E-05 | MLIP | -2.05434 | -2.49902 | 3.22E-05 | 0.000167 |
| CLEC5A | 2.106923 | 1.238301 | 1.91E-05 | 0.000104 | ZEB2 | -2.05288 | 1.714376 | 3.46E-19 | 1.26E-17 |
| ITIH2 | 2.103462 | -0.19987 | 0.003987 | 0.011573 | ENSG00000274322 | -2.05146 | -0.22082 | 2.36E-12 | 4.17E-11 |
| SPEF1 | 2.103022 | -0.33459 | 1.16E-05 | 6.68E-05 | RND1 | -2.04994 | 2.380306 | 5.06E-16 | 1.36E-14 |
| ELOVL3 | 2.102749 | 1.038857 | 0.000274 | 0.001109 | STXBP6 | -2.04864 | 1.066317 | 1.29E-07 | 1.11E-06 |
| HPN | 2.102678 | 0.520818 | 0.005302 | 0.014801 | TLN1 | -2.04821 | 6.734823 | 1.58E-47 | 3.51E-45 |
| CPNE4 | 2.10229 | -0.00863 | 0.007855 | 0.020764 | DENND2A | -2.04776 | 2.504935 | 1.61E-18 | 5.49E-17 |
| MTL5 | 2.101433 | 1.71359 | 7.89E-08 | 7.13E-07 | ASXL3 | -2.04713 | -1.65798 | 2.97E-07 | 2.39E-06 |
| KIFC1 | 2.100816 | 5.524991 | 5.73E-14 | 1.23E-12 | NAP1L2 | -2.04673 | 1.528003 | 3.02E-11 | 4.63E-10 |
| CCNB2 | 2.099676 | 5.572058 | 3.13E-14 | 6.89E-13 | HSPA12A | -2.04641 | 1.091428 | 2.35E-15 | 5.86E-14 |
| SAA1 | 2.099264 | 7.15941 | 0.01813 | 0.042179 | PRSS35 | -2.04602 | -0.38403 | 4.49E-07 | 3.51E-06 |
| AUNIP | 2.097379 | 3.242513 | 1.48E-13 | 3.06E-12 | GPER1 | -2.04505 | 0.986598 | 5.80E-16 | 1.55E-14 |
| TOP2A | 2.091434 | 6.435544 | 8.67E-12 | 1.43E-10 | EDNRA | -2.04387 | 3.257804 | 2.97E-17 | 8.90E-16 |
| GJA3 | 2.089017 | -0.10116 | 0.002949 | 0.008905 | PCDH18 | -2.0436 | 2.12091 | 1.02E-17 | 3.22E-16 |
| MCIDAS | 2.088477 | 1.314278 | 8.96E-06 | 5.28E-05 | MTURN | -2.04162 | 3.885669 | 9.75E-33 | 8.30E-31 |
| DLGAP5 | 2.084437 | 4.503162 | 1.82E-11 | 2.86E-10 | MITF | -2.03314 | 1.778858 | 5.21E-18 | 1.70E-16 |
| CHRNG | 2.083918 | -2.71758 | 0.003425 | 0.010155 | HEPH | -2.03213 | 2.859718 | 1.68E-12 | 3.02E-11 |
| CDC6 | 2.082101 | 4.577049 | 1.91E-15 | 4.80E-14 | GRIK5 | -2.03181 | 0.575847 | 3.45E-10 | 4.56E-09 |
| SMTNL1 | 2.080083 | 0.531628 | 9.05E-05 | 0.000422 | CRB2 | -2.02913 | -2.99772 | 1.90E-06 | 1.30E-05 |
| HMMR | 2.078417 | 4.033099 | 5.06E-12 | 8.59E-11 | KCNQ5 | -2.02693 | -0.71351 | 8.38E-05 | 0.000394 |
| SMCO2 | 2.078415 | 0.253611 | 4.79E-06 | 3.00E-05 | MUSTN1 | -2.02659 | -1.87253 | 7.19E-06 | 4.32E-05 |
| SYT5 | 2.077076 | -0.45928 | 0.012137 | 0.03005 | STAC | -2.02309 | 0.6831 | 5.00E-08 | 4.67E-07 |
| TSACC | 2.076764 | 0.9916 | 3.78E-11 | 5.71E-10 | FYCO1 | -2.02294 | 4.277819 | 5.32E-43 | 8.39E-41 |
| FZD2 | 2.076605 | 4.142308 | 1.66E-09 | 1.99E-08 | ATP2B4 | -2.02191 | 5.525147 | 1.34E-24 | 7.05E-23 |
| HYAL4 | 2.076402 | -0.77973 | 0.015872 | 0.037681 | MAFF | -2.01923 | 5.329239 | 1.45E-26 | 8.72E-25 |
| NCAPG | 2.076 | 3.97375 | 2.04E-12 | 3.64E-11 | C8orf4 | -2.01875 | 7.447316 | 1.75E-11 | 2.77E-10 |
| TK1 | 2.075227 | 7.379957 | 3.91E-13 | 7.61E-12 | FZD7 | -2.01709 | 4.454369 | 1.65E-23 | 7.96E-22 |
| MYLK4 | 2.074519 | 1.866217 | 0.000885 | 0.003114 | PDGFRA | -2.01588 | 3.198391 | 3.88E-14 | 8.44E-13 |
| GPAT2 | 2.074283 | 2.382559 | 0.001485 | 0.004906 | NAP1L3 | -2.01209 | 1.145252 | 3.72E-10 | 4.89E-09 |
| FBXW12 | 2.073566 | -1.41838 | 0.000694 | 0.002521 | HSPA2 | -2.0112 | 3.851963 | 3.07E-15 | 7.56E-14 |
| CABP4 | 2.072634 | 2.074435 | 2.66E-05 | 0.00014 | FGF13 | -2.01039 | -1.5237 | 1.77E-11 | 2.79E-10 |
| C20orf144 | 2.071495 | -0.897 | 8.16E-13 | 1.53E-11 | NFIA | -2.00972 | 3.410212 | 2.21E-25 | 1.23E-23 |
| SLC39A4 | 2.070445 | 5.007585 | 1.34E-11 | 2.17E-10 | GABRA2 | -2.00799 | -4.37762 | 0.004117 | 0.0119 |
| GINS1 | 2.070067 | 4.386617 | 7.10E-16 | 1.88E-14 | ARID5B | -2.00678 | 4.729559 | 1.08E-34 | 1.09E-32 |
| HSD17B3 | 2.068258 | 1.086551 | 0.000893 | 0.003139 | PTGES3L-AARSD1 | -2.00645 | -2.25982 | 1.95E-07 | 1.62E-06 |
| HIST1H4I | 2.065235 | 4.925891 | 1.95E-09 | 2.31E-08 | PDGFD | -2.0064 | 2.808239 | 2.98E-16 | 8.21E-15 |
| GRIN1 | 2.064463 | -1.29241 | 0.000107 | 0.000487 | SNED1 | -2.00201 | 0.74743 | 9.96E-16 | 2.58E-14 |
| BUB1B | 2.059771 | 4.09536 | 3.63E-13 | 7.10E-12 |  |  |  |  |  |
| CCDC114 | 2.059329 | -0.02993 | 9.22E-06 | 5.43E-05 |  |  |  |  |  |
| CCNE2 | 2.058126 | 2.132598 | 1.42E-09 | 1.73E-08 |  |  |  |  |  |
| BRIP1 | 2.058098 | 2.261826 | 1.05E-12 | 1.94E-11 |  |  |  |  |  |
| CHI3L1 | 2.055099 | 6.560931 | 0.005137 | 0.014392 |  |  |  |  |  |
| SLC12A8 | 2.05393 | 3.54069 | 1.20E-09 | 1.47E-08 |  |  |  |  |  |
| PDZD7 | 2.049149 | -0.08921 | 6.32E-07 | 4.77E-06 |  |  |  |  |  |
| SHOX2 | 2.047019 | 0.731297 | 0.001442 | 0.00479 |  |  |  |  |  |
| LYPD1 | 2.045662 | 1.686794 | 7.84E-05 | 0.00037 |  |  |  |  |  |
| KIF20A | 2.044828 | 4.985076 | 4.52E-12 | 7.75E-11 |  |  |  |  |  |
| MYCN | 2.042154 | 3.555995 | 0.004439 | 0.012679 |  |  |  |  |  |
| SYNE4 | 2.042151 | 4.326544 | 4.84E-06 | 3.02E-05 |  |  |  |  |  |
| KIF15 | 2.04081 | 3.267253 | 6.69E-13 | 1.27E-11 |  |  |  |  |  |
| KIF14 | 2.038244 | 2.59599 | 2.36E-10 | 3.20E-09 |  |  |  |  |  |
| DLX3 | 2.036031 | 3.031407 | 0.00198 | 0.006294 |  |  |  |  |  |
| DNAJC5B | 2.035237 | 0.144395 | 0.007338 | 0.01955 |  |  |  |  |  |
| HMGB3 | 2.033161 | 6.365364 | 1.17E-17 | 3.67E-16 |  |  |  |  |  |
| CCNB1 | 2.033125 | 6.500943 | 2.95E-13 | 5.85E-12 |  |  |  |  |  |
| GALNT6 | 2.029913 | 3.705823 | 1.82E-07 | 1.52E-06 |  |  |  |  |  |
| C17orf47 | 2.029473 | -3.0849 | 0.014454 | 0.034789 |  |  |  |  |  |
| ARID3C | 2.027495 | -1.88129 | 0.003611 | 0.010654 |  |  |  |  |  |
| SPATA17 | 2.027126 | 0.685536 | 2.35E-07 | 1.93E-06 |  |  |  |  |  |
| ERCC6L | 2.026366 | 2.478743 | 1.64E-11 | 2.61E-10 |  |  |  |  |  |
| MKI67 | 2.026322 | 4.975826 | 6.09E-12 | 1.02E-10 |  |  |  |  |  |
| EXD1 | 2.025877 | -4.10872 | 0.007193 | 0.019218 |  |  |  |  |  |
| PRR11 | 2.023788 | 4.420949 | 3.80E-12 | 6.57E-11 |  |  |  |  |  |
| HOXB5 | 2.021183 | 3.867278 | 8.87E-05 | 0.000414 |  |  |  |  |  |
| SUCNR1 | 2.015513 | 0.474719 | 0.000625 | 0.002297 |  |  |  |  |  |
| TREM2 | 2.014032 | 4.581132 | 3.25E-06 | 2.10E-05 |  |  |  |  |  |
| HIST1H2BK | 2.012908 | 7.739827 | 2.53E-08 | 2.50E-07 |  |  |  |  |  |
| MYB | 2.012493 | 2.240959 | 9.84E-06 | 5.77E-05 |  |  |  |  |  |
| LRRC19 | 2.011623 | -1.83199 | 0.01436 | 0.034605 |  |  |  |  |  |
| FAM227A | 2.005365 | 0.020473 | 3.13E-08 | 3.03E-07 |  |  |  |  |  |
| TDRD5 | 2.004726 | 1.800477 | 0.004233 | 0.012183 |  |  |  |  |  |
| CR1L | 2.003745 | -1.85608 | 0.007362 | 0.019608 |  |  |  |  |  |
| CHST8 | 2.000953 | 1.359837 | 0.02132 | 0.04846 |  |  |  |  |  |
| OIP5 | 2.000487 | 3.501577 | 1.25E-12 | 2.28E-11 |  |  |  |  |  |
