# Supplementary material for: Identification of Hub Genes Associated With Progression and Prognosis in Patients With Bladder Cancer
Source: Front Genet. 2019 May 7;10:408. doi: 10.3389/fgene.2019.00408 (PMC6513982; doi:10.3389/fgene.2019.00408)
Supplement: TABLE S2 — Common genes in MeanDecreaseAccuracy and MeanDecreaseGini ranked top 50. [file Table_2.DOC]

**Table S2：Common genes in MeanDecreaseAccuracy and MeanDecreaseGini ranked top 50.**

| **gene symbol** | **MeanDecreaseAccuracy** | **MeanDecreaseGini** | **common genes** |
| --- | --- | --- | --- |
| BUB1B | 5.42015649 | 2.218551 | HIST1H2AD |
| HDAC4 | 5.39329299 | 2.8928861 | HDAC4 |
| ORC1 | 4.70222833 | 1.1326902 | CDK1 |
| CCNB1 | 4.53661299 | 1.2297698 | SHH |
| PLK1 | 4.24683498 | 1.5390343 | HIST1H3B |
| RAD54L | 3.88545787 | 1.5404275 | DCN |
| NCAPG | 3.73355857 | 1.0090604 | ACTBL2 |
| SHH | 3.71604384 | 1.764963 | CCL2 |
| HIST2H4A | 3.67221389 | 1.2425146 | ACTN2 |
| MMP9 | 3.65088358 | 2.1150884 | KIF15 |
| CENPA | 3.48972171 | 0.915656 | ZWINT |
| HIST1H3D | 3.34596821 | 1.592542 | PLK1 |
| TPX2 | 3.32004364 | 1.0081499 | HIST1H3A |
| TRIP13 | 3.28713547 | 1.150447 | CCNB1 |
| HIST1H2AD | 3.27074022 | 1.346447 | BUB1B |
| HIST1H2BC | 3.21740413 | 1.5302345 | HIST2H4A |
| CDC45 | 3.19523663 | 0.8528908 | ISG15 |
| HIST1H3A | 3.15560036 | 1.7116598 | HIST1H2BD |
| KIF15 | 3.13540888 | 1.2630885 | HIST1H2BC |
| SAA1 | 3.05756012 | 1.0803008 | FOXM1 |
| HIST1H2BD | 3.03965066 | 1.7088239 | OIP5 |
| CCNB2 | 3.0345991 | 1.0496466 | CSF2 |
| OIP5 | 3.00231426 | 1.3599848 | HIST1H3D |
| ASPM | 2.96917386 | 1.0435929 | MMP9 |
| CDCA8 | 2.95311022 | 0.9992309 | RAD54L |
| MCM10 | 2.78504385 | 0.9812561 |  |
| HIST1H4I | 2.65477342 | 1.039364 |  |
| CSF2 | 2.64935226 | 1.6622473 |  |
| CDK1 | 2.6061986 | 1.3635384 |  |
| HIST1H3B | 2.58151432 | 1.5402081 |  |
| HIST1H3I | 2.56849179 | 0.6357823 |  |
| DLGAP5 | 2.55306144 | 1.0708839 |  |
| TTK | 2.5267359 | 1.1249197 |  |
| DCN | 2.50632445 | 1.9506307 |  |
| ACTC1 | 2.46751046 | 1.1075776 |  |
| UBE2C | 2.45270068 | 0.9483931 |  |
| KIF4A | 2.42489154 | 1.0482217 |  |
| ACTN2 | 2.39731787 | 1.7371956 |  |
| AURKB | 2.34071145 | 1.1591704 |  |
| ZWINT | 2.33292385 | 2.2079596 |  |
| AURKA | 2.31634571 | 0.9246175 |  |
| NUF2 | 2.31539917 | 0.7784414 |  |
| ISG15 | 2.21528702 | 1.3536179 |  |
| CCL2 | 2.17003597 | 1.2818076 |  |
| HIST1H2BJ | 2.13119271 | 1.0095939 |  |
| HIST1H2BK | 2.11535789 | 1.0967047 |  |
| CACNA1C | 2.0999304 | 1.1325798 |  |
| KIF20A | 2.08239116 | 0.9006232 |  |
| FOXM1 | 2.06428219 | 1.4025593 |  |
| ACTBL2 | 1.98336221 | 1.2652787 |  |
| CXCL12 | 1.96372878 | 2.1750823 |  |
| CXCL10 | 1.95102981 | 1.311328 |  |
| JUN | 1.9300625 | 1.3589071 |  |
| HIST1H4B | 1.91585772 | 0.7946731 |  |
| HJURP | 1.89288545 | 1.2800821 |  |
| KIF2C | 1.88670397 | 1.0337365 |  |
| CDC25C | 1.86733221 | 1.2897427 |  |
| IL13 | 1.8570101 | 1.3312404 |  |
| ACTG2 | 1.81658691 | 0.9976369 |  |
| NCAPH | 1.80490281 | 1.4823033 |  |
| CDC20 | 1.80255899 | 0.7309806 |  |
| HIST1H2BL | 1.80188159 | 1.1100773 |  |
| THBS1 | 1.79979795 | 1.647653 |  |
| GPR17 | 1.61701051 | 0.8138992 |  |
| TOP2A | 1.46380038 | 1.0928431 |  |
| EXO1 | 1.4562416 | 0.9171955 |  |
| KIF14 | 1.45393516 | 0.9457197 |  |
| GNG4 | 1.45159018 | 1.5911865 |  |
| KIF18A | 1.43782435 | 1.1106125 |  |
| NEK2 | 1.40118732 | 1.4563796 |  |
| HIST1H3G | 1.39397291 | 0.8429983 |  |
| CXCL2 | 1.3740305 | 1.2080357 |  |
| PTGS2 | 1.32499591 | 1.3164962 |  |
| CDT1 | 1.31686986 | 0.7782801 |  |
| HIST3H2BB | 1.24381815 | 1.469924 |  |
| HMMR | 1.21796158 | 0.8283146 |  |
| PRKG1 | 1.20187631 | 1.5729221 |  |
| HIST1H2BO | 1.16968801 | 0.9289394 |  |
| CALB1 | 1.14033195 | 1.201691 |  |
| POLQ | 1.07240626 | 1.0783258 |  |
| HIST1H2BF | 1.07164035 | 1.0496383 |  |
| ADCY5 | 1.06755875 | 1.2003409 |  |
| HIST1H2BI | 1.00130014 | 0.9364654 |  |
| CXCL9 | 1.00045842 | 1.0993288 |  |
| MELK | 0.99367207 | 0.9108399 |  |
| F2 | 0.9845384 | 1.6956768 |  |
| GNG7 | 0.90406637 | 0.8795077 |  |
| ACTA2 | 0.83263358 | 1.5104888 |  |
| IL6 | 0.79600862 | 0.9255747 |  |
| FAM64A | 0.78165234 | 1.1396239 |  |
| MKI67 | 0.78097362 | 1.1734593 |  |
| AGTR1 | 0.75415729 | 1.2687495 |  |
| HIST1H4D | 0.74406926 | 1.0228978 |  |
| HIST2H3D | 0.71001748 | 1.219432 |  |
| BDKRB1 | 0.70204068 | 1.2546358 |  |
| ALB | 0.5533793 | 0.7600784 |  |
| HIST1H3H | 0.53344349 | 1.240997 |  |
| HIST1H3J | 0.48389778 | 0.7411279 |  |
| HIST1H3F | 0.47616029 | 0.8761491 |  |
| ESR1 | 0.44972716 | 1.4045804 |  |
| HIST1H2BM | 0.43072316 | 0.5257158 |  |
| GNAO1 | 0.39794301 | 1.2742623 |  |
| MYOD1 | 0.27254782 | 0.9652076 |  |
| LRRK2 | 0.24888164 | 1.1002432 |  |
| HIST1H3C | 0.24307709 | 1.105458 |  |
| GAL | 0.22654901 | 1.2724691 |  |
| CDKN2A | 0.20812392 | 0.961272 |  |
| SPAG5 | 0.20564128 | 1.1415845 |  |
| FGF2 | 0.0488529 | 1.1560366 |  |
| IL2 | 0.01278681 | 0.5038082 |  |
| BDKRB2 | -0.01543308 | 1.0332778 |  |
| CDC6 | -0.18877373 | 0.9757944 |  |
| ADCY2 | -0.20259563 | 1.0438146 |  |
| ASF1B | -0.21367217 | 1.1718763 |  |
| HIST1H4A | -0.21573566 | 0.4985727 |  |
| AMBP | -0.21860381 | 0.812272 |  |
| FOS | -0.28383285 | 1.0121031 |  |
| ACTA1 | -0.32495032 | 1.0302624 |  |
| NTS | -0.33447668 | 1.0444431 |  |
| GNG8 | -0.37246544 | 0.9449922 |  |
| HIST1H4C | -0.48395839 | 0.9317957 |  |
| HIST1H4F | -0.49710568 | 0.268278 |  |
| SPTA1 | -0.50418527 | 1.0918021 |  |
| HIST1H2BH | -0.61697878 | 1.4035413 |  |
| ACTL8 | -0.67704829 | 0.991001 |  |
| HIST1H2BE | -0.69207976 | 0.9529295 |  |
| HIST1H2BG | -0.95227233 | 1.0496621 |  |
| ACACB | -1.04577153 | 0.9673898 |  |
| IGF2 | -1.05282594 | 0.8007452 |  |
| SPP1 | -1.10813384 | 2.0020723 |  |
| EGR1 | -1.21501582 | 0.969527 |  |
| DMD | -1.27120677 | 1.1995664 |  |
| HIST2H2BF | -1.31788402 | 1.1451191 |  |
| HIST1H4E | -2.24174547 | 1.1431484 |  |
