# Supplementary material for: Identification of Hub Genes Associated With Progression and Prognosis in Patients With Bladder Cancer
Source: Front Genet. 2019 May 7;10:408. doi: 10.3389/fgene.2019.00408 (PMC6513982; doi:10.3389/fgene.2019.00408)
Supplement: TABLE S3 — One-way ANOVA analysis, spearman correlation analysis, distance correlation analysis, and AUC of candidate hub genes. [file Table_3.DOC]

**Table S3: One-way ANOVA analysis, spearman correlation analysis, distance correlation analysis, and AUC of candidate hub genes.**

| **Gene ID** | **One-way ANOVA** | | **Spearman correlation** | | **Distance correlation** | | **ROC** |
| --- | --- | --- | --- | --- | --- | --- | --- |
|  | **F** | ***P*-value** | **Correlation** | ***P*-value** | **Correlation** | ***P*-value** | **AUC** |
| ACTN2 | 1.428 | 0.234 | 0.173 | 0.027 | 0.168 | 0.029 | 0.245 |
| BUB1B | 41.156 | 0 | 0.445 | 0 | 0.472 | 2.20E-16 | 0.934 |
| CCL2 | NA | NA | 0.367 | 0.000001 | 0.351 | 2.20E-16 | 0.153 |
| CCNB1 | 24.67 | 0.000002 | 0.37 | 0.000001 | 0.382 | 2.20E-16 | 0.884 |
| CDK1 | 32.094 | 0 | 0.392 | 0 | 0.408 | 2.20E-16 | 0.869 |
| CSF2 | NA | NA | 0.184 | 0.018 | 0.2 | 5.52E-04 | 0.785 |
| DCN | 4.83 | 0.029 | 0.145 | 0.063 | 0.161 | 4.18E-02 | 0.069 |
| FOXM1 | NA | NA | 0.513 | 0 | 0.545 | 2.20E-16 | 0.907 |
| HDAC4 | 0.016 | 0.898 | -0.051 | 0.519 | 0.102 | 5.98E-01 | 0.092 |
| HIST1H2AD | 3.144 | 0.078 | 0.157 | 0.043 | 0.181 | 6.05E-03 | 0.298 |
| HIST1H2BC | NA | NA | 0.39 | 0 | 0.385 | 2.20E-16 | 0.781 |
| HIST1H2BD | 18.112 | 0.000035 | 0.339 | 0.000009 | 0.344 | 2.20E-16 | 0.719 |
| HIST1H3A | 14.872 | 0.000165 | 0.303 | 0.000078 | 0.292 | 2.20E-16 | 0.708 |
| HIST1H3B | 10.181 | 0.002 | 0.294 | 0.000128 | 0.305 | 2.20E-16 | 0.786 |
| HIST1H3D | 10.877 | 0.001 | 0.257 | 0.001 | 0.25 | 1.16E-09 | 0.839 |
| HIST2H4A | NA | NA | 0.359 | 0.000002 | 0.365 | 2.20E-16 | 0.614 |
| ISG15 | 12.029 | 0.001 | 0.289 | 0.000169 | 0.295 | 2.20E-16 | 0.908 |
| KIF15 | 40.671 | 0 | 0.451 | 0 | 0.474 | 2.20E-16 | 0.888 |
| MMP9 | NA | NA | 0.329 | 0.000016 | 0.318 | 2.20E-16 | 0.555 |
| OIP5 | NA | NA | 0.402 | 0 | 0.449 | 2.20E-16 | 0.85 |
| PLK1 | NA | NA | 0.365 | 0.00001 | 0.406 | 2.20E-16 | 0.7 |
| RAD54L | 56.778 | 0 | 0.504 | 0 | 0.522 | 2.20E-16 | 0.951 |
| SHH | NA | NA | -0.332 | 0.000013 | 0.333 | 2.20E-16 | 0.386 |
| ZWINT | 37.495 | 0 | 0.44 | 0 | 0.463 | 2.20E-16 | 0.842 |
